# Enzyme Enhancement Through Computational Stability Design Targeting NMR-Determined Catalytic Hotspots

**DOI:** 10.1021/jacs.4c09428

**Published:** 2025-03-19

**Authors:** Luis I. Gutierrez-Rus, Eva Vos, David Pantoja-Uceda, Gyula Hoffka, Jose Gutierrez-Cardenas, Mariano Ortega-Muñoz, Valeria A. Risso, Maria Angeles Jimenez, Shina C. L. Kamerlin, Jose M. Sanchez-Ruiz

**Affiliations:** † Departamento de Química Física, Facultad de Ciencias, Unidad de Excelencia de Química Aplicada a Biomedicina y Medioambiente (UEQ), 16741 Universidad de Granada, Granada 18071, Spain; ‡ School of Chemistry and Biochemistry, 1372 Georgia Institute of Technology, Atlanta, Georgia 30332, United States; § Departamento de Química Física Biológica, Instituto de Química Física Blas Cabrera (IQF-CSIC), Madrid 28006, Spain; ∥ Department of Biochemistry and Molecular Biology, Faculty of Medicine, 37599 University of Debrecen, Debrecen 4032, Hungary; ⊥ Doctoral School of Molecular Cell and Immune Biology, University of Debrecen, Debrecen 4032, Hungary; # Department of Chemistry, Lund University, Lund 22100, Sweden; ¶ Department of Chemistry and Biochemistry, Kennesaw State University, Kennesaw, Georgia 30144, United States; ∇ Departamento de Química Orgánica, Facultad de Ciencias, Unidad de Excelencia de Química Aplicada a Biomedicina y Medioambiente (UEQ), Universidad de Granada, Granada 18071, Spain

## Abstract

Enzymes are the quintessential green catalysts, but realizing their full potential for biotechnology typically requires improvement of their biomolecular properties. Catalysis enhancement, however, is often accompanied by impaired stability. Here, we show how the interplay between activity and stability in enzyme optimization can be efficiently addressed by coupling two recently proposed methodologies for guiding directed evolution. We first identify catalytic hotspots from chemical shift perturbations induced by transition-state-analogue binding and then use computational/phylogenetic design (FuncLib) to predict stabilizing combinations of mutations at sets of such hotspots. We test this approach on a previously designed de novo Kemp eliminase, which is already highly optimized in terms of both activity and stability. Most tested variants displayed substantially increased denaturation temperatures and purification yields. Notably, our most efficient engineered variant shows a ∼3-fold enhancement in activity (*k*_cat_ ∼ 1700 s^–1^, *k*_cat_/*K*_M_ ∼ 4.3 × 10^5^ M^–1^ s^–1^) from an already heavily optimized starting variant, resulting in the most proficient proton-abstraction Kemp eliminase designed to date, with a catalytic efficiency on a par with naturally occurring enzymes. Molecular simulations pinpoint the origin of this catalytic enhancement as being due to the progressive elimination of a catalytically inefficient substrate conformation that is present in the original design. Remarkably, interaction network analysis identifies a significant fraction of catalytic hotspots, thus providing a computational tool which we show to be useful even for natural-enzyme engineering. Overall, our work showcases the power of dynamically guided enzyme engineering as a design principle for obtaining novel biocatalysts with tailored physicochemical properties, toward even anthropogenic reactions.

## Graphical abstract

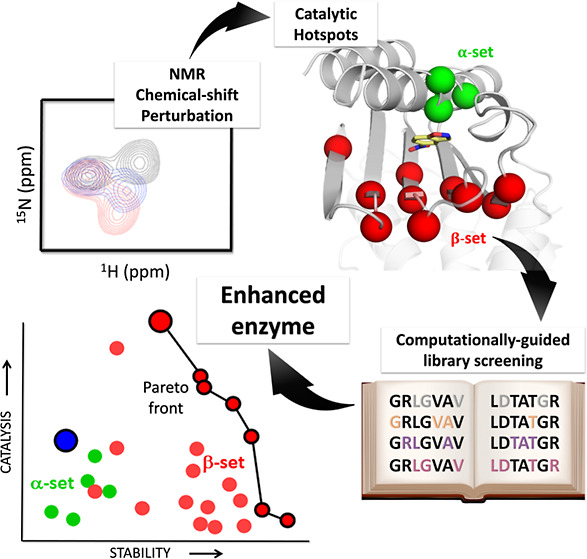

## Introduction

Enzymes have important and diverse applications as catalysts for organic synthesis, bioremediation and biochemical industrial reactions.^1,2^ Enzymes contribute in fact to superseding traditional industrial processes that were both often inefficient in terms of the use of natural resources and environmentally unfriendly in general, and overall promote sustainable chemistry.^3,4^ Most applications of enzymes require their optimization in terms of the relevant biomolecular properties. Developing methodologies for enzyme enhancement is therefore a goal with major economic impact. However, while increasing enzyme activity is obviously desirable for many industrial applications, so is ensuring sufficient enzyme stability. That is, low stability may facilitate irreversible enzyme denaturation and thus limit the total turnover of the enzyme under the conditions of the application. It may also simply preclude the use of high temperatures in the process, which may be required to warrant sufficient substrate solubility and to prevent microbial growth or simply to lead to even higher catalytic rates.^5,6^ Unfortunately, most mutations that are capable of modulating enzyme function are destabilizing,^7^ which explains why activity–stability trade-offs are often found in enzyme optimization studies.^8^ Certainly, it is plausible that a few mutations (or combinations of mutations) modulate activity without impairing stability. However, these mutations (or combinations thereof) are unlikely to be found through the screening of large random variant libraries. While this can to some extent be offset by the use of laboratory directed evolution, which is the most useful and broadly applicable methodology for protein engineering,^9^ this process is typically sluggish, and many rounds of time-consuming library screening are often required to reach the required levels of the targeted biomolecular properties.^10^ These challenges are obviously a reflection of the fact that the protein sequence space is vast and that most sequences do not encode for proteins with industrially desired biophysical properties. In view of this, considerable effort has been devoted in recent years to the development of general methodologies to design small focused libraries for guided directed evolution.^11–20^

In the present work, we show how the interplay between activity and stability in enzyme optimization can be efficiently addressed through the combination of two recently proposed methodologies to guide directed evolution.^18,19^ Our combined approach involves, first, the use of NMR to determine positions in the protein sequence at which mutations are likely to affect catalysis (catalytic hotspots). In this context, recent work^19^ has demonstrated that such catalysis hotspots can be identified from the chemical shift perturbations induced by the binding of a transition-state analogue to the targeted enzyme. This is important, as focusing directed evolution to catalytic hotspots bypasses one of the main factors that contributes to the ineffective screening of large random libraries, namely, that only certain regions of the enzyme structure are linked to the catalytic cycle and, therefore, that the screening of a random library involves the useless and time-consuming probing of large numbers of mutations that in fact do not affect catalysis. However, as we demonstrate in this work the process can be further optimized, by avoiding the preparation of random (saturation or combinatorial) libraries at the determined hotspots (as would be the standard practice). Rather, following recent work,^18^ we use FuncLib,^21^ a novel computational methodology that combines Rosetta design with phylogenetic analysis, to rank enzyme variants with multiple mutations according to predicted stability. This approach has been successfully used in a wide range of protein design applications.^21–26^ Specifically, we use here predictions from the FuncLib Web server to target sets of NMR-determined catalysis hotspots in such a way that the predicted variants may be expected to modulate enzyme activity without impairing stability. The use of FuncLib bypasses another major factor that contributes to making nonguided directed evolution inefficient, namely that since most mutations in a protein are destabilizing^27^ or even disruptive, screening of a random library with a significant mutational load involves the probing of variants with impaired stability which, in many cases, may not even fold properly.

As a protein system to test our combined approach, we have selected a de novo enzyme capable of catalyzing Kemp elimination through a classical proton-abstraction mechanism.^18,20,28^ Kemp elimination has been widely used as benchmark for de novo enzyme engineering.^18,20,28–33^ These studies obtained (at times substantial) improvement in Kemp eliminase activity over catalytically modest starting points. In contrast, in this work, the specific Kemp eliminase we are targeting here for further engineering is already substantially optimized in terms of both, stability and catalysis. That is, as a result of previous engineering efforts,^18,20,28^ it displays high catalysis levels, approaching in fact those of the best proton-abstraction Kemp eliminase to date which was the outcome of 17 rounds of directed evolution.^32^ Furthermore, it is a stable protein with a high denaturation temperature of 80 °C, reflecting in part the fact that the original scaffold for the engineering was a resurrected ancestral lactamase with high stability.^18,28^ Based on these facts, overall, further optimization of our target Kemp eliminase appears a priori difficult. Yet, we find that a rather limited screening of just 25 variants predicted by our combined NMR/computational approach leads to a variant with ∼3-fold improved activity (from an already very high starting point), with activities of the variants spanning a ∼50-fold range, and without impairing protein stability. In fact, most of the predicted variants display substantially enhanced denaturation temperatures and purification yields. Moreover, we reach a catalytic efficiency ∼4 × 10^5^ M^–1^ s^–1^ with a catalytic rate constant well above 1000 s^–1^, i.e. above most reported values for engineered de novo enzymes and modern natural enzymes,^4,34^ making our Kemp eliminase the most efficient engineered Kemp eliminase catalyzing this reaction through proton abstraction. Finally, molecular dynamics and empirical valence bond simulations of the optimized variants, compared to the “wild-type” GNCA4-W229/F290W^28^ and computationally optimized GNCA4-12 variants^18^ indicate the importance of conformational plasticity in creating a preorganized active site for efficient catalysis, both through conformational enrichment of catalytically productive conformations of the active site tryptophan, W290, which is in turn important for substrate stabilization in the active site, as well as through elimination of nonreactive substrate binding conformations. This mirrors observations from de novo designed Kemp eliminases^35,36^ but on an enzyme with a de novo active site generated using a minimalist design involving a single mutation,^28^ that may mimic the actual emergence of new enzymes during natural evolution. Finally, and remarkably, noncovalent interaction network analysis appears to be able to predict ∼50% of hotspots in both our designed Kemp eliminase as well as two naturally evolved proteins. Also, we demonstrate that further evaluation based on evolutionary conservation of interaction networks not only rationalizes experimental engineering outcomes but also acts as a tool to filter viable vs nonviable hotspot residue predictions from our interaction network analysis. Taken together, these tools provide a fully computational alternative to NMR for hotspot prediction for systems where NMR analysis is not viable.

## Results and Discussion

### De novo Kemp Eliminases Used in This Work

Kemp elimination (Figure 1A) is a simple reaction that has been used as a common benchmark in de novo enzyme engineering studies.^18–20,28–32,37^ As shown in Figure 1A, Kemp elimination is a classical model reaction for base-catalyzed proton abstraction from carbon, although there are increasing examples in the literature of redox mediated Kemp elimination.^19,33,38^ Kemp elimination is, in principle, an “easy” reaction that requires a very simple catalytic machinery, i.e., a catalytic base to remove the proton. Yet, it is not straightforward to reach high levels of catalysis for Kemp elimination, since the transition state (Figure 1A) is geometrically and electronically similar to the substrate and, therefore, achieving preferential stabilization of the former is challenging.^39^ This is for example evidenced by the only modest contribution of an oxyanion hole to catalysis in the highly proficient HG3.17 designed Kemp eliminase,^32^ as demonstrated by Kries and co-workers.^40^ Several Kemp eliminases from our previous work^18,20,28^ are used in this work to test our protein design workflow. These are summarized in Table S1 and described in more detail below.

**Figure 1:**
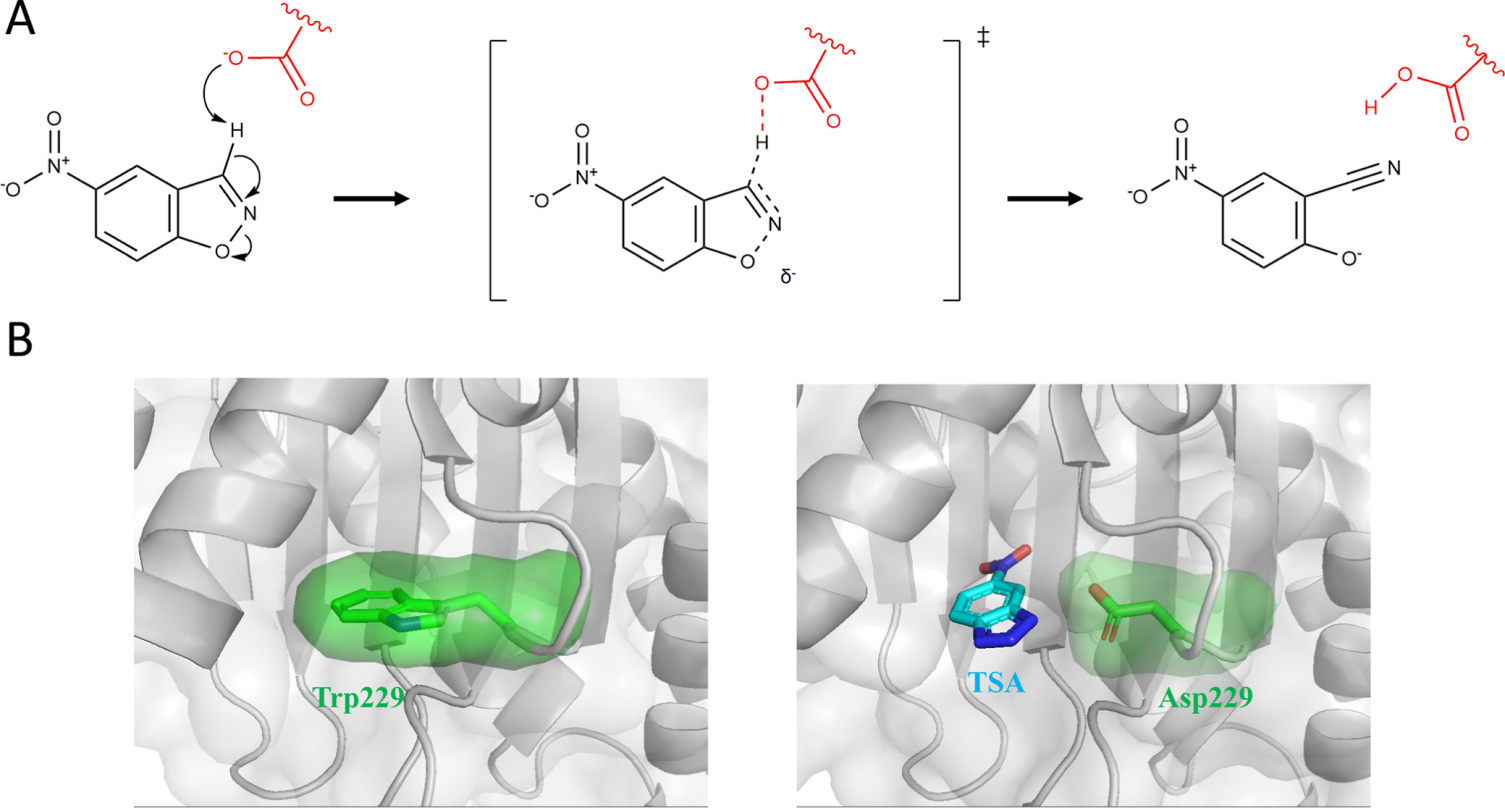
(A) Base-catalyzed Kemp elimination of 5-nitrobenzioxazole. A proposed structure for the transition state is shown. (B) Schematic representation of the minimalist approach used to generate a de novo active site for Kemp elimination. Replacing a partially buried tryptophan residue (left) in a β-lactamase scaffold with an aspartate generates a cavity with the approximate shape of the Kemp reactant and a catalytic base at the bottom of the cavity (right; note that a transition-state analogue is shown in the cavity).

As a starting point, we recently^28^ used a minimalist design strategy (Figure 1B) to generate in β-lactamase scaffolds a completely new active site capable of catalyzing Kemp elimination. This design approach involved replacing a large hydrophobic residue (a tryptophan) with an aspartate, in such a way that both, a cavity potentially capable to bind the Kemp substrate and a catalytic base potentially capable of performing proton abstraction, were generated through a single mutation. For this very simple design approach to be successful, the protein scaffold targeted for de novo active site generation must be able to tolerate a destabilizing disruptive mutation. Furthermore, since the cavity produced upon mutation does not exactly match the shape of the substrate molecule, the scaffold must be conformationally flexible, at least in the region of the new active site, in order to allow substrate binding, while at the same time not so flexible and solvent exposed that the substrate is unable to remain in the active site. Interestingly, while we tested a large number of modern (extant) and ancestral β-lactamases as scaffolds for potential Kemp elimination activity, the approach was only successful on proteins encoded by reconstructed ancestral β-lactamases sequences, obtained through ancestral sequence reconstruction.

Initially, the best Kemp eliminase obtained through this approach^28^ involved a single W229D mutation in the GNCA_MP_ scaffold, i.e., in the protein encoded by the most probabilistic sequence at the GNCA ancestral node (last common ancestor of various Gram-negative bacteria). This Kemp eliminase had a catalytic efficiency of 45 M^–1^ s^–1^ although its catalytic rate constant, *k*_cat_, could not be accurately determined. Further improvements in activity were achieved by introducing an additional mutation (F290W) at the de novo active site and by screening several alternative reconstructions at the GNCA node as scaffolds for engineering. This Kemp eliminase will henceforth be referred to as GNCA4-WT, as it provided the baseline variant for our subsequent optimization effort.^18^ It is important to note that the same design approach did not generate Kemp eliminase activity when implemented on 10 different modern β-lactamases of moderate stability that were conformationally rigid.^28^ Introduction of the additional F290W mutation let us reach a catalytic efficiency of ∼5 × 10^3^ M^–1^ s^–1^ with a catalytic rate constant as high as ∼10 s^–1^ for Kemp elimination by GNCA4-WT.^28^ It is also worth noting that the natural activity of β-lactamases is the degradation of β-lactam antibiotics, such as penicillin.^41,42^ Our design approach generated a de novo active site that is both chemically distinct and spatially separated from the natural antibiotic degradation active site.

We then further optimized GNCA4-WT^28^ using the FuncLib server^21^ on the basis of the screening of a small ultrafocused library randomizing 11 positions, that included variants with multiple active-site mutations at the top of the stability ranking predicted by FuncLib. This resulted in a Kemp eliminase with a catalytic efficiency of ∼2 × 10^4^ M^–1^ s^–1^, a catalytic rate constant of ∼100 s^–1^ and a denaturation temperature of 76 °C, as determined by scanning calorimetry. We shall refer to this variant as GNCA4-12, in line with our prior work.^18^ Finally, GNCA4-12 was further enhanced on the basis of the inclusion of an extra polypeptide segment at the carboxyl terminus (Figure S1). The rationale behind this approach was that the extra polypeptide segment could generate new interactions at the adjacent de novo active site and that these interactions could perhaps be tapped for catalysis enhancement. In order to do so, a library including all combinations of the 20 amino acids at three positions in the extra segment was prepared. This combinatorial library spanned 8000 variants, of which ∼800 were screened for Kemp elimination activity. The best variant thus obtained had a catalytic efficiency of ∼2 × 10^5^ M^–1^ s^–1^, a catalytic rate constant of ∼600 s^–1^ and a denaturation temperature of 80 °C. We shall refer to this variant simply as V4, following the terminology used in the original work.^20^ Note that the approach based on the inclusion of an additional polypeptide segment does not rely on features specific to Kemp elimination or to the protein scaffolds we use for de novo enzyme engineering. It appears thus reasonable that the approach is general to a substantial extent, although additional studies on other enzyme systems will be required to confirm this statement.

This Kemp eliminase variant, V4, resulting from previous studies described above, provides the starting point for the current work. It is already highly optimized in terms of both stability and catalysis. Its denaturation temperature value, 80 °C, is, for instance, much higher than that for the prototypical TEM1 β-lactamase (55 °C).^42^ Regarding de novo Kemp eliminase catalysis on this scaffold, the catalytic parameters for V4 (*k*_cat_ ∼ 600 s^–1^ and *k*_cat_ */K*_M_ ∼ 2 × 10^5^ M^–1^ s^–1^) are similar to those of the best proton-abstraction Kemp eliminase reported to date (HG3.17 *k*_cat_ = 700 s^–1^, *k*_cat_/*K*_M_ = 2.3 × 10^5^ M^–1^ s^–1 32^), which was the result of 17 rounds of directed evolution starting with a rationally designed background with a low activity. We note here that a recent even more efficient Kemp eliminase has been designed with a catalytic rate constant of ∼3600 s^–1^; however, this enzyme does not use a conventional proton-abstraction mechanism and is a redox-mediated enzyme based on using myoglobin as a starting point.^19^ It is clear overall that further optimization of V4 appears a priori to be a challenging prospect. This challenge is why we chose the V4 variant as an engineering target to stress-test our approach. Experiments performed as part of the current study and aimed at further enhancing the V4 variant are described below.

### Determination of Catalysis Hotspots from NMR Chemical Shift Perturbation Experiments

Recent work^19^ has suggested that catalytic hotspots for focusing directed evolution experiments can be determined from NMR spectroscopy. The rationale behind this proposal is that, since enzyme action is based upon transition state stabilization, residue positions linked to catalysis will display changes in chemical shift upon the binding of a transition-state analogue. The approach has been shown to have the potential to identify even hotspots that are distant from the active site, and, due to this distance, are likely modulating activity through dynamical effects that are not apparent in the static 3D-structure.

Our goal in this work is to use NMR-guided directed evolution combined with computationally focused library screening to optimize the V4 Kemp eliminase (the best Kemp eliminase from our previous work^20^). As our starting point, we initially aimed at obtaining NMR chemical shift perturbations (CSP) with this variant. However, the inclusion of the extra polypeptide segment (Figure S1) in this variant makes it prone to slowly aggregate in solution, which makes V4 unsuitable for long-time NMR experiments. As a substitute for this, we performed NMR experiments and subsequent computational optimization using the GNCA4-12 Kemp eliminase, under the reasonable assumption that the hotspots thus determined could also be used for V4 optimization due to the sequence similarity between the two enzymes (which only differ in the presence of the additional segment in V4). Following previous work in the field, we used 5(6)-nitrobenzotriazole (Figure 2A) as an analogue of the transition state for Kemp elimination.

**Figure 2:**
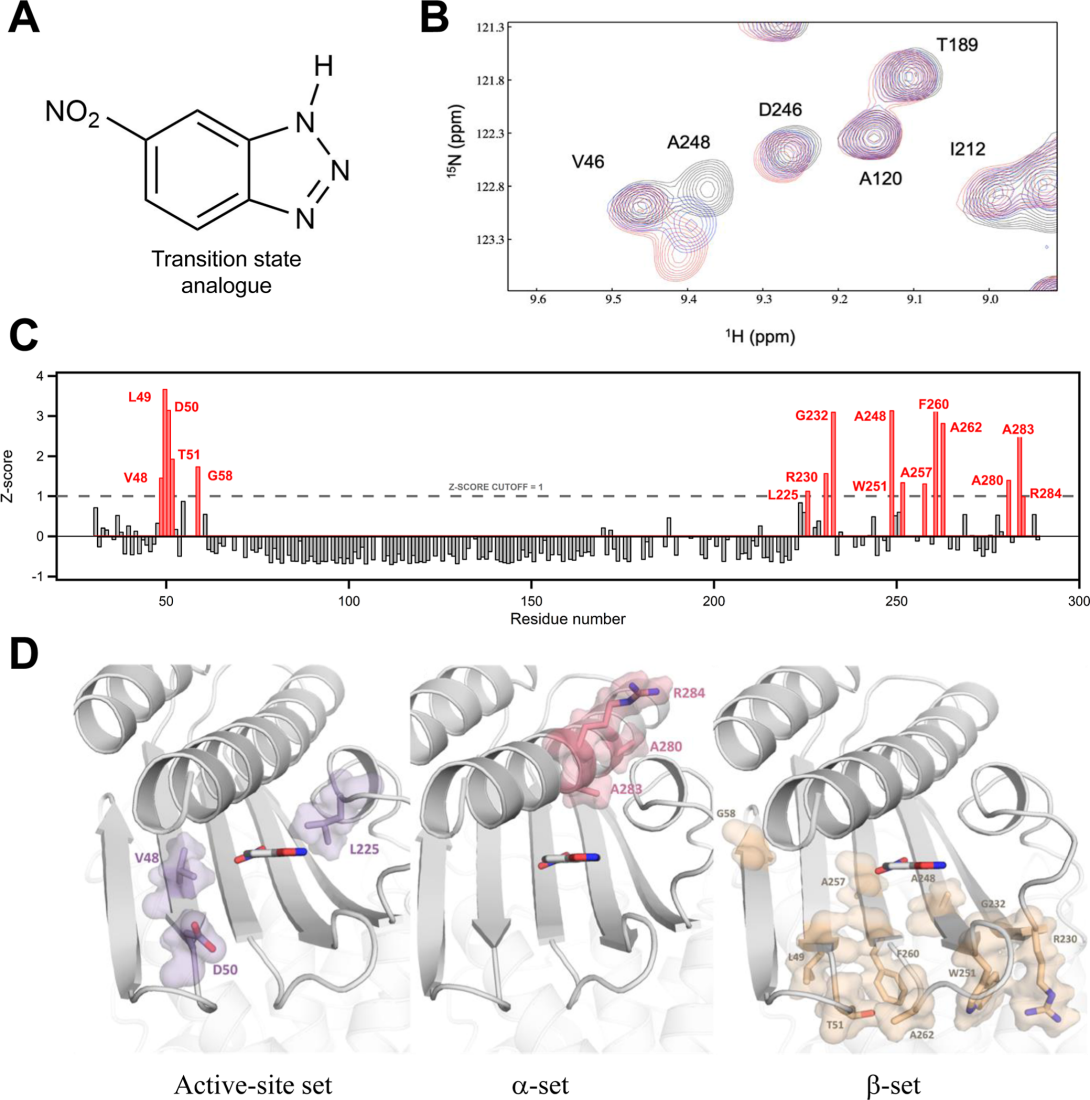
(A) 5(6)-nitrobenzotriazole, the transition-state analogue used in this work. (B) Representative example of perturbation of ^1^H, ^15^N HSQC spectra upon transition-state analogue binding. The spectrum of unliganded GNCA4-12 is shown (black contours), together with spectra of GNCA4-12 in the presence of the transition-state analogue at the following protein/analogue ratios: 1:0.1, 1:0.14; 1:0.2 (blue contours); 1:1.1 and 1:2.1 (red contours). Some cross-peaks are labeled. The strong perturbation of the cross-peak assigned to A248 is visually apparent. (C) CSP Z-scores as a function of residue number. Bars corresponding to CSP Z-scores ≥1 are colored in red. (D) Structural sets defined by the 16 hotspots from our NMR chemical shift perturbation experiments that could be unequivocally assigned to specific residue positions. The positions in each set are shown and labeled. The location of the de novo active site is indicated by bound transition-state analogue.

As described by Korendovych and colleagues,^19^ we quantified chemical shift perturbations by calculating Z-scores from the differences in the ^1^H_N_ and ^15^N chemical shifts of the GNCA4-12 Kemp eliminase upon binding the transition-state analogue (see the Experimental Section for details). This requires the unequivocal assignment of all the cross-peaks present in the 2D ^1^H, ^15^N HSQC spectrum of the free GNCA4-12 Kemp eliminase (see Figure S2) followed by experiments in the presence of increasing amounts of the transition state analogue. In the case of the unligated GNCA4-12 Kemp eliminase, these assignments were done by comparison with the related and previously assigned GNCA_MP_ Kemp eliminase variant^28^ combined with the analysis of triple resonance experiments (3D HNCO and 3D HNCA, see the Experimental Section). In this way, the chemical shifts for the backbone ^1^H_N_, ^15^N, ^13^C′ and ^13^C_α_ atoms (listed in Table S2) were obtained for about 78% of the protein residues, except for the 12 prolines, the 4 *N*-terminal residues and 5 *C*-terminal residues. Cross-peaks for the latter terminal residues could not be unequivocally identified likely because signal overlap and/or broadening or disappearance caused by exchange of the ^1^H_N_ amide protons with the water. The helices and strands deduced from the chemical shifts are in concordance with those present in the crystalline structure of the GNCA_MP_ variant^28^ (Figure S3), supporting that the GNCA4-12 variant maintains the structure characteristic of our previous Kemp eliminases based on ancestral lactamase scaffolds.

Upon stepwise addition of the transition state analogue, most cross-peaks in the 2D ^1^H, ^15^N HSQC spectra of GNCA4-12 either remain unaffected or slightly shifted, having CSP Z-scores lower than unity. A number of cross-peaks were gradually shifted, as illustrated with the residue A248 in Figure 2B. Such gradual shifts indicate that the binding of the transition state analogue occurs in the fast exchange regime. CSP Z-scores can be determined and unequivocally attributed to specific residues and thus to specific locations in the 3D-structure, provided that the cross-peaks are assigned in both the free GNCA4-12 and GNCA4-12 with the bound transition-state analogue. Out of 256 nonproline amino acid residues present in the GNCA4-12 scaffold (note that prolines are not observable in ^1^H, ^15^N HSQC spectra), a total of 189 CSP Z-scores could be unequivocally identified. Their position in the GNCA4-12 sequence is shown in Figure 2C. Sixteen of these assigned *Z* scores were ≥1 and were thus considered to identify potential catalysis hotspots. Hence, we decided to focus experimental protein engineering to these 16 positions.

### Structural Description of NMR-Determined Catalysis Hotspots

Remarkably, the 16 hotspots from our NMR chemical shift perturbation analysis that could be unequivocally assigned to specific residue positions conform to clear structural patterns on the protein scaffold. In fact, as described below in some detail, they can be grouped in three different sets:

The three hotspots at positions 48, 50, and 225 are located close to the transition-state analogue in a 3D-structure of the GNCA4-12 variant (Figure 2D). These positions are effectively located at the Kemp elimination active site, and we will thus refer to them collectively as the “active-site set”.

The three hotspots at positions 280, 283, and 284 are located in the long 271–289 α-helix. Position 289 at the end of the helix is the original location of the carboxyl terminus, although in most variants a tail for purification or for activity enhancement (in the V4) has been attached there. Position 289 and neighboring residues are reasonably close to the de novo active site. However, the hotspot positions 280, 283, and 284 appear close to each other in a section of the α-helix that is removed from the Kemp elimination active site. Chemical shift perturbations (high *Z* scores) at these positions upon binding of a transition state analogue suggests, therefore, long-distance communication linked to catalysis. We will refer to positions 280, 283, and 284 collectively as the “α-set”.

The ten hotspots at positions 49, 51, 58, 230, 232, 248, 251, 257, 260, and 262 form a well-defined cluster with interesting structural features. All of them are located in the β-strand that forms a “wall” of the de novo active site. Therefore, many of them could be considered as “active site” residues, except for the fact that in all cases the side chains are pointing in the opposite direction, i.e., away from the active site (Figure 2D). Chemical shift perturbation of these residues upon binding of transition-state analogue may simply reflect small changes in the shape of the de novo active site. For this reason, mutations at these hotspot positions, even if they do not involve active-site side chains, may plausibly alter the active-site shape and in doing so affect catalysis. We will refer to positions 49, 51, 58, 230, 232, 248, 251, 257, 260, and 262 collectively as the “β-set”.

### Exploration of the Active-Site Hotspot Set for Kemp Elimination Activity

Effects of mutations at the active site on enzyme activity may be related with very specific interactions of the mutated side chains. Therefore, we deemed convenient to perform screening of saturation mutagenesis libraries at the three positions of the active-site set, 48, 50, and 225 (Figure S4). We did not find any significant enhancement in Kemp eliminase activity at either of the three positions, a result which supports that the active site region has been already optimized to a large extent in previous engineering efforts.

### Exploration of the α-set for Kemp Elimination Activity

Following our previous success with this approach, we used the FuncLib Web server to computationally search the sequence space corresponding to the three hotspot positions of the α-set. The residues at the hotspot positions in the background V4 variant are A280, A283 and R284. FuncLib identified only a limited available sequence space as being available at those positions (Table S3 and Supporting Information Table 1): alanine was fixed at position 280; alanine and threonine were allowed at position 283, and alanine, lysine and arginine at position 284. More importantly, FuncLib predicted that all variants in the available sequence space for the α-set were destabilizing, with worse Rosetta scores than the initial sequence by between 4 and 10 kcal mol^–1^ (Table S4 and Supporting Information Table 1). Despite this, we decided to prepare and determine the Kemp eliminase activity of these variants in order to test prediction of the NMR chemical shift perturbation experiments. The five variants displayed substantially diminished Kemp elimination activity with respect to the V4 background (Figure S5 and Table S5). Clearly, the screening of the α-set on the basis of FuncLib predictions does not lead to enzyme optimization. It is important to note, however, that it does confirm the capability of chemical shift perturbation experiments to identify long-distance communication pathways that are relevant to enzyme catalysis, even if in this case the impact of perturbing those pathways was detrimental.

### Exploration of the β-set for Kemp Elimination Activity

The β-set includes 10 hotspot residues, many of them located in the active-site β-strand but with their side chains pointing away from the active site. Position 260 in this set was optimized to some substantial extent in our previous work.^18^ Therefore, we focused our FuncLib analysis to the remaining nine hotspots. Unlike the case of the α-set described above, FuncLib found that the available sequence space for the β-set was comparatively broad (Table S6) and that many of the variants in that sequence space were predicted to be stabilizing, at least by a few kcal mol^–1^, based on calculated Rosetta scores (Supporting Information Table 2). We prepared and determined Michaelis profiles of rate vs substrate concentration for the 20 variants at the top of the predicted stability ranking (i.e., the 20 top variants in Supplementary Table 2; see also Table S7). Remarkably, we found a large (∼50-fold) range of modulation in rate across the designed variants (Figure 3), thus confirming the prediction of our chemical shift-perturbation NMR experiments that positions bearing side chains outside the active site may affect catalysis upon mutation.

**Figure 3:**
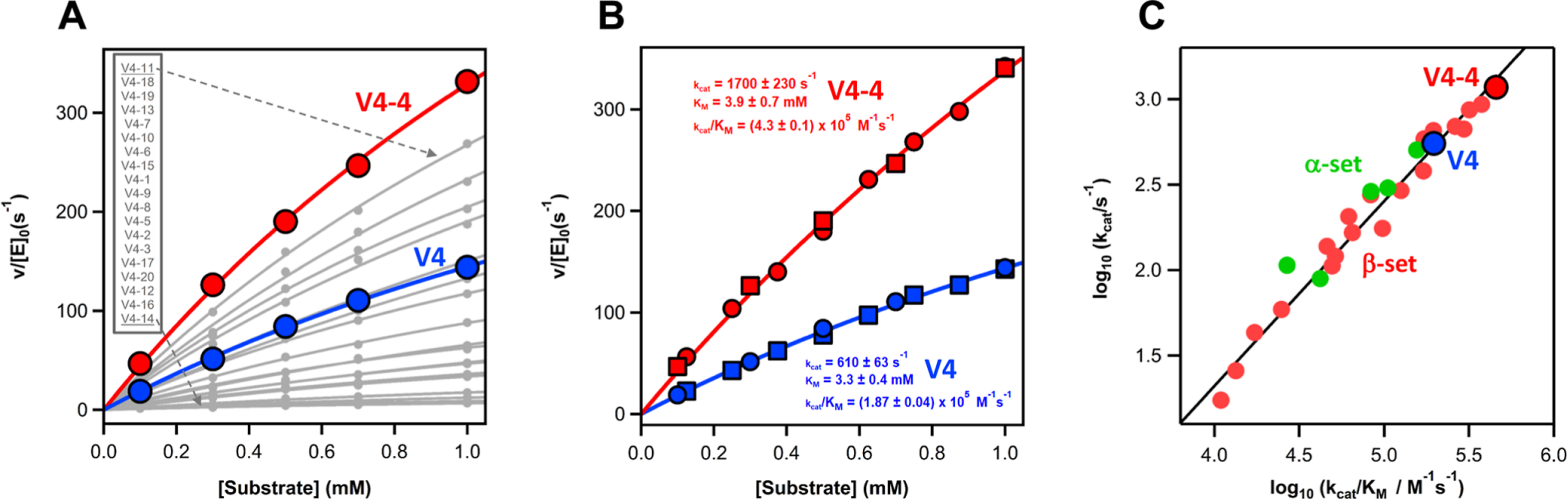
(A) Plots of rate vs substrate concentration for the variants predicted by the FuncLib Web server (Table S7) for the β-set (Figure 2). [E]_0_ is the total enzyme concentration and *v* stands for the initial rate. The continuous lines represent the best fit of the Michaelis–Menten equation. The profiles for the V4 variant used as background and for the best variant, V4-4, are highlighted in blue and red. (B) Plots of rate vs substrate concentration for the background and best variants, V4 and V4-4. For each variant data obtained with two different protein preparations are shown (circles and squares). The lines are the best fits of Michaelis–Menten equation. The values shown for the catalytic parameters are derived from fits to the combined data sets. However, essentially the same values within the associated uncertainties were obtained from individual analyses. Specifically, for V4-4 we obtained *k*_cat_ = 1711 ± 267 s^–1^ and *k*_cat_/*K*_M_=(4.2 ± 0.1)·10^5^ M^–1^ s^–1^ from the individual fit to one set and *k*_cat_ = 1529 ± 293 s^–1^ and *k*_cat_/*K*_M_=(4.4 ± 0.1)·10^5^ M^–1^ s^–1^ from the individual fit to the other set. The values we obtained for V4 are *k*_cat_ = 663 ± 96 s^–1^ and *k*_cat_/*K*_M_=(1.83 ± 0.06)·10^5^ M^–1^ s^–1^ from the individual fit to one set and *k*_cat_ = 551 ± 293 s^–1^ and *k*_cat_/*K*_M_=(1.96 ± 0.04)·10^5^ M^–1^ s^–1^ from the individual fit to the other set. (C) Plot of logarithm of catalytic rate constant vs logarithm of catalytic efficiency including the background V4 protein, and the variants based on the α-set and the β-set that have been studied experimentally in this work. The excellent linear correlation observed supports that ∼50-fold range in catalytic efficiency seen in our designs results mainly from changes in catalysis, rather than from changes in substrate binding. All data in this figure were obtained at pH 8.5.

Several features of the large range of rates achieved across our variants are worth noting:

1. Although many of the variants display diminished activity compared with the background V4 variant, several variants are significantly more active (Figure 3). In particular, variant V4-4 shows, at pH 8.5, a catalytic efficiency, *k*_cat_ */K*_M_, of (4.3 ± 0.1)·10^5^ M^–1^ s^–1^, and a catalytic rate constant, *k*_cat_, of 1700 ± 230 s^–1^. We emphasize that this is actually a very high *k*_cat_ value that is above most reported *k*_cat_ values for engineered/evolved de novo enzymes and modern natural enzymes.^4,34^

2. The activity range across the designed variants (which spans impaired and improved activity) is ∼50-fold. It is important for design purposes to emphasize that this ∼50-fold change in activity is not achieved through active site redesign or through impairment of the catalytic machinery. Rather, it is achieved through mutating side-chains that are outside the active-site, and this difference is significant. Certainly, nonactive-site mutations could impair activity if they are disruptive and prevent proper folding to the functional 3D-functional structure. However, our combinations of mutations are not disruptive: they are stabilizing, in fact, as shown in Figure 4. Therefore, the reported 50-fold modulation in rate supports that large effects on activity may be achieved by targeting distant hotspots, which is an important and nonintuitive result. The overall activity enhancement with respect to the background variant used in our study is smaller, at ∼3-fold, but this only reflects our choice of selecting as background an already highly active enzyme that had been previously extensively optimized (larger enhancements could have likely been obtained with a less optimized background).
3. Over the ∼50-fold modulation range, there is a good correlation between the catalytic efficiency, *k*_cat_ */K*_M_, and the catalytic rate constant, *k*_cat_, meaning that the modulation reflects changes in catalysis, rather than changes in substrate binding. This is relevant because, while a high value of the catalytic efficiency can be in principle achieved on the basis of tight substrate binding (i.e., a low *K*_M_ value), many practical applications of enzymes employ comparatively high substrate concentrations at which the rate is mainly determined by the *k*_cat_ value. Enzyme optimization approaches are therefore expected to specifically target the catalytic rate constant.^43^
4. The large ∼50-fold modulation in rate upon introducing mutations is not accompanied by impairment in stability. The opposite is true in fact. All the 20 variants tested are more stable than the background V4 variant, as inferred from the denaturation temperature values determined by differential scanning calorimetry (Figures 4 and S6), revealing, therefore, the absence of stability/activity trade-offs for the studied set. Furthermore, several variants display both enhanced stability and catalysis and the Pareto fronts^44^ of optimum stability/catalysis solutions are shown in Figure 4 (see Supplementary Discussion for details). Certainly, the overall observed enhancements with respect to the background used are moderate. Yet, these enhancements are relevant because we purposedly selected a very difficult target for optimization. Our background scaffold thus comes from ancestral sequence reconstruction, a methodology known to lead in many cases to proteins with enormously enhanced stability, and shows, in fact, a denaturation temperature many degrees above that of a modern homologue. Furthermore, our background already displays a level of activity toward Kemp elimination similar to the best proton-abstraction Kemp eliminase previously reported in the literature, which required 17 rounds of optimization by directed evolution, and has a *k*_cat_ of 700 s^–1^ and a *k*_cat_/*K*_M_ of 2.3 × 10^5^ M^–1^ s^–1^. Against this backdrop, the absolute activities obtained in this work are tremendous. Our best engineered variant has a *k*_cat_ of 1700 s^–1^, and a *k*_cat_/*K*_M_ of 4.3 × 10^5^ M^–1^ s^–1^ and it is not impaired in terms of stability. Rather, its denaturation temperature is a few degrees higher than that of the highly stable background used.
5. Most Kemp eliminase variants show a more efficient expression as compared with the V4 background, as least as reflected in the amount of protein obtained (Table S8). This result is interesting because expression level and purification yields are biotechnologically relevant parameters which may reflect not only stability but also folding rate and, more generally, the folding landscape.^45^

**Figure 4:**
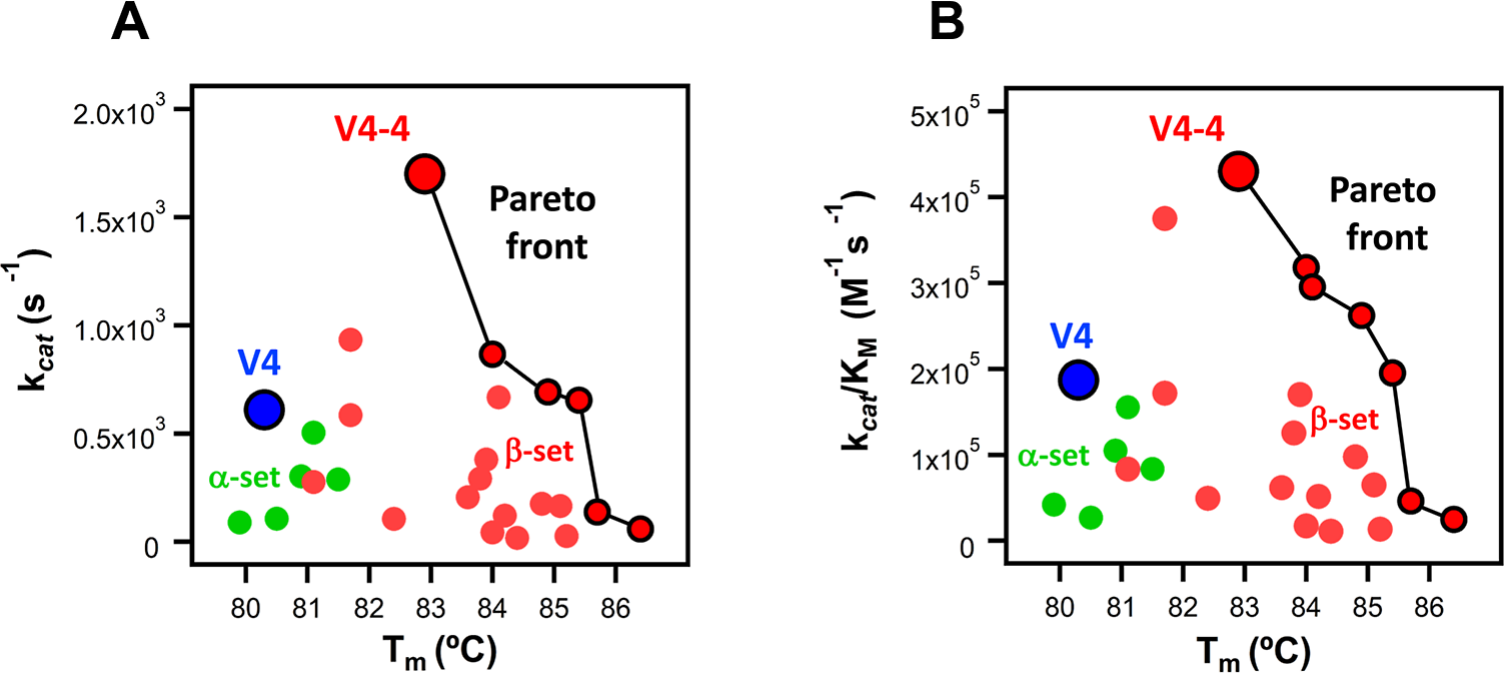
Plots of catalytic rate constant (A) and catalytic efficiency (B) vs denaturation temperature including the background V4 protein, and the variants based on the α-set and the β-set that have been studied experimentally in this work. Denaturation temperature values were determined by differential scanning calorimetry (Figure S6). The plots reveal the absence of a stability–activity trade-off. The lines connect the data for the nondominated variants, i.e., the Pareto subset or Pareto front, which represents the set of optimum solutions for a multiobjective optimization problem (see Supplementary Discussion for details). A variant is nondominated if no other variant is better in terms of, simultaneously, all the features targeted for optimization (activity and stability in this case).

### pH-Dependence of the De Novo Catalysis

In order to gain further insight into the origin of the obtained enhancement in catalysis, we have determined Michaelis profiles of rate vs substrate concentration at different pH values for, both the V4 background variant and the V4-4 variant (Figure S7) which shows the highest level of catalysis among the variants derived from the β-set. In principle, the pH-dependence of the rate parameters (Figure 5 and Table S9) is expected to be determined by the protonation of the catalytic base, which in our case is the aspartate side chain. Normal (unperturbed) p*K*_a_ values for aspartic acid are about 4. Therefore, any significant pH effect on rates observed at pH values above 6 (as in Figure 5) necessarily implies highly perturbed p*K*_a_ values for the active-site aspartic acid residues. This is, of course, reasonable as a buried aspartate in a mostly hydrophobic cavity is expected to display a perturbed p*K*_a_ value substantially above 4, and elevating the p*K*_a_ value of the catalytic base (e.g. through desolvation) is a common engineering strategy to enhance catalysis in Kemp eliminases and other enzymes. As discussed below, while the pH-dependence of the catalytic efficiency can be reasonably explained assuming a single perturbed p*K*_a_ value, that of the catalytic rate constant points to an ensemble of perturbed p*K*_a_ values, in congruence with the computational analyzed reported further below which suggest enhanced conformational diversity in the Michaelis complex.

**Figure 5:**
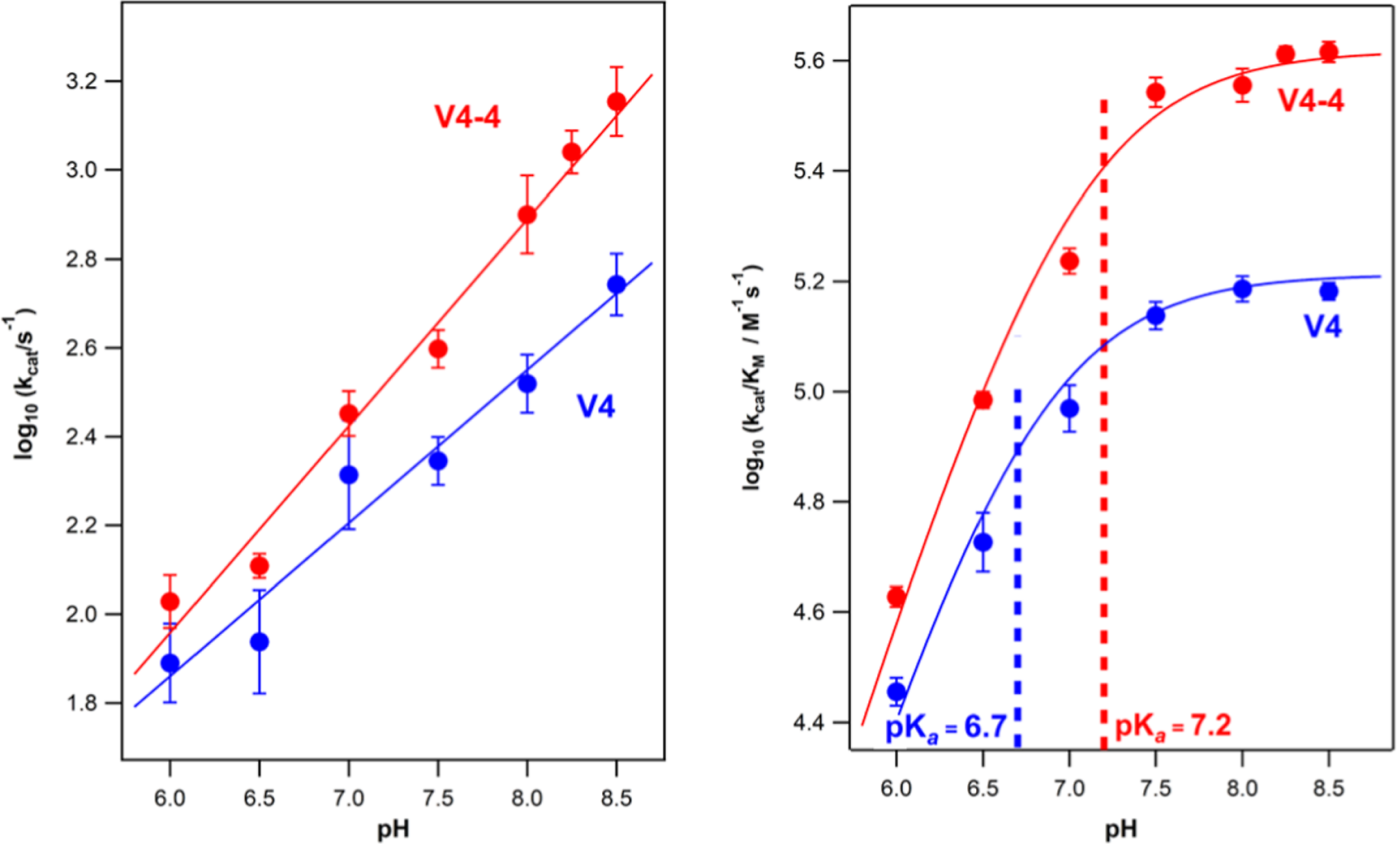
Effect of pH on the catalytic parameters for the best Kemp eliminase achieved in this work (variant V4-4) and the background protein V4. Values of catalytic rate constant and catalytic efficiency have been determined from the fitting of the Michaelis–Menten equation to profiles of rate vs substrate concentration obtained at different pH values (Figure S7). Error bars are standard deviations from the fittings of the Michaelis–Menten equation and are no not shown when they are smaller than the size of the data points. The dependence of logarithm of the catalytic rate constant with pH (left) is linear. Profiles of logarithm of catalytic efficiency vs pH are well described by eq 1 in the main text; fittings are shown with continuous lines in panel on the right. The p*K*_a_ of the catalytic aspartic acid residue is shown. Note that the change in trend in the plot occurs at pHs close to the p*K*_a_ values (see vertical dashed lines).

A simple theoretical analysis based on the pre-equilibrium interpretation of the Michaelis–Menten equation (see the Supplementary Discussion) suggest the following equation

1 $$\log_{10}P=\log_{10}P_{0}+\log_{10}\frac{10^{\left(pH-{pK}_{a}\right)}}{1+10^{\left(pH-{pK}_{a}\right)}}$$

where *P* is a catalytic parameter (either the catalytic efficiency, *k*_cat_ */K*_M_, or the catalytic rate constant, *k*_cat_), *P*_0_ is the value of the catalytic parameter at a sufficiently high pH and p*K*_a_ refers specifically to the p*K*_a_ of the (conjugate acid of the) catalytic base. This equation assumes a single p*K*_a_ value for the catalytic base, which, when *P* is catalytic efficiency, would describe ionization in the free enzyme while, when *P* is the catalytic rate constant, would describe ionization in the Michaelis complex (see the Supplementary Discussion).

Equation 1 predicts that, for pH ≫ p*K*_a_, the catalytic parameter will reach a plateau, log_10_ *P* = log_10_ *P*_0_, while, for pH ≪ p*K*_a_, log_10_ *P* = log_10_ *P*_0_ + pH – p*K*_a_, and the dependence of the logarithm of the catalytic parameter with pH is a line of slope unity. Consequently, according to eq 1 the p*K*_a_ value should be visually apparent in profiles for the pH-dependence of the logarithm of rate parameter as the approximate pH value at which the change of trend is observed (i.e., the pH value for the “kink” in the profile). The catalytic efficiency vs pH profiles can be in fact adequately described by eq 1 with a perturbed p*K*_a_ of about 7, as shown in the left panel of Figure 5. On the other hand, the profiles for the pH-dependence of the logarithm catalytic rate constant do not show a plateau but are reasonably well described by straight lines (Figure 5). It would seem that the p*K*_a_ of the catalytic base in the Michaelis complex is highly perturbed and clearly above the studied pH range (i.e., above 8.5). However, such interpretation in terms of a single p*K*_a_ value does not hold because the slopes of the log_10_ *k*_cat_ vs pH lines are clearly below unity. In fact, the single-p*K*_a_ eq (eq 1) does not provide a satisfactory fit to the experimental *k*_cat_ vs pH data, as shown in Figure S8. A more reasonable interpretation is perhaps that the Michaelis complex exist in an ensemble of conformations with different p*K*_a_ values (see the Supplementary Discussion). Of course, there may be other factors that could contribute to the seemingly anomalous of the pH-dependence of *k*_cat_, among that the equilibrium of formation of the Michaelis complex is not established. Yet, the contribution of an ensemble of conformations in the Michaelis complex is reasonable and it is supported by our molecular dynamics simulations (see “Computational characterization of the altered catalytic activity of the Kemp eliminase variants”). That is, the p*K*_a_ of buried aspartic acid residues are expected to be increased with respect to the model value of about 4 due to the hydrophobicity of the environment, but the p*Ks* corresponding to the different conformations may be increased to different extents thus leading to the failure of the single-p*K*_a_ model to describe the *k*_cat_ vs pH profiles.

### Computational Characterization of the Altered Catalytic Activity of the Kemp Eliminase Variants

To computationally probe the molecular origins for the enhanced chemical activities of the GNCA4-WT, GNCA4-12, V4-4 variants, as our starting point, we performed molecular dynamics simulations of each variant in complex with the substrate 5-nitrobenzisoxazole, as described in the Experimental Section. In the case of the GNCA4-12 variant, we also performed simulations for its V4 variant, which presents an additional polypeptide segment,^20^ in order to probe the impact of this polypeptide segment on catalysis. Analysis of these simulations give rise to two major observations, that align well with prior studies of de novo designed Kemp eliminases. The first of these (Figure 6) is that the W290 side chain, which was inserted into the GNCA4-WT to improve stability and activity, is actually conformationally diverse and can take on a range of different side chain conformations. Upon optimization of the eliminase, in the V4-4 variant, we observe two discrete metastable conformations of the W290 side chain with a clear loss of a third (likely catalytically unfavorable) conformation of the tryptophan observed in GNCA4-WT. In this conformation, the tryptophan points out of the active site and away from the substrate (conformation I, Figure 6). Importantly, the addition of the extra peptide from GNCA4-12 to V4 variant further results in a decrease in mobility of the W290 side chain, which might be related with the catalytic improvement of the enzyme. This is analogous to similar observations in the de novo designed Kemp eliminase KE07, where evolutionary conformational selection allowed for the emergence of multiple active site conformations (including multiple conformations of a key active site tryptophan) during directed evolution of this enzyme.^35^ However, in the case of KE07, this was a de novo active site based on a computational design involving a substantial number of mutations; in the current case, we now observe this effect on an enzyme with a de novo active site generated using a minimalist design involving a single mutation, that may mimic the actual emergence of new enzymes during natural evolution.^28^

**Figure 6:**
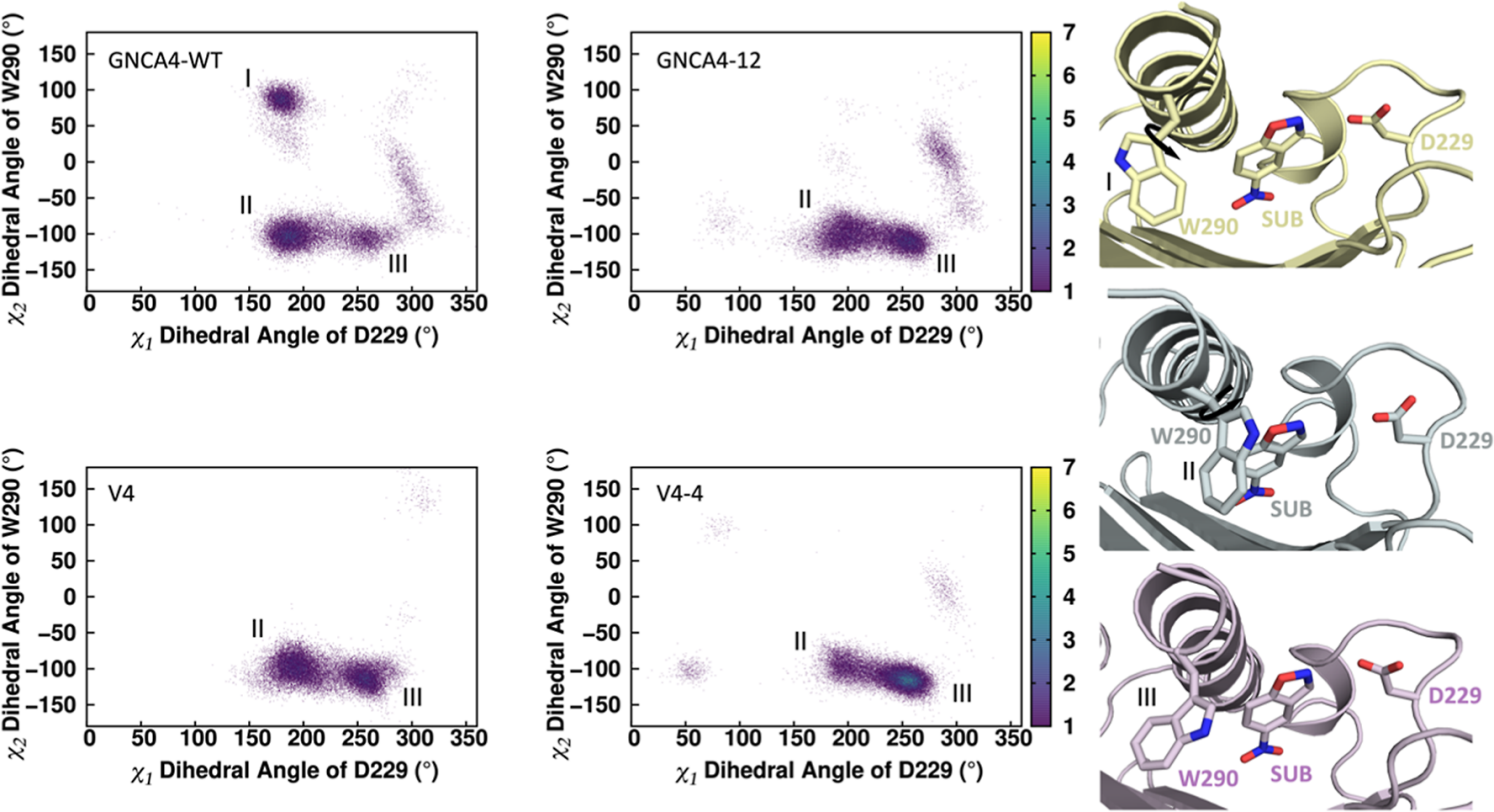
Joint distribution of the conformational space sampled by the χ_1_ dihedral angle of the D229 side chain, and the χ_2_ dihedral angle of the W290 side chain, in simulations of the GNCA4-WT, GNCA4-12, V4 and V4-4 variants. As can be seen from this data, in the GNCA4-WT variant, we observe three distinct metastable conformations of the W290 side chain, which are illustrated in the panel to the right (conformation I in gold at the top, II in gray in the middle, and III in purple at the bottom).

The second crucial observation from our simulations is the conformational flexibility of the substrate (Figure 7). Our de novo active site is built into a hydrophobic pocket originally evolved to stabilize a hydrophobic tryptophan side chain.^28^ While there is shape congruence between the substrate for Kemp elimination and the tryptophan side chain, the polar NO_2_ group of 5-nitrobenzisoxazole is unlikely to want to remain in this hydrophobic pocket. In fact, we see that the ligand gets pushed out of the active site for part of the simulation time, taking both an “IN” conformation which is similar to the conformation originally observed in the GNCA4-WT in complex with a transition state analogue, and an “OUT” conformation where the NO_2_ group points out of the hydrophobic pocket.

**Figure 7:**
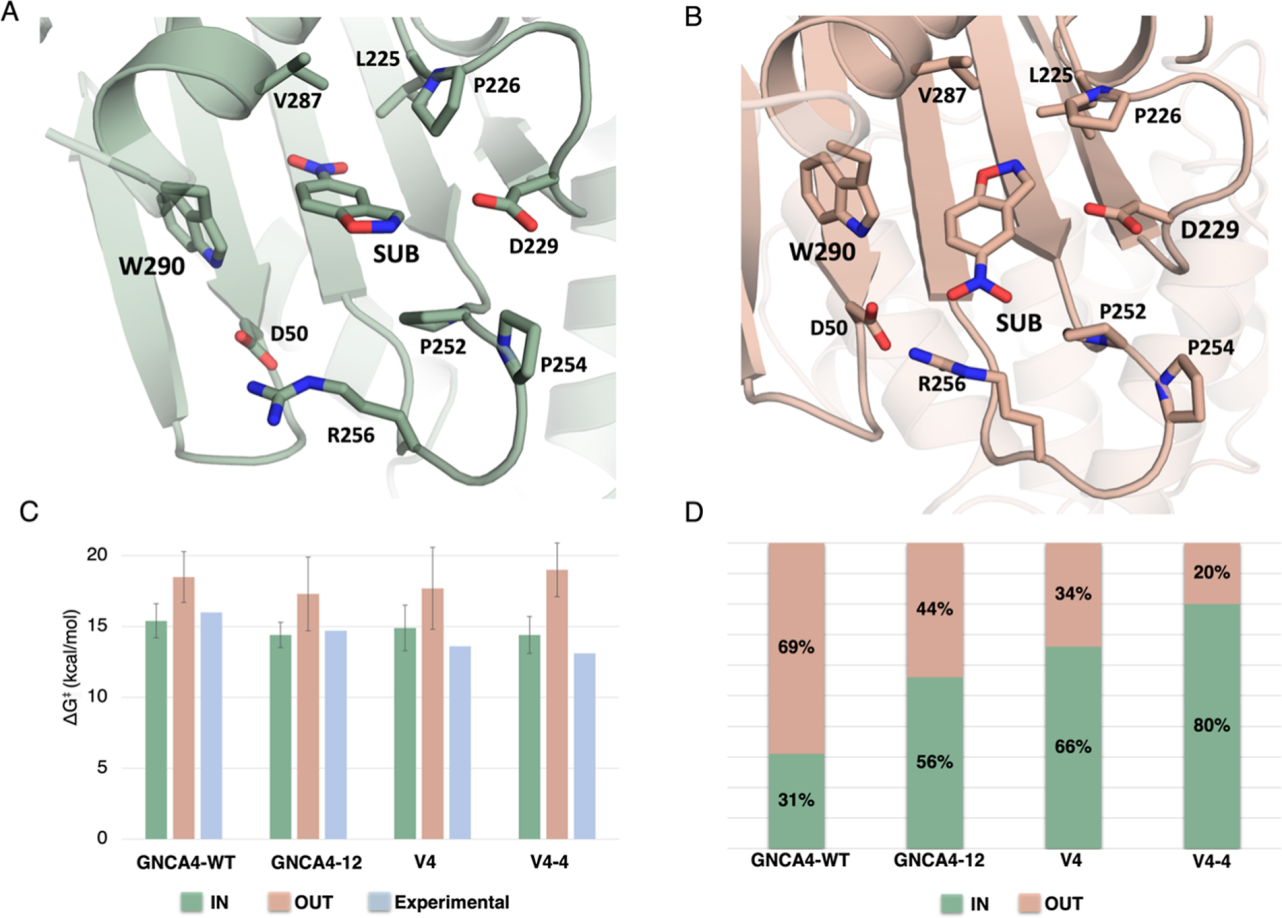
(A,B) Representative structures of different stable substrate conformations sampled in our simulations of the GNCA4-WT, with the substrate denoted as “SUB”. The “IN” conformation, in which the substrate is placed inside the active site pocket, is denoted in green, and the “OUT” conformation, in which the substrate points out of the active site pocket, is denoted in salmon. (C) Activation free energies (kcal mol^–1^) for the hydrolysis of 5-nitrobenzioxazole showing a comparison of experimental Δ*G* ^‡^ derived from the measured *k*_cat_ values (blue bar) and the corresponding calculated values obtained from EVB simulations initiated at either the “IN” substrate conformation (green bar) or the “OUT” substrate conformation (salmon bar). Error bars are presented as the standard deviations over 30 independent EVB trajectories of each system, with raw data presented in Table S10. (D) Percentage of simulation time the substrate spends in each of the “IN” and “OUT” conformations during our simulations of each variant, calculated as described in the Experimental Section.

The difference between these two substrate conformations is effectively a 180° substrate flip, and it is not implausible that both conformations are to some extent catalytic. To test this possibility, we performed empirical valence bond (EVB) simulations of each of the GNCA4-WT, GNCA4-12, V4 (GNCA4-12 with the polypeptide) and V4-4 variants, starting from crystal structures of each variant (modified from the GNCA4-12 structure in the case of the V4 and V4-4 variants), with the substrate initially placed in either of the “IN” and “OUT” conformations of the substrate (Figure 7C), allowing us to estimate which of the two substrate conformations are energetically preferred, as we are averaging over 30 independent trajectories (with conformational sampling across the trajectory) per system. The simulations initiated from the “IN” conformation give good agreement with experiment (see Figure 7 and Table S10), with a Pearson correlation coefficient of 0.69 between the calculated and experimental activation free energies (Figure S9). In contrast, the simulations initiated from the “OUT” conformation of the substrate consistently yield both higher activation free energies (by 3–5 kcal/mol) per variant, and poor correlation with experiment, again indicating that this is unlikely to be a catalytically competent substrate conformation. The IN conformation is further expected to be catalytically productive based on the positioning of the transition state analogues in the GNCA4-WT (PDB ID: 5FQK)^28^ and GNCA4-12 (PDB ID: 6TXD)^18^ active sites. In addition, it is not implausible (see the Supplementary Discussion) that the “IN” conformation promotes catalysis through a higher value of the p*K*_a_ of the catalytic group, a local-environment effect that could perhaps be more pronounced in the more active Kemp eliminases.

Finally, and interestingly, as shown in Figure 7D, similar to the HG3 series where evolution eliminates a nonproductive substrate binding conformation in the transition from the initial design to HG3.17,^32^ also here, our optimization trajectory from GNCA4-WT through to V4-4 (+ polypeptide segment) significantly reduces sampling of the “OUT” substrate conformation observed in GNCA4-WT. That is, while this conformation is dominant in the GNCA4-WT (although nearly 69/31 split), the proportion of the conformations equilibrates in GNCA4-12 (44/56). The situation is completely reverted for V4 and V4-4, for which we observe the “OUT” conformation in only 34% and 20%, respectively of our simulation time (vs 70% of simulation time in GNCA4-WT). Again, the reduction of the IN/OUT proportion observed from GNCA4-12 to V4 corroborates the enhancement of the catalytic activity when the polypeptide is added to the *C*-terminus of the enzyme. Furthermore, we observe strong correlations between the fraction IN/OUT and the experimental activation free energies (Figure S9), with a negative Pearson correlation coefficient of −0.99 between %IN and increasing activation free energy, and a positive Pearson correlation coefficient of 0.99 between %OUT and increasing activation free energy.

As shown in Figure S10, the polypeptide segment in V4-4 closes over the active site and is held in place by a hydrogen bonding interaction between D229 and the R266 from the polypeptide segment. On the one hand, this interaction, which is maintained for 44% of simulation time (at an average distance of 2.92 ± 0.24 Å taking snapshots every 10 ps of 5 × 1 μs trajectories) is catalytically detrimental as this salt bridge would be expected to stabilize the D229 side chain and thus decrease its p*K*_a_. However, this detrimental effect appears to be offset by the fact that this interaction makes the active site more compact, reducing its volume to 443.21.1 ± 165.7 Å^3^ in V4-4 compared to 700.9 ± 143.2 Å^3^ (for which the D229-R266 interaction is just kept for 2% of the simulation time), 651.2.9 ± 115.4 Å^3^ in GNCA4-12 and 1433.0.0 ± 132.8 Å^3^ in GNCA4-WT (as measured using Pocket Volume Measurer (POVME) 3.0, see the Experimental Section).

In summary, over the course of the designed trajectory, we observe two jumps in the IN/OUT ratio, with enrichment toward the catalytically preferred “IN” conformation. The first of these is between GNCA4-WT and GNCA4-12 (Figure 7). GNCA4-12 is the best variant from our initial FuncLib study.^18^ As shown in Figure 5 of ref 18 the structural changes between GNCA4-12 and the wild-type enzyme are minimal. However, our analysis of the conformational space of W290 (Figure 6) shows enrichment of W290 toward catalytically productive conformations. This in turn likely leads to the stabilization of the “IN” conformation in GNCA4-12, with this stabilization being further enhanced in V4-4 (which also includes the *C*-terminal polypeptide segment). This segment closes over the active site and restricts its volume contributing to stabilization of the “IN” conformation.

Taken together, our simulations show that conformational enrichment of catalytically productive substrate conformations and W290 side chain conformations is likely a major contributor to the enhanced activity of the evolved variants. This interplay between conformational selection and minimizing loss of electrostatic optimization in turn leads to the observed catalytic enhancement of the V4-4 variant, as well as, plausibly, the unusual pH dependence of this enzyme. We find this observation of interest, as such conformational enrichment of productive substrate poses has been observed in several natural and engineered enzymes. At a minimum, it has been observed in two other series of Kemp eliminases: the HG3 series^32^ mentioned above, and the KE07 series.^35^ We have further observed such enrichment in the transition of chalcone isomerases from a solute binding protein to enzymes,^46^ where the emergence of enzymatic activity on this scaffold is correlated with a 180° flip of the substrate from a nonproductive conformation in the ancestor to a productive conformation in the extant enzyme. Further, conformational selection of the active site (not substrate) has been observed during the evolution of a phosphotriesterase to an arylesterase in a phosphotriesterase from Pseudomonas diminuta.^47^ Substrate flipping has also been observed during the engineering of a selective halohydrin dehydrogenase, in this case impacting enzyme selectivity.^48^ Our data adds to this body of literature, suggesting it is not an obscure phenomenon.

### Using Interaction Networks to Predict Engineering Hotspots

Having established that the NMR determined hotspots can be optimized through computational stability design to obtain an exceptionally proficient Kemp eliminase, a logical question to ask is whether the process could have been performed fully computationally. That is given the expansion of approaches to predict allosteric communication and residue interaction networks computationally,^49–53^ would a computational approach be able to predict dynamically important hotspots that could be targeted for further engineering effort?

To test this, we have applied our recently developed tool, Key Interactions Finder (KIF),^54^ to explore how well KIF is able to predict experimentally determined NMR hotspots (Figure 8). KIF calculations were performed by comparing differences in interaction networks between the liganded and unliganded forms of the GNCA4-12 β-lactamase,^28^ that was used for determination of the NMR hotspots. As can be seen in Figure 8, several of the residues in the functionally important β-set (6/10) have KIF scores >0.1. Further, no residues in the α-set (which did not improve function when targeted), and only one residue in the active site set (which was already heavily optimized in our prior work^28^) yield large KIF residue importance scores.

**Figure 8:**
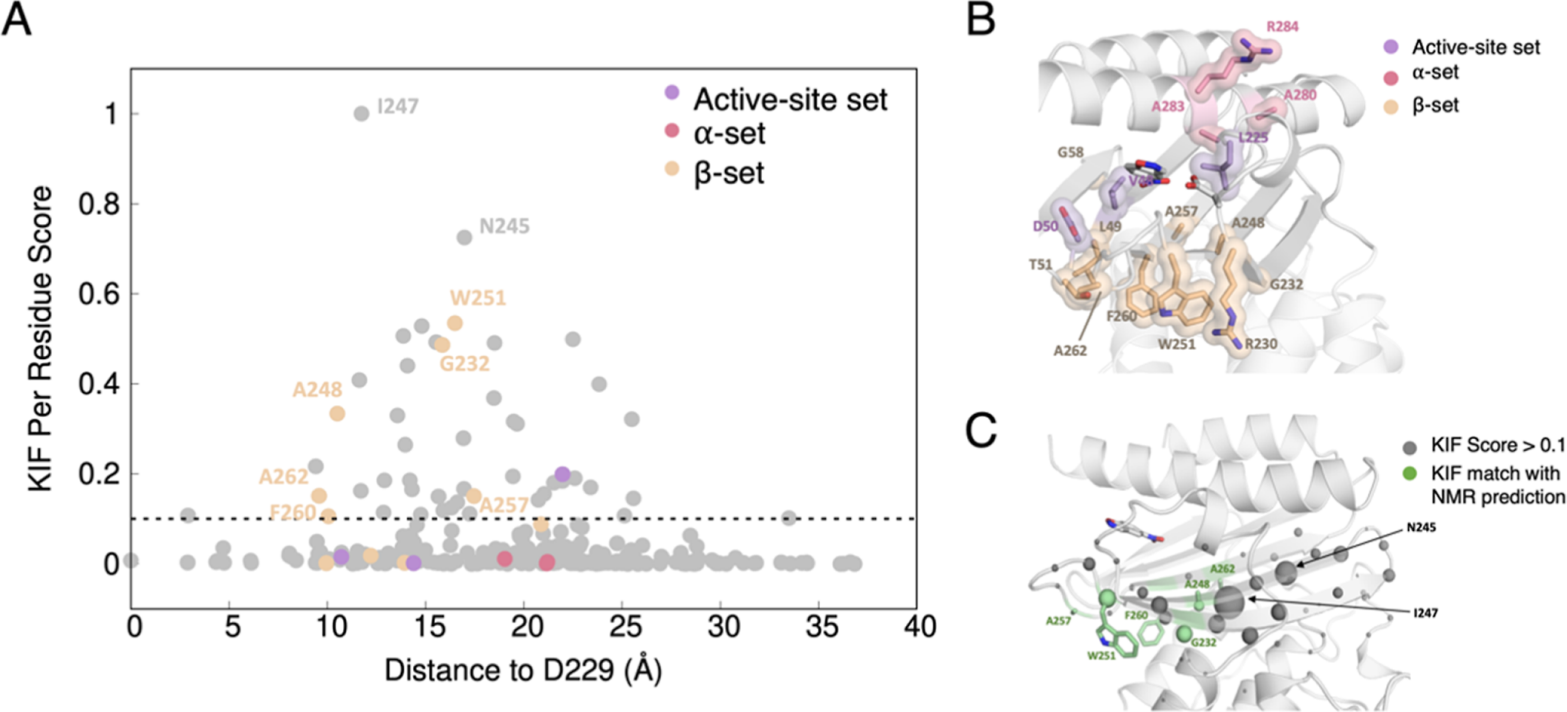
Prediction of residues involved in functionally important noncovalent interactions in GNCA4-12, using Key Interactions Finder (KIF).^54^ (A) Scatter plot of KIF-calculated residue importance scores, vs distance to the active site (using the C_α_-atom of D229 as a proxy). NMR-identified hotspots are colored based on the set they are included in. (B) Structure of GNCA4-12 (PDB ID: 6TXD),^18^ showcasing the different NMR-determined hotspot sets, colored using the same color scheme as in panel (A). (C) Projection of residues with KIF scores >0.1 onto the GNCA4-12 crystal structure (PDB ID: 6TXD),^18^ shown as gray spheres, with NMR-predicted hotspots with KIF scores >0.1 highlighted in green.

As Kemp elimination is an anthropogenic activity, for which we have engineered a de novo active site, we also wanted to test how well KIF would be able to predict hotspots in naturally occurring enzymes where key functionally important hotspots are known. To address this, we extended our KIF analysis to simulations of two additional systems: a chalcone isomerase ancestor inferred from ancestral sequence reconstruction^46^ (CHI ancCC, Figure S11) and a bacterial phosphotriesterase from P. diminuta^47^ (PTE, Figure S12).

Prior work on chalcone isomerase has shown that both substrate flipping and active site conformational dynamics are crucial for the evolution of CHI from a noncatalytic solute binding ancestor to extant proficient isomerases.^46^ Finally, laboratory evolution of the phosphotriesterase from P. diminuta (PTE) caused an activity switch to an arylesterase;^9,47,55,56^ structural and dynamical characterization of this trajectory highlighted the role of protein dynamics in allowing for the functional switch.^47^ Taken together, these three systems all show examples of cases where substrate or scaffold dynamics is important for function, and where hotspot residues controlling function have been identified through either laboratory evolution or NMR characterization. They thus provide excellent test cases for the ability of KIF to predict such functionally important positions.

As can be seen from the scatter plots shown in Figures 8, S11 and S12, encouragingly, KIF is able to predict a large number of hotspot residues in all systems (38% in GNCA4-12, 70% in CHI and 42% in PTE), with KIF importance scores of >0.1. This is particularly encouraging in the case of PTE, where we see an importance score of >0.8 assigned to His254 in the initial R0 variant of this enzyme (basically, wild-type PTE), given that the mutation of His254 to arginine was the first mutation along the PTE evolutionary trajectory to fixate in the evolution toward arylesterase activity.^47^ It is important to note that these are residues with high KIF scores that are known experimentally to be hotspots for directed evolution. There can be additional plausible hotspots among the residues flagged with high KIF scores that have simply not yet been tested experimentally (although not all these positions will be hotspots). However, KIF’s ability to predict ∼50% of experimentally known hotspots across these three systems is significant, both because one does not necessarily expect all hotspots to lie along residue communication networks, but also, because at a 50% hit rate, KIF is able to predict promising residues on which to focus directed evolution efforts, which is particularly valuable in systems where complete determination of NMR hotspots is not possible (due to system size, as one example).

We note here that KIF by design predicts important residues and interactions across the entire protein scaffold, and not just at known mutational hotspots. While any of these are potentially targets for design effort, it would be helpful to have a means to filter hotspots to subject to further optimization and not have to test the full KIF-predicted set. In this context, in prior work, we computed detailed residue interaction networks to calculate evolutionary conservation of such interactions in Class A β-lactamases.^57,58^ To test the efficacy of KIN as a tool for filtering hotspots, we have compared our KIF calculated hotspots against evolutionarily conserved positions predicted using KIN (Figure 9). This analysis provides two important observations: (1) with two exceptions, interactions involving the ten hotspot residues that are part of the NMR-determined β-set also have low KIN evolutionary conservation scores. Specifically, G232 and W251 have the highest KIN conservation scores from this set, L49 and T51 have non-negligible but much lower KIN conservation scores, and the remaining six β-set residues all have KIN conservation scores of 0.05 or less, and are thus not annotated on the figure. This indicates that the β-set residues, in particular those with negligible KIN conservation scores, would be good targets for design, as evidenced by our successful optimization of these positions. Further, (2) a number of NMR-determined α-set residues have non-negligible KIN scores, indicating that these positions are less likely to be amenable to successful optimization, again consistent with our unsuccessful attempts to improve activity when targeting these positions. Finally, it is worth noting that, in the case of the β-set residues with higher KIN conservation scores, specifically G232 and W251, FuncLib also proposed relatively limited sequence space (Table S6). Further, in the top scoring FuncLib variants for the β-set (Supporting Information Table 2), G232 remains largely unchanged, and W251 is modified to other aromatic residues.

**Figure 9:**
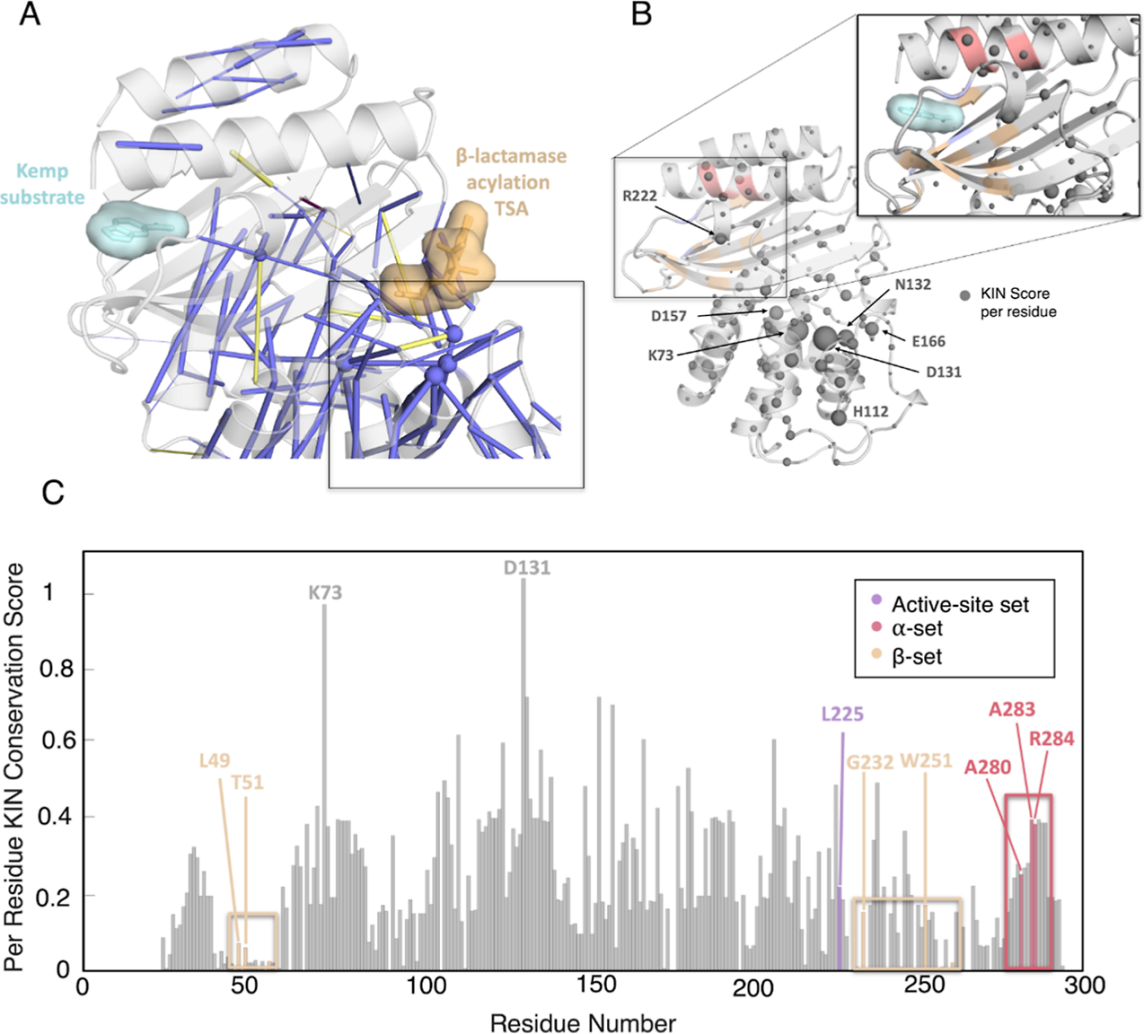
Predicting the evolutionary conservation of noncovalent interactions in Class A β-lactamases, using key interactions network (KIN).^57,58^ (A) Visualization of evolutionarily conserved interactions in the TEM-1 β-lactamase (PDB ID: 1M40),^90^ which was used as a reference for the KIN analysis, using a 50% simulation retention cutoff for the analysis. A transition state for the β-lactamase reaction present in the TEM-1 crystal structure used (pinacol­[[2-amino]-α-(1-carboxy-1-methylethoxyimino)-4-thiazoleacetyl]­amino]­methaneborate) is shown in orange, and the substrate for Kemp elimination (5-nitrobenzisoxazole, placed manually in the de novo active site location for illustration) is shown in cyan, to indicate the position of the two active sites relative to each other. Blue lines denote hydrogen bonds, and yellow lines donate salt bridges. The relative conservation of the interaction is indicated by cylinder size. (B) Projection of per-residue KIN evolutionary conservation scores, with each residue’s C_α_-atom shown as spheres (relative conservation indicated by sphere size). NMR identified hotspots are colored based on the set they are included in, using the same coloring as in Figure 8. (C) Per residue KIN scores, with NMR identified hotspot residues highlighted and colored based on the set they are included in. Note that only residues with non-negligible KIN scores are labeled.

These two observations are particularly interesting, as the interaction networks calculated by KIN are based on evolutionary information across Class A β-lactamases, which have been evolutionary optimized to support the antibiotic degradation activity of this scaffold, and not the de novo Kemp eliminase activity. Despite this, KIN provides an effective tool at filtering hotspots, and we posit that positions that have both high KIF scores and low KIN conservation scores will be effective targets for optimization. Taken together, we believe this would be a viable pathway to perform hotspot prediction and optimization fully computationally prior to experimental testing, leading to highly efficient enzymes. On this premise, we suggest a simplified computational workflow to use KIF to guide experimental or computational protein optimization: (1) Use KIF to identify key interaction networks and/or residues with importance scores above a user-defined threshold. (2) Apply filtering, for instance through multiple sequence alignment, or tools like our partner tool key interaction networks (KIN),^57^ to identify evolutionary conserved positions or interactions. Modifications at these positions will likely be highly detrimental to activity. (3) Once a manageable number of hotspots have been filtered, these can be subject to randomization using computational tools such as FuncLib,^21^ to predict promising variants, which can finally (4) be subjected to further experimental design and/or testing.

Finally, we emphasize that the computational approaches described in this work are not, of course, claimed to be more efficient than the experimental NMR approach at determining catalytic hotspots. However, they should provide an immediately feasible approach when, due to problems such as the size of the protein, limitations on the available protein or simply the lack of a suitable transition-state analogue, the NMR analysis is not possible, or it is high demanding in terms of the experimental effort required.

## Concluding Remarks

Enzyme optimization for biotechnological applications is challenging. Standard directed evolution is typically sluggish and time-consuming.^10^ Furthermore, simultaneous enhancement of several crucial biomolecular features is rarely achieved in enzyme engineering.^8^ These two common experimental scenarios obviously reflect that the protein sequence space is vast and that most sequences do not encode for proteins with the desired properties.

Here, we have combined two recently proposed strategies of guided directed evolution to enhance a previously engineered de novo enzyme capable of catalyzing a non-natural reaction. The two strategies are fundamentally different and target different protein features. Yet they complement each other synergistically and lead to a laboratory evolution protocol that is highly efficient in terms of both, the very limited amount of screening required, and the pattern of multiproperty optimization achieved.

Our two-pronged approach (computational design of stabilizing variants targeting NMR-determined catalytic hotspots) sharply focuses screening to the small regions of the sequence space in which catalysis is efficiently modulated without compromising stability. In this way, we avoid the detrimental catalysis-stability trade-offs that are often observed^8^ as a result of the fact that most mutations in a protein are destabilizing.^7^ The effectiveness of our approach is highlighted by the fact that we targeted for enhancement a de novo Kemp eliminase which had already been highly optimized by previous engineering efforts.^18,20,28^ Based on the already high activities of our starting enzyme (*k*_cat_ ∼ 600 s^–1^ and *k*_cat_ */K*_M_ ∼ 2 × 10^5^ M^–1^ s^–1^) one would expect further rate enhancements to be challenging if not even impossible. Particularly, our starting variant already has activities comparable to HG3.17,^32^ the best proton abstraction Kemp eliminase to date, *k*_cat_ = 700 s^–1^, *k*_cat_ */K*_M_ = 2.3 × 10^5^ M^–1^ s^–1^, which resulted from 17 rounds of directed evolution on an iterative design background. Yet, despite this high starting activity, we were able to achieve additional optimization and reach de novo biocatalysis levels that are above most reported values for engineered de novo enzymes and modern natural enzymes^4,34^ while also improving stability, as shown by increased denaturation temperature. The only current Kemp eliminase with higher activity than ours is redox-mediated and exploits metal ion catalysis;^19^ our best variant in this work achieves *k*_cat_ ∼ 1700 s^–1^, *k*_cat_/*K*_M_ ∼ 4.3 × 10^5^ M^–1^ s^–1^ through classical proton abstraction without the need for metal ions.

Molecular dynamics simulations and empirical valence bond (EVB) calculations on the best Kemp eliminase obtained in this work, together with some of its precursors, provided insight into the molecular mechanisms behind the enhancement achieved. Improved de novo activity is not linked to modifications in the catalytic machinery but, rather, to shifts in the conformational ensembles of both, crucial active-side residues and bound substrate, in such a way that productive conformations are promoted, while the population of nonproductive conformations is decreased. Such conformational diversity at the Michaelis complex would also serve to rationalize the unusual pH rate profiles (*k*_cat_ vs pH) shown in Figure 5, with slopes of log_10_ *k*_cat_ vs pH that are below unity. Different metastable substrate conformations would be expected to experience different local environments for the aspartic acid, which in turn could impact the p*K*_a_ values. We also note that a similar conformational selection mechanism has been described for the Kemp eliminases HG3 and KE07.^32,35^ It must be noted, however, that, unlike HG3 and KE07, the active site of our Kemp eliminase was originally generated^28^ using a single-mutation minimalist design that may mimic the actual emergence of new enzymes during natural evolution. Our results therefore suggest the relevance of the conformational selection in the evolutionary optimization of primordial enzymes.

It is also of interest that conformational selection in our Kemp eliminase enzyme is linked to the roughly additive effects of interactions generated by an extra polypeptide chain^20^ and by mutations predicted by the FuncLib server^21^ at catalytic hotspots determined through NMR chemical shift perturbation. Remarkably, the residues at such catalytic hotspot positions do not bear side-chains at the active site, thus supporting the potential of the NMR approach^19^ to guide directed evolution by identifying useful positions linked to dynamic effects that would not be obvious in the enzyme 3D-structure. We note that this NMR-guided approach has been successfully coupled with saturation mutagenesis to repurpose a natural enzyme, myoglobin, as a redox-mediated Kemp eliminase.^19^ Here, we extend this approach to a de novo active site, replacing saturation mutagenesis with FuncLib analysis.^21^ We further demonstrate that, in cases where NMR analysis is not practical or accessible, hotspots can be effectively predicted using key interactions finder (KIF)^54^ to identify residues involved in functionally important noncovalent interactions, and then filtering high scoring residues using key interaction networks (KIN)^57^ to eliminate residues that are involved in highly evolutionarily conserved interactions as hotspots.

Curiously, our KIN analysis is based on Class A β-lactamases more broadly, i.e. the interaction network was evolutionarily optimized for the natural not de novo activity, and yet we show (Figure 9) that the KIN scores nevertheless successfully discriminates between catalytically productive and nonproductive sets of hotspots predicted from the NMR (residues from the β-set, which was successfully targeted for optimization, have low KIN evolutionary conservation scores, and conversely, residues from the poorly performing α-set have non-negligible KIN scores). Similarly, FuncLib predicts mutations based on evolutionary information (phylogenetic analysis), and therefore would be expected to work best on naturally occurring enzymes. Yet both here, and in our prior work,^18^ we demonstrate that FuncLib is also a powerful tool for engineering de novo active sites. In both cases, it appears that information from natural evolution can be transferred also for optimizing a de novo activity.

More generally, the fact that a large modulation of catalysis is achieved through substrate conformational selection suggests that the engineering approach proposed in this work could be used to stabilize a preferred conformation of the bound substrate that favors a specific outcome of the chemical transformation. This suggests the potential of our approach for engineering of systems where substrate binding pose is important, including, plausibly, enzyme regio- and stereoselectivity.

Overall, we show here an enhancement in the biomolecular features of an anthropogenic enzyme, from a starting point that had already been heavily optimized through both laboratory evolution (using ancestral protein as scaffolds) and protein engineering. Taken together, our results both provide insight into the molecular mechanisms of enzyme evolution and demonstrate a highly efficient experimental/computational approach to catalysis/stability enhancement that will contribute to expand the scope of biotechnological applications of enzymes through targeted engineering of protein conformational dynamics.

## Experimental Section

### Protein Expression and Purification

The various enzymes studied here were prepared as described in our previous work.^18,20,28^ Briefly, genes were cloned into a pET24-b with resistance to kanamycin and subsequently transformed into E. coli BL21 (DE3). The proteins were purified by affinity chromatography taken advantage of a His-tag attached at the *C*-terminus. Protein stock solutions were prepared by exhaustive dialysis. Protein concentrations were determined spectrophotometrically using known values of the extinction coefficient.

### Determination of Rates of Kemp Elimination

The kinetic of enzyme-catalyzed Kemp elimination was studied as we have previously described in detail.^18,20,28^ Briefly, Kemp elimination was followed by using absorbance at 380 nm to observe product formation. Rates were calculated from the initial slopes of the dependence absorbance with time using an extinction coefficient of 15,800 M^–1^ cm^–1^. All activity values were corrected for a blank determined under the same conditions. In all cases, the corrections were small or negligible. Kinetic experiments were carried out within the pH range 6–8.5 at 25 °C in HEPES buffer 10 mM with NaCl 100 mM. At each pH value, kinetic experiments with different substrate concentrations were carried out. Stock solutions of the Kemp substrate were prepared in acetonitrile. Therefore, variable amounts of acetonitrile were added to experiments with different substrate concentrations in order to ensure that the final acetonitrile concentration in the reaction mixture was 1% in all cases. Profiles of rate vs substrate concentration were fitted with the Michaelis–Menten equation in order to determine the values of the catalytic efficiency (*k*_cat_ */K*_M_), the catalytic rate constant or turnover number (*k*_cat_) and the Michaelis constant (*K*_M_). Determination of *k*_cat_ relies on the experimental observation of curvature in the plots of rate vs substrate concentration. Detection of such curvature is often difficult in studies of Kemp eliminases because low solubility limits the experimentally available substrate concentration range. We note, however, that the reliability of the curvature in the plot of rate vs substrate concentration for the best Kemp eliminase reported in this work is clearly supported by the excellent agreement between the results of experiments independently performed with two different protein preparations (Figure 3B).

### Determination of Protein Stability

Protein thermal stability was assessed by differential scanning calorimetry following protocols that we have previously described in detail.^42^ Experiments were performed with protein solutions in HEPES 10 mM NaCl 100 mM pH 8.5. In all cases, a single calorimetric transition was observed. The denaturation temperature, defined as the temperature of the maximum of the calorimetric transition was used as a metric of protein stability.

### Preparation and Screening of Saturation Libraries

Saturation libraries at the positions in the active-site set were prepared using the QuickChange Lighting PCR method (Agilent #210518). The codon corresponding to each position was replaced by NNN. Library preparation, transformation and quality control of the library were carried as we have previously described in detail.^20^ Library screening was also performed following exactly a protocol that we recently described^20^ and that took advantage of the availability of a Freedom EVO 200 robot from TECAN (Männedorf, Schweiz), except for the buffer and the Kemp substrate concentration employed: HEPES 10 mM NaCl 100 mM pH 8.5 and 0.06 mM, respectively. About 90 colonies were screened for each library to thus ensure a high probability that all of the 20 variants were screened at least once. Results are shown in Figure S4.

### NMR Experiments

All NMR experiments were performed at 31.5 °C on a Bruker AV NEO 800 spectrometer equipped with a cryoprobe on a 0.4 mM uniformly ^13^C, ^15^N-labeled sample. Sequence-specific assignments were made using the standard procedures on the basis of the following experiments: 2D ^1^H, ^15^N HSQC and 3D HNCO and HNCA using a BEST TROSY versions.^59^ NMRPipe^60^ and NMR View^61^ were used to process the raw NMR data and to perform interactive spectrum analysis, respectively. Chemical shifts were referenced to the water signal as an internal reference for ^1^H using pH and temperature corrections.^62,63^ ^15^N and ^13^C chemical shifts were referenced indirectly.^64^

The titration experiments were performed by stepwise addition of a concentrated solution of the transition-state analogue to a ^13^C, ^15^N GNCA4-12 protein sample. After each addition, changes in chemical shifts of the protein resonances were monitored in 2D BEST TROSY ^1^H, ^15^N HSQC spectra. A total of six protein/transition-state-analogue ratios were examined: 1:0.1, 1:0.14, 1:0.2, 1:0.4, 1:1.1 and 1:2.1. The average amide CSP (Δδ_avg_) were obtained at 2-fold molar excess of transition-state analogue as Δδ_avg_ = [((Δδ_N_x0.4)^2^ + Δδ_H_^2^)/2]^1/2^, where Δδ_N_ and Δδ_H_ are the CSP of the amide nitrogen and the proton, respectively. For each observed resonance, the CSP *Z*-score^19^ was calculated as *Z* = (Δδ_avg_ – μ)/σ, where μ and σ are, respectively, the average and the standard deviation of Δδ_avg_ values for a given experiment.

### FuncLib Screening of Mutational Hotspots

Mutational hotspots were screened using the FuncLib Web server (https://funclib.weizmann.ac.il), as described in ref 21. Screening was performed on each of the α- and β-sets alone (positions 280, 284 and 284, and positions 49, 51, 58, 230, 232, 248, 251, 257, and 262, respectively) as well as a combination of both the α- and β-sets from the NMR chemical shift experiments. Note that the full β-set contains 10 residues; however, F260 from the β-set was kept fixed in these screens, as prior work highlighted the importance of the L260F mutation in enhancing the Kemp eliminase activity.^18^ All calculations we performed on chain A of the GNCA4-12 variant, PDB ID: 6TXD,^18^ for consistency with the NMR chemical shift experiments. D229 and W260 were identified as essential amino acids, and the His tag was retained in the FuncLib predictions. The multiple sequence alignment was performed using the default FuncLib parameters, with both runs generating ∼1000 predicted variants. The top 20 ranked designs, based on their stability score, were retained for further experimental characterization. The sequence space randomized and their predicted stability by FuncLib is presented in Tables S3, S6 and Supporting Information Tables 1 and 2.

### Simulation Setup and Structure Preparation

Four different systems were simulated using molecular dynamics (MD) and empirical valence bond (EVB) simulations^65^ in this work: the GNCA4-WT, GNCA4-12, and the V4-4 variant, the latter being simulated both with and without the additional polypeptide segment.^20^ All simulations were performed in complex with the substrate 5-nitrobenzisoxazole, which was manually modeled into all systems based on the position of the transition state analogue (TSA) 6-nitrobenzotriazole in the GNCA4-WT structure (PDB ID: 5FQK).^28^ In the case of GNCA4-WT and GNCA4-12, available crystallographic structures were used as starting points for the simulation (PDB IDs: 5FQK and 6TXD, respectively.^18,28^ The polypeptide segment of V4 was modeled into the GNCA4-12 construct from sequence using Modeler.^66^ Finally, the V4-4 starting structure was prepared by first manually inserting the corresponding mutations into the V4 construct, followed by structure optimization using AlphaFold.^67^ In all cases, the substrate 5-nitrobenzioxazole was manually placed in the active site in the position of the transition-state analogue 5(6)-nitrobenzotriazole present in the crystal structure. Additionally, His tags were retained in our simulations for better direct comparison with experiment. All starting structures employed during this work, and any modifications made to them during setup, are summarized in Table S11. Descriptions of system set up for additional simulations provided in this work for KIF^54^ analysis are provided as Supporting Information.

Finally, the p*K*_a_s of ionizable residues in all systems were predicted using PROPKA 3.0 ^68,69^ to identify any amino acid side chains with potentially anomalous p*K*_a_s. Based on this, in addition to the expected elevated p*K*_a_ of the catalytic base D229, D246 and K234 were both predicted to have anomalous p*K*_a_s (in the range of 8.7–11.0 for D246 and 6.3–6.5 for K234), which was confirmed to be reasonable by visual inspection of the local environment of these side chains, and thus these side chains were kept in their neutral states throughout our simulations. All other side chains were kept in their standard protonation states at physiological pH. All systems were solvated in a truncated octahedral water box of OPC water molecules,^70^ extending 11.0 Å from the protein in all directions, and all systems were neutralized using Na^+^ counterions (8 for GNCA4-WT, 9 for GNCA4-12, and 10 for V4-4 with and without the polypeptide segment). Finally, all hydrogen atoms in the system were scaled using hydrogen mass repartitioning.^71^

### Molecular Dynamics Simulations

All MD simulations in this work were performed using the CUDA-accelerated version of Amber22,^72^ using the ff19SB force field,^73^ and the OPC water model.^70^ Partial charges for the substrate were calculated at the HF/6-31G­(d) level of theory by restrained electrostatic potential (RESP)^74^ fitting using Antechamber,^75^ based on gas-phase geometries optimized at the B3LYP/6-31G­(d) level of theory, using Gaussian 16 Rev. B.01.^76^ All other force field parameters used to describe the substrate 5-nitrobenzisoxazole were obtained using the General AMBER Force Field (GAFF2),^77^ and all nonstandard substrate parameters are provided for reference in Table S12, and in the Zenodo data package submitted at DOI: 10.5281/zenodo.12666978.

MD simulations of all systems were performed using the same protocol: each replica for each system was first energy minimized using the steepest descent algorithm for 100 steps, followed by 900 steps of conjugate gradient minimization, with a 100 kcal mol^–1^ Å^–2^ restraint applied to all solute (protein and substrate) atoms. The complete system was then heated from 50 to 300 K in an *NVT* ensemble, using a simulated annealing protocol, in which the system was able to reach 300 K in the first 100 ps of heating. This was continued for a total of 1 ns, using a 1 fs time step, and Langevin temperature control, with a collision frequency of 1 ps^–1^. The 100 kcal mol^–1^ Å^–2^ restraint applied to all solute atoms was retained until this point in the equilibration, and then reduced from 100 to 10 kcal mol^–1^ Å^–2^ in subsequent equilibration steps. Following this heating, a second energy minimization and heating were performed using the prior protocol, with positional restraints applied to just the solute heavy atoms. During the subsequent equilibration steps, the restraints were progressively reduced from 10 to 1 to 0.1 kcal mol^–1^ Å^–2^, until the heavy atom restraints were removed in the final step. The final systems with only this distance restraint applied were then equilibrated for a final 1 ns in an *NPT* ensemble (300 K, 1 atm), using a Berendsen barostat with a 1 ps pressure relaxation time, and Langevin temperature control with a collision frequency of 1 ps^–1^. The NPT simulations were again performed using a 1 fs simulation time step, and for all simulations the SHAKE algorithm^78^ was applied to restrain all bonds to hydrogen atoms.

Finally, production MD runs were performed using a 4 fs time step, facilitated by hydrogen mass repartitioning^71^ and the SHAKE algorithm^78^ to constrain all bonds containing hydrogen atoms, an 8 Å direct space nonbonded cutoff, Langevin temperature control (collision frequency of 1 ps^–1^), and a Berendsen barostat (pressure relaxation time of 1 ps). Note that in addition to the heavy atom restraints described above, for the duration of our equilibration and production simulations, a further 5 kcal mol^–1^ Å^–2^ wall restraint was placed on the donor carbon of the substrate and the acceptor oxygen atom of the D229 side chain that kicks when the two atoms are 3.0 Å or greater apart, in order to prevent substrate dissociation from the active site during our molecular dynamics simulations. This application of this restraint also takes into account prior experimental studies of enzymatic Kemp elimination (see the Brønsted plot in Supplementary Figure 3 of ref 32 which suggests substantive proton transfer to the catalytic base at the transition state for Kemp elimination). Equilibration of these trajectories is shown in Figure S13.

The final production trajectories for all systems were 1 μs in length each, and each system was simulated in 5 different replicas, leading to a cumulative 5 μs of simulation time per system and 20 μs of simulation time across all systems.

### Empirical Valence Bond Simulations

Following our prior work,^18,28^ empirical valence bond simulations were performed on the GNCA4-WT, GNCA4-12, V4 and V4-4 variants, using the protocol and parameters presented in ref 18 Protonation states of ionizable residues within the explicit simulation sphere, as well as histidine protonation patterns (both of which were validated by PROPKA 3.0 ^70,71^ and visual inspection), can be found in Table S13. Note that we subtly updated our Morse and van der Waals parameters for the bonds changing during the Kemp elimination reaction in order to minimize system instabilities upon reaching the product state (otherwise in some replicas, too much force builds up on the proton being transferred leading to a system explosion). The initial structures for the “IN” and “OUT” substrate conformations was prepared either using the position of the transition state analogue in the original crystal structure of each variant as a reference (“IN”) or by manually modifying the orientation of the substrate and keeping a reactive donor–acceptor distance and angle to mimic the conformation observed in MD simulations (“OUT”). Sample input files, updated parameter files and starting structures have all been submitted to Zenodo, DOI: 10.5281/zenodo.12666978, along with the corresponding files describing our molecular dynamics simulations. In brief, each system was simulated in 30 replicas of 30 ns equilibration each followed by 10 ns of EVB simulations (200 ps window over 51 discrete EVB windows), leading to a cumulative 600 ns of EVB simulation time per system (including crystal “IN” and “OUT” EVB calculations), and 2.4 μs of EVB simulation time across all systems studied in this work. All EVB simulations were performed using the Q6 simulation package,^79^ the OPLS-AA force field,^80^ the TIP3P water model,^81^ and the surface constrained all atom solvent (SCAAS) model^82^ to describe solvent. Long range interactions were described using the local reaction field (LRF) approach.^83^ For further simulation details, see our prior work.^18,28^

### Simulation Analysis

Unless specified otherwise in the text, all MD analysis was performed using the CPPTRAJ module^84^ of AmberTools23.^85^ All analysis is based on extracting frames every 10 ps of each simulation trajectory and is presented (where relevant) as averages and standard deviations over 5 × 1 μs trajectories per system. In the case of donor–acceptor distance and donor-hydrogen-acceptor analysis, the “closest” keyword was used to ensure that distances are always being measured to the closest oxygen atom to the donor, due to the possibility of side chain rotation. The percentages of different IN/OUT substrate orientations in the active site were performed by counting frames, with the substrate orientation being defined by the angle by the C_α_ atom of residue D229, the C and N atoms of the substrate, and the C_α_-atom of residue V287. An “IN” conformation was defined as a conformation with a dihedral angle in the range of 51–180°, and an “OUT” conformation was defined as a conformation with a dihedral angle in the range of −180–50°. Similarly, the percentage of the hydrogen bond formed by D229 and the arginine of the polypeptide segment in the V4-4 variant with polypeptide segment^20^ and its average value was calculated by counting frames defined by the donor (O atom of D229) and acceptor (N atom of the arginine of the polypeptide segment), with a distance cutoff of 3.5 Å. Active site volumes for the different variants were calculated using Pocket Volume Measurer (POVME) 3.0,^86^ with snapshots taken every 100 ps of the simulations. Finally, PyMOL was used for visualization analysis.^87^

### Interaction Network Analysis

Key interactions finder (KIF) calculations^54^ were performed using mutual information analysis^88,89^ to calculate the per residue interaction scores. These were obtained by comparing noncovalent interactions in the presence and absence of ligand in each system considered. Analysis was performed over 25,000 structures per system (extracted every 0.02 ns of our MD simulations). All hydrogen bonds, salt bridges and hydrophobic interactions in each system were considered for the analysis, and interactions with an occupancy of <50% of simulation time were excluded.

The evolutionary conservation of the noncovalent interactions in the Class A β-lactamase family was analyzed using key interaction networks (KIN).^57^ This analysis built on prior work considering the evolution of interaction networks in these enzymes,^58^ now including also the GNCA4-12 variant in the analysis. As in our prior work,^57,58^ TEM-1 was selected the reference structure for projection of the network, owing both to its evolutionary connection to the chosen ancestral sequences, and its chemical significance as a catalytic specialist. The KIN calculated conservation network provides a relative conservation score for each interacting residue pair, based on how frequently each interaction appears across the structures. A cutoff of 50% conservation was used in interaction analysis. From this, per residue KIN evolutionary conservation scores were calculated for direct comparison to the KIF analysis.

## Supplementary Material



## Notes

Supplementary tables, supplementary figures including computational details and additional experimental data, and supplementary discussion on the pH-dependence of the Michaelis–Menten parameters. Supplementary Data Table 1 and Supplementary Data Table 2 provide the full FuncLib prediction set (PDF)

## ○

School of Chemistry and Max Planck-Bristol Centre for Minimal Biology, University of Bristol, Cantock’s Close, Bristol BS8 1TS, U·K
