# Supplementary material for: Enzyme Enhancement Through Computational Stability Design Targeting NMR-Determined Catalytic Hotspots
Source: J Am Chem Soc. 2025 Mar 19;147(18):14978–96. doi: 10.1021/jacs.4c09428 (PMC12151457; doi:10.1021/jacs.4c09428)
Supplement: Supplementary file 1 [file ja4c09428_si_001.pdf]

Supporting Information for:

Enzyme enhancement through computational stability design targeting NMR-determined catalytic hotspots

Luis I. Gutierrez-Rus,[⊥1] Eva Vos,[⊥2] David Pantoja-Uceda,[⊥3] Gyula Hoffka,[4-6] Jose Gutierrez-Cardenas,[2,7] Mariano Ortega-Muñoz,[8] Valeria A. Risso,[1] Maria Angeles Jimenez,[3]* Shina C. L. Kamerlin,[2,6]* Jose M. Sanchez-Ruiz[1]*

[1] Departamento de Química Física, Facultad de Ciencias, Unidad de Excelencia de Química Aplicada a Biomedicina y Medioambiente (UEQ), Universidad de Granada, Granada, Spain.

[2] School of Chemistry and Biochemistry, Georgia Institute of Technology, Atlanta, Georgia, USA.

[3] Departamento de Química Física Biológica, Instituto de Química Física Blas Cabrera (IQF-CSIC), Madrid, Spain.

[4] Department of Biochemistry and Molecular Biology, Faculty of Medicine, University of Debrecen, Hungary.

[5] Doctoral School of Molecular Cell and Immune Biology, University of Debrecen, Hungary.

[6] Department of Chemistry, Lund University, Lund, Sweden

[7] Department of Chemistry and Biochemistry, Kennesaw State University, Kennesaw, Georgia, USA.

[8] Departamento de Química Orgánica, Facultad de Ciencias, Unidad de Excelencia de Química Aplicada a Biomedicina y Medioambiente (UEQ), Universidad de Granada, Granada, Spain.

* Corresponding authors:
majimenez@iqf.csic.es, skamerlin3@gatech.edu, sanchezr@ugr.es

⊥ L.I.G.R., E.V. and DPU contributed equally to this work.

Table of Contents

S1. Supplementary Tables……………………………………………..S3

S2. Supplementary Figures…………………………………...…....S26

S3. Supplementary Discussion…………………………………...…..S39

S3-1. pH-dependence of the Michaelis-Menten parameters for Kemp eliminases based on the proton-abstraction mechanism…….…………….S39

S3-2. The Pareto front…………………...…………………………….S44

S4. Supplementary Computational Methods ………………………...S45

S5. Supplementary References……………………………………….S47

S6. Supplementary Data Table 1……………………………………..S50

S7. Supplementary Data Table 2……………………………………..…S51

S1. Supplementary Tables

Table S1. Description of Kemp eliminases studied in this and previous works.

**Variants**	**Modifications**	**Mutations**	**PDB ID**	**Catalytic Efficiency (k_{cat}/K_M, M^{-1} s^{-1})[a]**
$GNCA_{MP}$[1]	Initial ancestral β-lactamase scaffold	-	4B88	-
$GNCA_{MP}$-W229D[2]	Minimalist active site by single mutation of $GNCA_{MP}$	W229D	4UHU	45±6
GNCA4-W229D[2]	Best scaffold variant for initial Kemp eliminase catalysis	W229D	--	250±7
GNCA4-W229D/F290W[2]	Second mutation in the minimalist active site	F290W	5FQI	3047 ± 283
GNCA4-12[3]	FuncLib mutations on GNCA4-W229D/F290W	I250V<br>R256H<br>L260F<br>V261L<br>H291V	6TXD	4127 ± 460
V4[4]	Addition of polypeptide segment over GNCA4-12	-	--	$(2.0±0.1)·10^5$
V4-4	Mutations in V4	L49I<br>G58Q	--	$(4.3±0.1)·10^5$

		R230I<br>A257P<br>A262C		

[a] Catalytic efficiency reported for Kemp elimination reaction.

Table S2. List of ^1H_N, ^{15}N, $^{13}C'$, and ^{13}Ca chemical shifts (ppm) of ^{15}N,^{13}C-GNCA4-12 derived from analysis of 3D HNCO and HNCA spectra.

Number	Residue	^1H_N	^{15}N	$^{13}C'$	$^{13}C_a$
30	S	8.651	112.762	176.12	63.28
31	E	7.228	121.539	179.54	59.15
32	Q	8.061	120.121	180.43	58.87
33	L	8.402	120.906	----	57.18
35	E	7.398	119.093	178.71	59.26
36	L	7.522	123.162	178.50	57.53
37	E	8.835	120.153	180.04	59.48
38	K	7.928	121.815	179.79	59.89
39	R	7.773	120.201	178.46	59.21
40	S	8.101	114.476	175.81	60.69
41	G	7.663	111.812	172.42	46.17
42	G	7.881	107.551	171.61	44.70
43	R	9.116	124.647	172.73	55.08
44	L	10.223	131.676	172.85	53.34
45	G	9.493	112.900	172.63	44.47
46	V	9.457	122.967	174.44	60.39
47	A	8.866	126.961	175.47	51.16
48	V	8.743	119.668	175.52	59.77
49	L	8.862	129.338	174.59	54.61
50	D	8.813	125.966	177.15	53.06
51	T	7.945	115.711	175.87	64.32
52	A	8.664	124.804	179.05	54.83
53	T	6.712	101.821	176.13	60.71
54	G	8.211	112.386	----	45.57
58	G	7.363	115.764	170.61	44.41
60	Y	8.940	124.428	173.80	58.83
61	R	8.626	123.321	177.47	58.20
62	G	7.987	136.143	173.26	47.06
63	D	8.026	113.588	175.85	52.92
64	E	7.217	120.861	174.73	55.73
65	R	8.013	116.752	----	55.90
68	M	8.482	123.250	----	60.13

71	T	7.691	113.378	----	65.72
73	K	7.465	122.574	177.26	59.49
74	A	6.576	117.062	176.58	55.17
75	L	6.616	114.127	----	56.26
77	A	7.443	117.931	178.24	55.10
78	A	7.089	117.716	176.97	55.27
79	A	7.687	119.896	180.21	54.31
80	V	7.658	119.408	177.85	66.65
81	L	8.237	120.776	----	57.90
83	R	7.133	117.194	179.70	58.79
84	V	8.087	124.460	180.95	65.39
85	D	8.907	123.906	178.28	57.30
86	Q	7.360	115.663	176.51	55.78
87	G	8.255	109.306	174.95	45.86
88	K	8.073	117.953	173.82	55.60
89	E	7.319	118.987	171.83	53.23
90	N	10.111	126.020	175.27	52.27
91	L	8.971	124.953	176.48	57.78
92	D	8.163	113.457	175.76	53.40
93	R	7.368	123.217	----	57.85
95	I	9.114	132.074	175.02	61.33
96	T	8.024	118.752	173.14	60.56
97	Y	7.737	119.724	173.14	55.92
98	G	9.338	107.714	175.24	43.71
99	K	8.721	120.729	179.39	59.39
100	E	9.480	118.128	176.70	58.43
101	D	7.626	117.954	175.26	55.44
102	L	7.011	117.101	178.11	55.18
103	V	7.663	117.925	176.43	58.94
104	D	8.264	123.449	----	56.04
106	S	8.333	120.447	----	55.76
109	T	9.432	113.692	182.24	64.03
110	E	8.117	121.034	176.77	58.98
111	K	7.173	116.786	177.14	56.34
112	H	7.099	115.113	173.79	54.91
113	V	7.165	119.535	176.92	54.96
114	G	8.313	109.674	----	46.18
116	G	7.922	109.805	171.90	45.33
117	M	8.192	114.607	174.90	54.88
118	T	8.975	112.552	175.88	60.43
119	V	8.130	121.714	178.77	67.98
120	A	9.153	122.346	179.78	56.26
121	E	7.733	118.560	----	58.79
123	C	8.276	120.577	----	64.35
125	A	8.281	124.261	----	55.52

127	I	7.608	112.330	175.83	64.07
128	T	8.413	107.477	174.42	64.82
129	Y	6.847	113.804	----	58.21
131	D	7.378	122.780	176.97	56.28
132	N	8.784	127.274	----	56.32
134	A	8.844	123.242	178.32	55.19
135	A	7.059	115.471	178.12	54.60
136	N	7.814	119.484	178.90	55.98
137	L	8.788	122.073	180.88	58.18
138	L	7.905	120.411	----	57.15
140	E	8.695	122.691	179.32	60.10
141	A	7.623	122.627	179.51	54.56
142	L	7.688	116.694	176.47	55.48
143	G	7.438	102.519	175.18	44.59
144	G	8.405	109.943	----	44.97
146	A	8.540	119.324	180.64	55.08
147	A	7.209	122.248	181.44	54.27
148	L	7.376	121.370	178.14	57.58
149	T	8.203	118.162	175.03	68.33
150	A	8.020	122.696	----	55.42
152	L	8.001	122.086	178.70	57.54
153	R	8.316	118.615	181.17	57.39
154	S	7.862	117.246	175.10	61.27
155	I	6.946	113.974	----	60.58
157	D	7.729	121.725	175.57	52.65
158	N	8.397	123.248	----	52.84
160	T	9.723	126.254	174.10	64.97
161	R	8.643	121.654	171.08	54.45
162	L	7.509	122.188	174.05	53.28
163	D	9.935	127.453	175.51	55.42
164	R	9.058	125.196	170.48	55.31
165	W	7.599	114.626	177.80	55.21
166	E	9.271	117.460	----	54.63
169	L	7.834	115.671	176.59	57.42
170	N	7.653	120.441	174.46	54.01
171	T	7.569	114.661	174.33	67.22
172	A	9.267	121.242	175.50	51.96
173	A	8.229	121.245	----	50.63
175	G	8.830	113.723	173.19	45.23
176	D	7.220	122.851	----	50.28
180	T	7.144	105.756	178.04	59.59
181	T	8.081	114.174	173.50	59.74
182	T	8.377	112.298	----	57.69
186	M	8.269	117.210	178.31	57.27
187	A	8.043	119.617	----	55.92

189	T	9.098	121.762	175.93	66.80
193	L	8.106	118.229	----	67.83
196	G	7.572	110.135	174.26	44.98
197	D	8.333	118.882	176.13	53.71
198	V	7.027	117.878	----	64.47
200	S	9.357	120.040	----	57.55
202	A	8.347	119.656	181.06	55.04
203	S	7.961	120.158	---	62.67
206	Q	7.508	118.865	177.56	57.22
207	L	7.690	120.506	178.25	58.06
208	V	7.918	118.989	177.00	68.30
209	D	8.394	119.705	180.86	57.75
210	W	7.925	121.231	176.89	59.19
211	L	8.030	117.588	181.75	58.39
212	I	8.985	122.962	177.64	64.56
213	A	7.443	122.220	176.05	51.87
214	N	7.117	116.321	177.19	56.58
215	K	8.698	126.691	----	55.74
217	G	8.922	113.129	176.96	45.85
218	D	8.822	123.950	176.62	58.07
219	K	8.362	114.391	175.67	54.99
220	R	6.781	118.142	----	54.39
222	R	9.306	115.172	177.90	59.80
223	A	6.977	118.079	178.51	53.50
224	G	7.745	105.160	172.45	44.55
225	L	6.607	120.454	----	51.58
227	A	8.463	124.905	177.71	54.39
228	D	8.076	112.141	----	53.70
230	R	7.825	125.735	----	55.72
232	G	8.916	111.786	171.72	43.77
233	D	8.184	122.161	169.57	53.22
234	K	8.316	113.968	----	54.23
236	G	7.619	103.530	----	48.54
238	G	8.541	113.050	172.81	45.95
240	E	7.427	117.163	----	56.27
242	G	8.633	104.959	175.07	47.46
243	T	6.559	115.088	175.27	63.08
244	T	9.225	127.499	----	63.73
246	D	9.260	122.456	172.25	54.97
247	I	7.981	119.725	173.72	58.00
248	A	9.373	122.831	174.35	50.66
249	V	8.450	119.176	171.93	58.93
250	V	8.278	125.908	174.46	60.01
251	W	9.616	127.860	----	56.15
256	H	7.80		116.64	173.33

257	A	8.165	123.312	----	50.76
260	F	8.840	125.846	----	60.80
262	A	8.148	127.184	----	51.44
264	Y	9.191	128.610	----	56.54
266	T	9.148	119.390	----	60.91
268	S	7.439	109.787	----	58.56
270	V	7.119	112.863	175.14	60.05
271	D	8.145	120.113	175.43	54.00
272	A	8.372	120.929	180.04	56.58
273	D	8.171	118.150	178.71	57.27
274	A	8.026	126.157	180.97	54.67
275	R	8.555	120.585	178.52	61.02
276	D	7.597	120.184	177.09	58.49
277	A	7.500	119.749	181.25	54.39
278	V	7.684	121.934	----	66.70
280	A	7.981	121.323	179.34	55.87
281	E	7.784	118.738	----	58.46
283	A	7.067	119.085	175.43	55.85
284	R	7.525	116.572	----	60.02
287	V	8.147	118.877	176.60	66.68
288	A	7.079	118.239	----	54.89

Table S3. Allowed sequence space for the α-set.

Original Residue	FuncLib Predictions
A280	A
A283	AT
R284	RAK

Table S4. FuncLib[5] predictions for the α-set.[a]

**Serial Number**	**Total Score**[b]
'010101	-905.347
'030402	-901.238
'030305	-900.447
'040302	-899.345
'030204	-898.554
'040405	-898.3
'020205	-896.248
'020303	-895.853
'020406	-895.197
'040203	-895.011

[a] Note that serial number '010101 refers to the initial crystal structure. For the full prediction set please see **Supplementary Data Table 1**. [b] Total FuncLib score presented in Rosetta Energy Units.

Table S5. Michaelis-Menten parameters and denaturation temperatures at pH 8.5 for Kemp eliminases studied in this work

**β-set variants**				
**Variant code**	k_{cat} **(s^{-1})**	K_M **(mM)**	k_{cat}/K_M **($M^{-1}s^{-1}$)**	T_m **(ºC)**
V4	610±63	3.3±0.4	167000±4000	80.3
V4-1	280±60	3.2±0.9	83000±5000	81.1
V4-2	140±40	3.0±1.1	46000±4000	85.7
V4-3	120±40	2.3±1.0	51000±6000	84.2
V4-4	1700±230	3.9±0.7	430000±10000	82.9
V4-5	170±30	2.6±0.7	6500±4000	85.1
V4-6	380±50	2.2±0.4	170000±7000	83.9
V4-7	660±40	3.4±0.4	195000±3000	85.4
V4-8	180±40	1.8±0.5	98000±9000	84.8
V4-9	210±130	3.2±2.5	62000±10000	83.6
V4-10	590±70	3.4±0.5	172000±5000	81.7
V4-11	930±100	2.5±0.4	375000±13000	81.7
V4-12	40±14	2.5±1.1	17000±2000	84.0
V4-13	670±80	2.3±0.4	296000±13000	84
V4-14	20±6	1.6±0.7	11000±2000	84.4
V4-15	290±40	2.3±0.5	126000±6000	83.8
V4-16	30±14	1.9±1.4	13000±3000	85.2
V4-17	110±20	2.1±1.8	49000±3000	82.4
V4-18	870±200	2.7±0.8	318000±22000	84
V4-19	690±160	2.6±0.8	262000±18000	84.9
V4-20	60±30	2.4±1.5	25000±4000	86.4
**α-set variants**				
**Variant code**	k_{cat} **(s^{-1})**	K_M **(mM)**	k_{cat}/K_M **($M^{-1}s^{-1}$)**	T_m **(ºC)**
V4-TK	110±50	4.0±2.3	27000±2600	80.5
V4-AA	300±20	2.9±0.2	105000±2000	80.9
V4-TR	500±190	3.2±1.5	156000±1400	81.1
V4-AK	90±10	2.1±0.5	42000±3000	79.9
V4-TA	300±100	3.5±1.5	84000±7000	81.5

Table S6. Allowed sequence space for the β-set.

**Original Residue**	**FuncLib Predictions**
L49	LFHIMRVWY
T51	TAMS
G58	GADEHNQST
R230	RIKMQTV
G232	GA
A248	A
W251	WFY
A257	AEGKPQRS
A262	ACSTV

Table S7. FuncLib predictions for the β-set.[a]

Variant FuncLib ID	Variant name	Total Score[b]
'010101010101010101	V4	-887.017
'040107040101020501	V4-1	-902.97
'040109040101030102	V4-2	-902.563
'040109060101020501	V4-3	-902.427
'040107020101010502	V4-4	-902.106
'040105070101030105	V4-5	-901.843
'040105040101010505	V4-6	-901.839
'040104020101030102	V4-7	-901.781
'040107060101020102	V4-8	-901.751
'040109040101030501	V4-9	-901.611
'090105020101020501	V4-10	-901.607
'070107070101010502	V4-11	-901.518
'070105010101020505	V4-12	-901.479
'040105020101010102	V4-13	-901.338
'040302060101010105	V4-14	-901.329
'070105040101010305	V4-15	-901.294
'040304040101010105	V4-16	-901.277
'040103010201030501	V4-17	-901.275
'040102070101010505	V4-18	-901.218
'040109060101010502	V4-19	-901.191
'040105040101020105	V4-20	-901.104

[a] Note that serial number '010101 refers to the initial crystal structure. Shown here are the initial crystal structure and the top 20 variants from the prediction. For the full prediction set please see **Supplementary Data Table 2**. [b] Total FuncLib score presented in Rosetta Energy Units.

Table S8. Purification yields of Kemp eliminase variants.[a]

Protein Variant	Protein concentration after purification (mg/ml)
V4	3.8
V4-4	19.5
V4-11	11.3
V4-12	6.0
V4-13	7.0
V4-14	3.0
V4-15	16.9
V4-16	12.9
V4-17	9.3
V4-18	5.4
V4-19	14.3
V4-20	10.9

[a] In all cases, the same purification protocol was used, starting with 400 mL of cell culture to obtain 3.2 mL of a purified protein solution. The protein concentrations in the final solutions are given.

Table S9. Catalytic parameters for Kemp eliminases V4 and V4-4 at different pH values

	V4			V4-4		
pH	k_{cat} (s^{-1})	K_M (mM)	k_{cat}/K_M ($M^{-1}s^{-1}$)	k_{cat} (s^{-1})	K_M (mM)	k_{cat}/K_M ($M^{-1}s^{-1}$)
6.0	80±10	2.7±0.6	29000±1000	110±10	2.5±0.4	42000±2000
6.5	90±20	1.6±0.6	53000±6000	130±10	1.3±0.1	97000±3000
7.0	210±50	2.2±0.7	93000±8000	280±30	1.6±0.2	173000±8000
7.5	220±20	1.6±0.3	137000±7000	400±30	1.1±0.2	349000±19000
8.0	330±40	2.2±0.4	154000±7000	790±140	2.2±0.5	359000±22000
8.25	ND	ND	ND	1100±110	4.2±1.5	409000±11000
8.5	610±63	3.3±0.4	167000±4000	1700±230	3.9±0.7	410000±10000

Table S10. Experimental and calculated activation free energies for the Kemp elimination of 5-nitrobenzisoxazole by different GNCA variants.[a]

System	Experimental	Calculated, "IN" substrate conformation	Calculated, "OUT" substrate conformation
GNCA4-WT	16.0	15.4 ± 1.2	18.5 ± 1.8
GNCA4-12	14.7	14.4 ± 0.9	17.3 ± 2.6
V4	13.6	14.9 ± 1.6	17.7 ± 2.9
V4-4	13.1	14.4 ± 1.3	19 .0 ± 1.9

[a] All energies are shown in kcal mol^{-1}. Experimental activation free energies were obtained from k_{cat} values presented in **Table S1**, using transition state theory. Calculated values are average values and standard deviations over 30 independent empirical valence bond (EVB) simulations[6] per system, with simulations initiated from either the "IN" or "OUT" substrate conformations, as described in the **Experimental Section**. EVB simulations where initiated from crystal structures were available (PDB ID: 5FQK[2] for GNCA4-WT, 6TXD[3] for GNCA4-12), or in the case of V4 and V4-4, the corresponding constructs, generated as described in the **Experimental Section**.

Table S11. Description of starting structures used for the MD simulations performed in this work, and modifications made to them during simulation setup.

**Variant**	**Initial Structure**	**Modifications**	**Non-Standard Protonation States[a]**
GNCA4-WT[2]	5FQK	Substrate manually placed in the active site.	Asp218, Lys207, HIE262, HIE263, HID85, HID213
GNCA4-12[3]	6TXD	Substrate manually placed in the active site.	Asp218, Lys207, HIE262, HIE263, HID85, HID213, HIP227
V4[4]	6TXD	Polypeptide segment added manually to the GNCA4-12 structure. Substrate manually placed in the active site.	Asp218, Lys209, HIE273-278, HID85, HID215, HIP229
V4-4	6TXD	Polypeptide segment added manually to the GNCA4-12 structure, individual residues mutated. Substrate manually placed in the active site.	Asp218, Lys209, HIE273-278, HID85, HID215, HIP229

[a] The side chains of aspartic acid residues listed here were protonated in our simulations, and those of lysine residues listed here were deprotonated. Protonation patterns of key histidine side chains are also provided.

Table S12. Non-standard force field parameters used to describe the substrate 5-nitrobenzisoxazole in our conventional molecular dynamics simulations.[a]

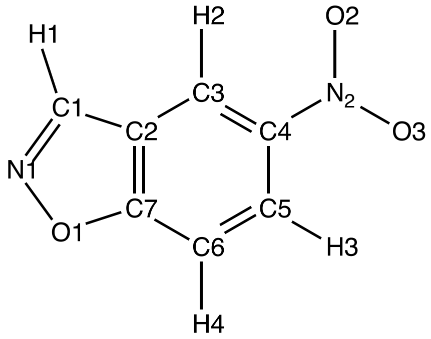

Atom Name	Atom Type	Partial Charge
O1	os	-0.199614
N1	nd	-0.313448
C1	cc	0.154120
H1	h4	0.151279
C2	ca	-0.050761
C3	ca	-0.250849
H2	ha	0.234663
C4	ca	-0.010267
N2	no	0.742907
O2	o	-0.450408
O3	o	-0.450408
C5	ca	-0.070430
H3	ha	0.198146
C6	ca	-0.432848
H4	ha	0.229481
C7	ca	0.518437

[a] All parameters were obtained using the General AMBER Force Field 2 (GAFF2),[7] as outlined in the Experimental Section in the main text.

Table S13. List of neutralized residues and histidine protonation patterns used in our EVB simulations of GNCA4-WT, GNCA4-12, V4 and V4-4 variants, in complex with substrates 5-nitrobenzisoxazole. [a]

**Residue Type**	**Residue Number**
Asp[b]	GNCA4-WT: 36, 58, 65, 74, 77, 88, 104, 130, 136, 149, 152, 170, 218, 242
	GNCA4-12: 36, 58, 65, 74, 77, 88, 104, 130, 136, 149, 152, 170, 218, 242
	V4: 38, 60, 67, 76, 79, 90, 106, 132, 138, 151, 154, 172, 220, 244
	V4-4: 38, 60, 67, 76, 79, 90, 106, 132, 138, 151, 154, 172, 220, 244
Glu	GNCA4-WT: 5, 9, 11, 37, 62, 73, 83, 94, 97, 113, 139, 141, 212, 238
	GNCA4-12: 5, 9, 11, 37, 62, 73, 83, 94, 97, 113, 139, 141, 212, 238
	V4: 7, 11, 13, 39, 64, 75, 85, 96, 99, 115, 141, 143, 214, 240
	V4-4: 7, 11, 13, 39, 64, 75, 85, 96, 99, 115, 141, 143, 214, 240
Lys	GNCA4-WT: 12, 46, 61, 72, 84, 207
	GNCA4-12: 12, 46, 61, 72, 84, 207
	V4: 14, 48, 63, 74, 86, 209
	V4-4: 14, 48, 63, 74, 86, 209
Arg	GNCA4-WT: 13, 17, 34, 38, 56, 66, 67, 126, 134, 137, 151, 246
	GNCA4-12: 13, 17, 34, 38, 56, 66, 67, 126, 134, 137, 151, 246
	V4: 15, 19, 36, 40, 58, 68, 69, 128, 136, 139, 153, 248
	V4-4: 15, 19, 36, 40, 58, 68, 69, 128, 136, 139, 153, 248
His-δ	GNCA4-WT: 85, 213
	GNCA4-12: 85, 213
	V4: 87, 215
	V4-4: 87, 215
His-ε	GNCA4-WT: 262, 263
	GNCA4-12: 263, 264, 265, 266
	V4: 273, 274, 275, 276, 277, 278
	V4-4: 273, 274, 275, 276, 277, 278

His-δ and ε	GNCA4-12: 227<br>V4: 229<br>V4-4: 229

[a] Shown here are the residues that fall outside the explicit simulation sphere, and were thus kept in their neutral form to avoid system instabilities created by having charged residues outside the water droplet (this is standard practice for such simulations[8]). All other residues were kept in their usual ionization state at physiological pH. In the case of the histidine side chains, His-ε and His-δ indicate histidine side chains protonated at the $N_{\varepsilon 2}$ and $N_{\delta 1}$ nitrogen atoms, respectively, and His-δ and His-ε indicates a doubly protonated histidine side chain. [b] Note that D218 in GNCA4-WT and GNCA4-12, and the corresponding D220 in V4 and V4-4 are protonated in our simulations, as they are the respective catalytic acids in the reaction shown in **Figure 1**.

Table S14. Description of starting structures used for the MD simulations performed for the inferred chalcone isomerase (CHI) ancestor ancCC, and the wild-type phosphotriesterase from *Pseudomonas diminuta*.[a]

**System**	**Initial Structure**	**Non-Standard Protonation States**
CHI ancCC[b]	5WKR	His-ε: 1<br>His-δ: 60<br>doubly protonated: 151
PTE R0[c]	4PCP	His-ε: 123, 201, 257<br>His-δ: 55, 57, 230<br>doubly protonated: 254

[a] Residue numbering following PDB IDs: 5WKR[9] for CHI ancCC, and 4PCP[10] for wild-type PTE.

Table S15. Non-standard force field parameters used to describe chalconaringenin during molecular dynamics simulations of the CHI ancCC-chalconaringenin complex.[a]

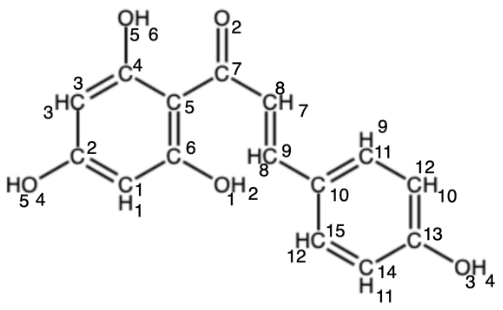

Atom Name	Atom Type	Partial Charge	Atom Name	Atom Type	Partial Charge
C1	ca	-0.685006	C13	ca	0.47076
O1	oh	-0.628861	C14	ca	-0.36609
C2	ca	0.594292	C15	ca	-0.108414
O2	o	-0.593425	H1	ha	0.26131
C3	ca	-0.685006	H2	ho	0.430795
O3	oh	-0.55392	H3	ha	0.26131
C4	ca	0.707883	H4	ho	0.390968
O4	oh	-0.512067	H5	ho	0.369299
C5	ca	-0.834148	H6	ho	0.430795
O5	oh	-0.628861	H7	ha	0.237326
C6	ca	0.707883	H8	ha	0.073933
C7	c	0.972703	H9	ha	0.16404
C8	ce	-0.733636	H10	ha	0.200529
C9	cf	0.16418	H11	ha	0.200529
C10	ca	0.001361	H12	ha	0.16404
C11	ca	-0.108414			
C12	ca	-0.36609			

[a] All parameters were obtained using the General AMBER Force Field 2 (GAFF2),[7] as outlined in the Experimental Section in the main text.

Table S16. Non-standard force field parameters used to describe the carbamylated lysine residue in our conventional molecular dynamics simulations of wild-type PTE.[a]

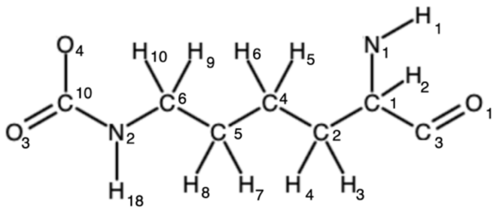

Atom Name	Atom Type	Partial Charge
N1	N	-0.551458
H1	H	0.265785
C1	CT	0.434447
H2	H1	-0.001470
C2	CT	-0.178318
H3	HC	0.045360
H4	HC	0.045360
C4	CT	-0.090695
H5	HC	0.055604
H6	HC	0.055604
C5	CT	-0.046077
H7	HC	0.044324
H8	HC	0.044324
C6	CT	0.234075
H9	H1	-0.021391
H10	H1	-0.021391
N2	N	-0.755002
H18	H	0.253962
C10	C	0.955468
O3	O2	-0.801442
O4	O2	-0.801442
C3	C	0.345469
O1	O	-0.511098

[a] All parameters were obtained using the ff19SB force field,[11] as outlined in the **Supplementary Computational Methods**.

Table S17. Non-standard force field parameters used to describe the PTE substrate 2-naphthyl-hexanoate in our conventional molecular dynamics simulations.[a]

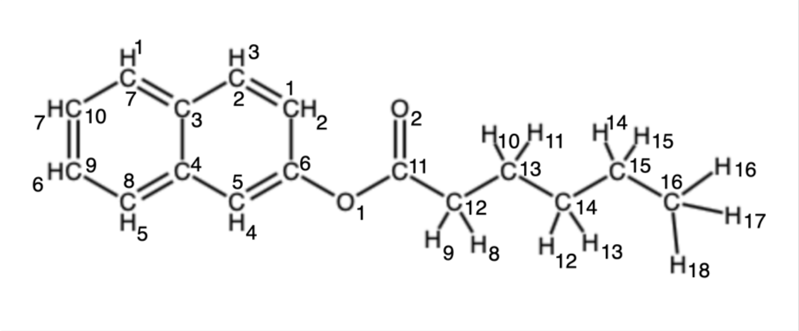

Atom Name	Atom Type	Partial Charge
C16	c3	-0.273109
H16	hc	0.061855
H17	hc	0.061855
H18	hc	0.061855
C15	c3	0.146985
H14	hc	-0.018254
H15	hc	-0.018254
C14	c3	-0.009978
H12	hc	0.006805
H13	hc	0.006805
C13	c3	0.064026
H10	hc	0.000453
H11	hc	0.000453
C12	c3	-0.365468
H8	hc	0.104828
H9	hc	0.104828
C11	c	1.004919
O2	o	-0.642649
O1	os	-0.597323
C6	ca	0.589678
C5	ca	-0.525546
H4	ha	0.226444
C1	ca	-0.448479

H2	ha	0.269432
H3	ha	0.168691
C3	ca	0.120233
C4	ca	0.261492
C8	ca	-0.268030
H5	ha	0.163135
C9	ca	-0.138781
H6	ha	0.147689
C10	ca	-0.129242
H7	ha	0.142572
C7	ca	-0.254155
H1	ha	0.160207

[a] All parameters were obtained using the General AMBER Force Field 2 (GAFF2),[7] as outlined in the Experimental Section in the main text.

S2. Supplementary Figures

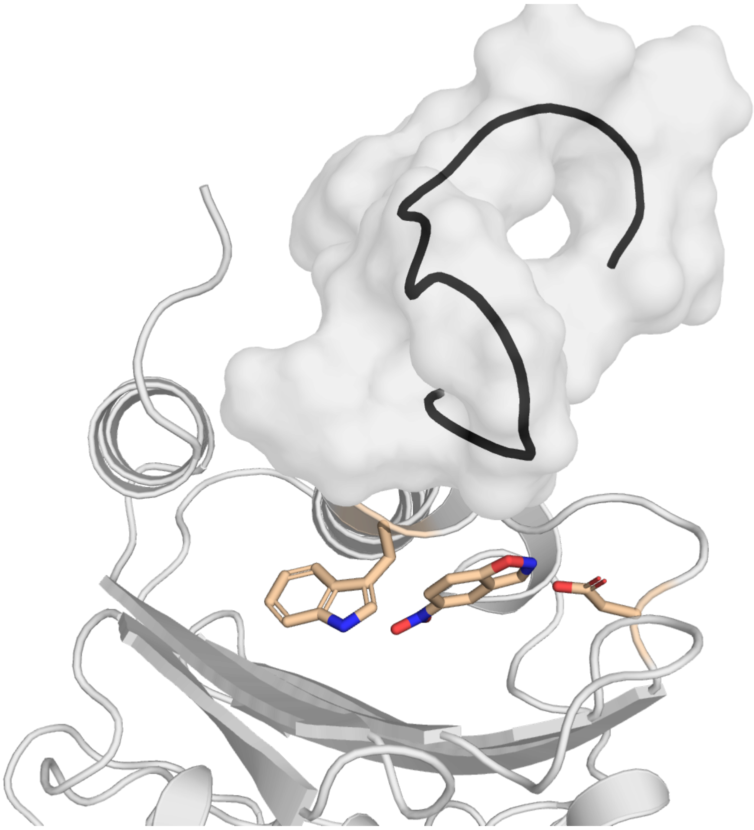

Figure S1: Representative structure of V4-4 plus the polypeptide segment showing the Modeller[12] predicted position of the C-terminal polypeptide. The extra polypeptide segment is highlighted in black, and the active site and substrate are colored light orange.

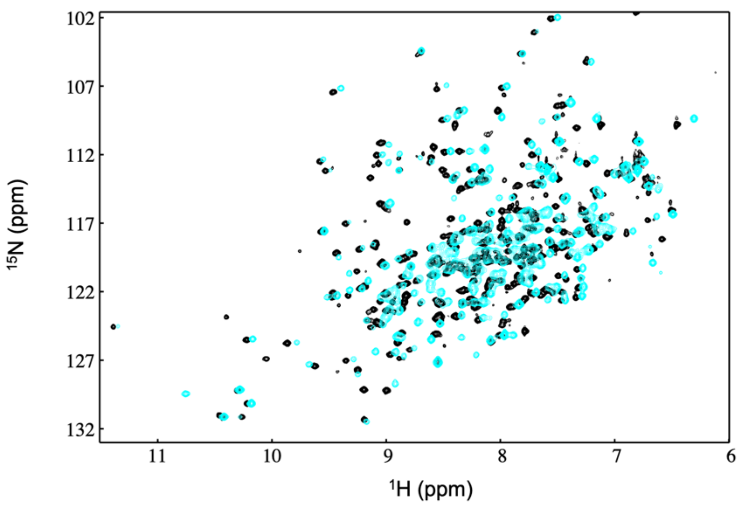

Figure S2. Overlay of 2D ^{1}H,^{15}N HSQC spectra of ^{13}C,^{15}N labeled sample of GNCA4-12 (cyan contours) and ^{15}N,^{13}C- GNCA$_{MP}$ (black contours).

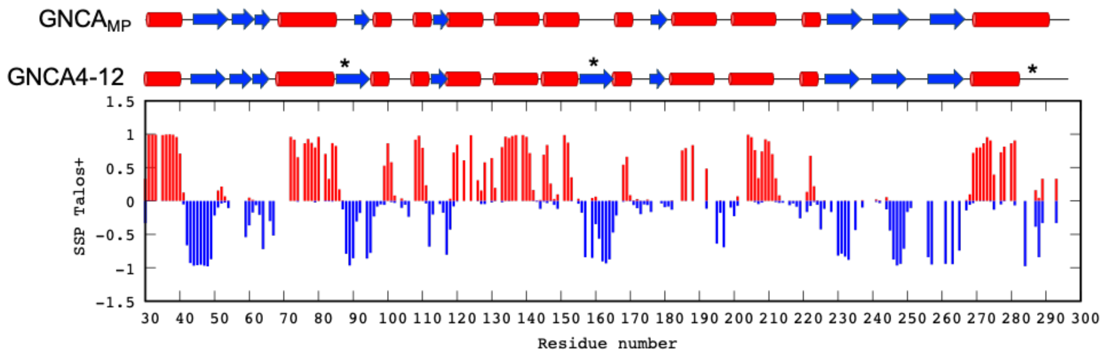

Figure S3. Bar plot of the helix (red; positive values) and strand (blue; negative values) probabilities predicted from chemical shifts of GNCA4-12 by using Talos+.[13] At the top the secondary structure elements of crystalline GNCA$_{MP}$ as well as that deduced for GNCA4-12 are indicated. Asterisks highlight the differences.

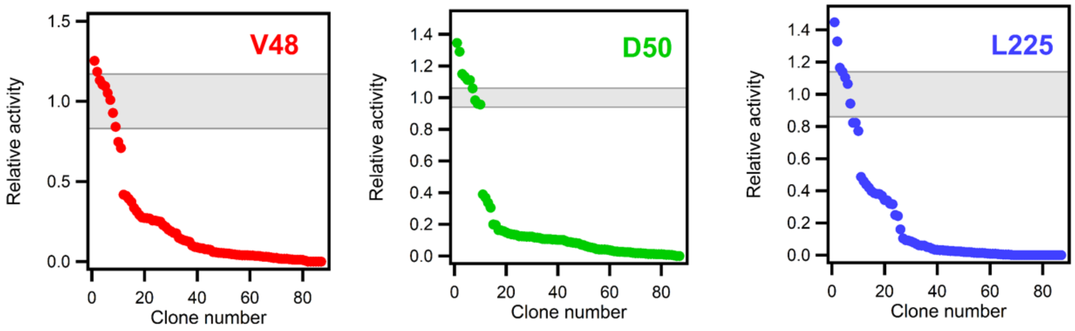

Figure S4. Saturation mutagenesis at the position of the active-site set, showing that no significant enhancement of activity was observed at either of the three positions. Clones are ranked in the graph according to the Kemp eliminase activity relative to the background variant. The grey strip represents the average activity of the background variant plus minus the associated standard error.

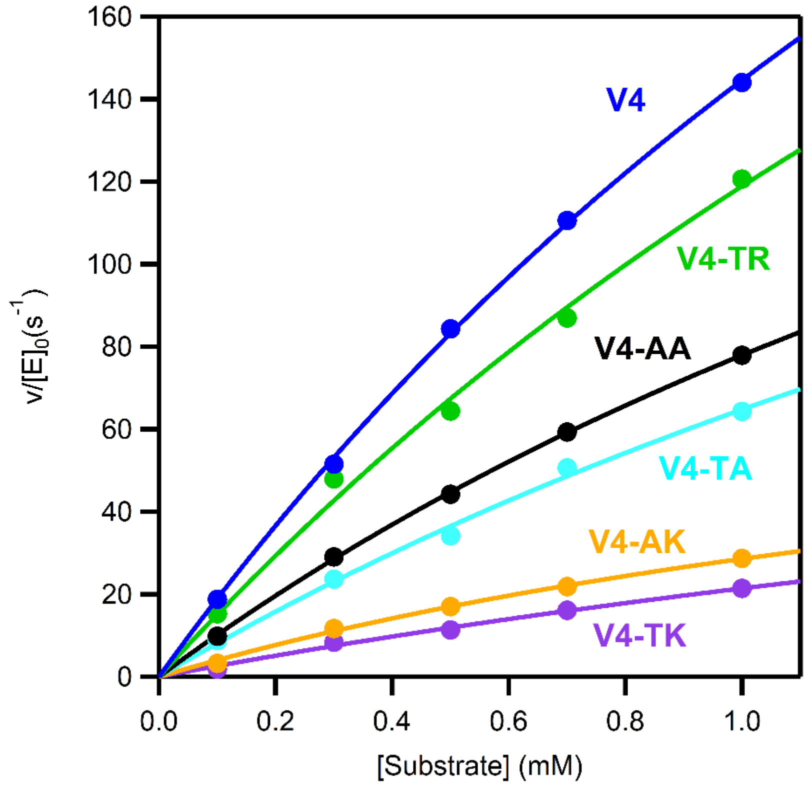

Figure S5. Experimental rate *vs*. substrate concentration for the variants of the α-set (profiles for the variants in the β-set are given in the main text). The terminology used describes the amino acids present at positions 283 and 284 of the studied variants of the V4 scaffold. Note that all variants are less active that the V4 background.

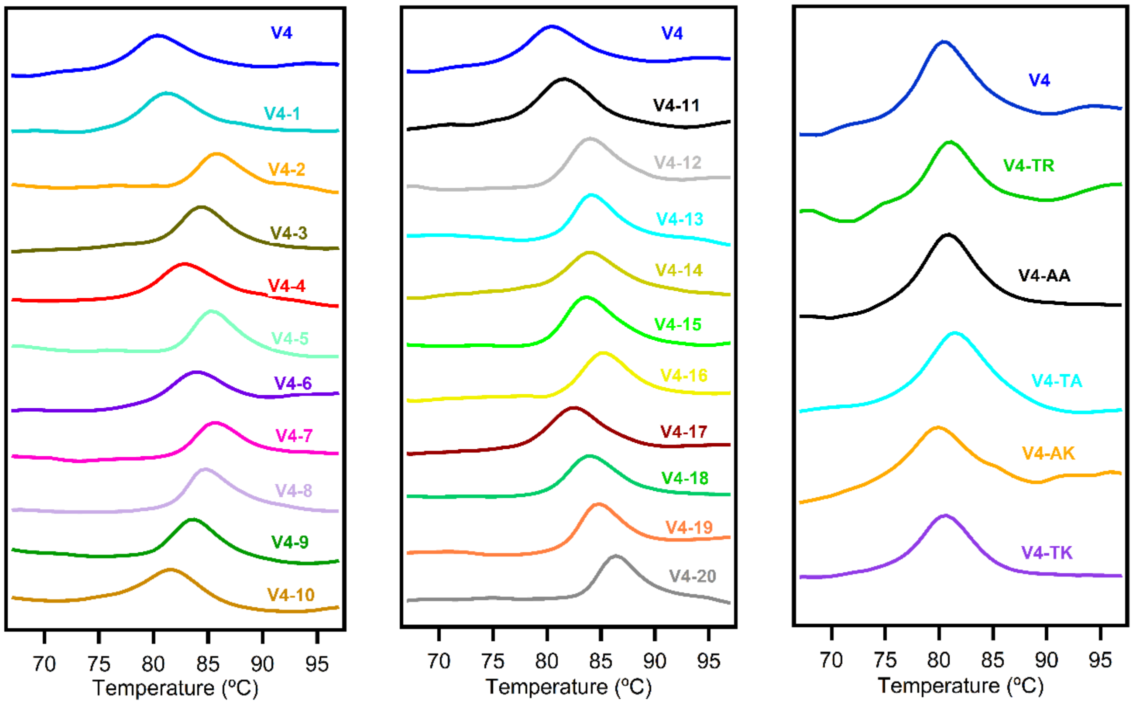

Figure S6. Differential scanning calorimetry profiles for selected protein variants.

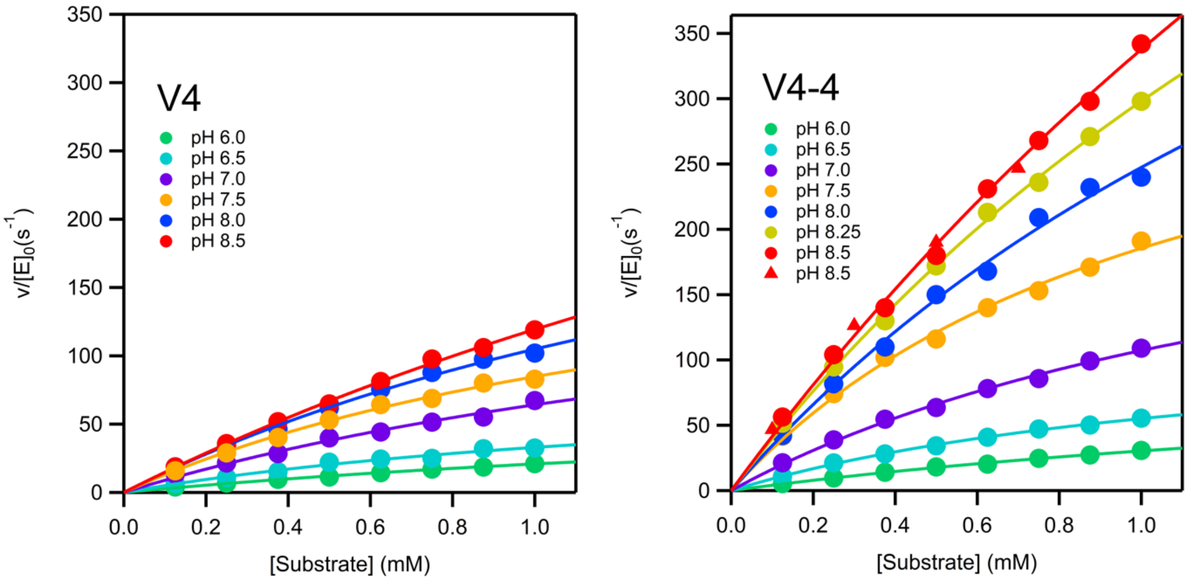

Figure S7. Examples of profiles of rate *vs*. substrate concentration for the background V4 variant and the best Kemp eliminase variant from this work (V4-4) at different pH values.

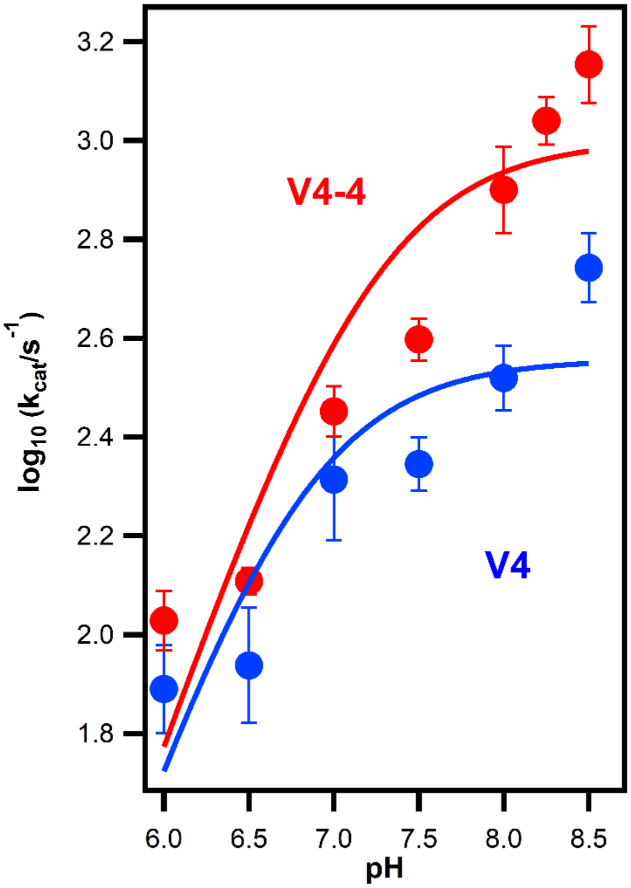

Figure S8. Effect of pH on the catalytic rate constant values for the best Kemp eliminase achieved in this work (variant V4-4) and the background protein V4. The values have been determined from the fitting of the Michaelis-Menten equation to profiles of rate versus substrate concentration obtained at different pH values (Figure S7). Error bars are standard deviations from the fittings of the Michaelis-Menten equation. The lines represent the best fits of the single-p*K* model described by equation 1 in the main text. Note that these fits are not satisfactory, in particular for the most active variant, V4-4, because deviations of the experimental data from the best fit values are, in many cases, much higher than the associated uncertainties and furthermore because the deviations follow a systematic, non-random pattern.

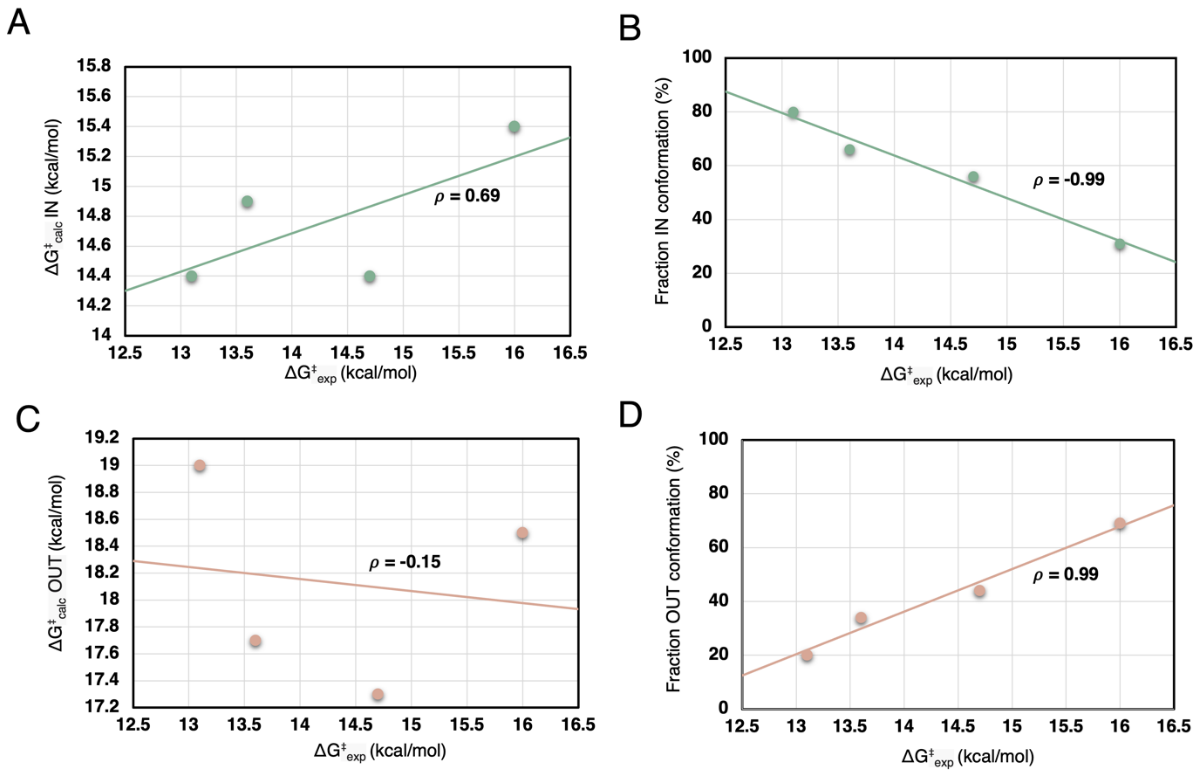

Figure S9. Pearson correlation coefficients for **(A, C)** calculated vs. experimental activation free energies (confidence interval <0.05), where calculated values are obtained from our empirical valence bond (EVB) simulations, and **(B, D)** the fraction of each substrate conformation observed in our MD simulations vs. experimental activation free energies. Panels **(A,B)** show data for the "IN" substrate conformation, while **(C,D)** correspond to the "OUT" substrate conformation. The raw data used in this figure is provided in Figure 7 and Table S10.

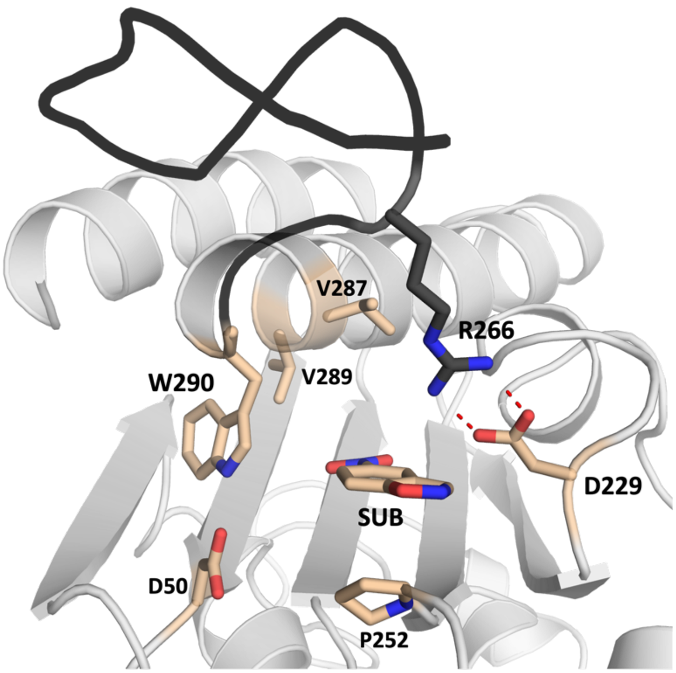

Figure S10. Structure of the V4-4 Kemp eliminase, with the polypeptide segment covering the active site, extracted as a snapshot from our molecular dynamics simulations of this variant.

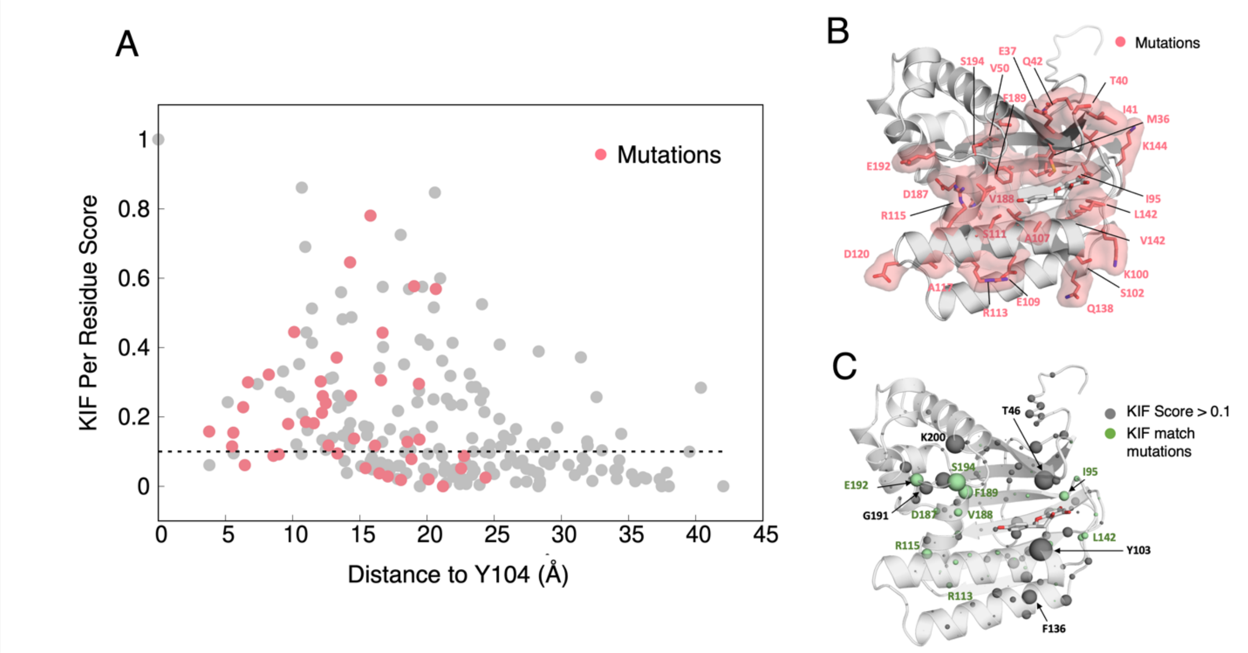

Figure S11. Prediction of residues involved in functionally important non-covalent interactions in CHI ancCC using Key Interactions Finder (KIF).[14] (**A**) Scatter plot of KIF-calculated residue importance scores, *vs*. distance to the active site (using the C_α-atom of Y104 as a proxy). Evolutionary important hotspots identified in prior work[9] are colored based on the set they are included in. (**B**) Structure of CHI ancCC (PDB ID: 5WKR[9]), showcasing the positions of the different evolutionary hotspots on the ancCC crystal structure. (**C**) Projection of residues with KIF scores >0.1 onto the ancCC crystal structure (PDB ID: 5WKR[9] chain A), shown as gray spheres, with evolutionary important hotspots with KIF scores >0.1 highlighted in green.

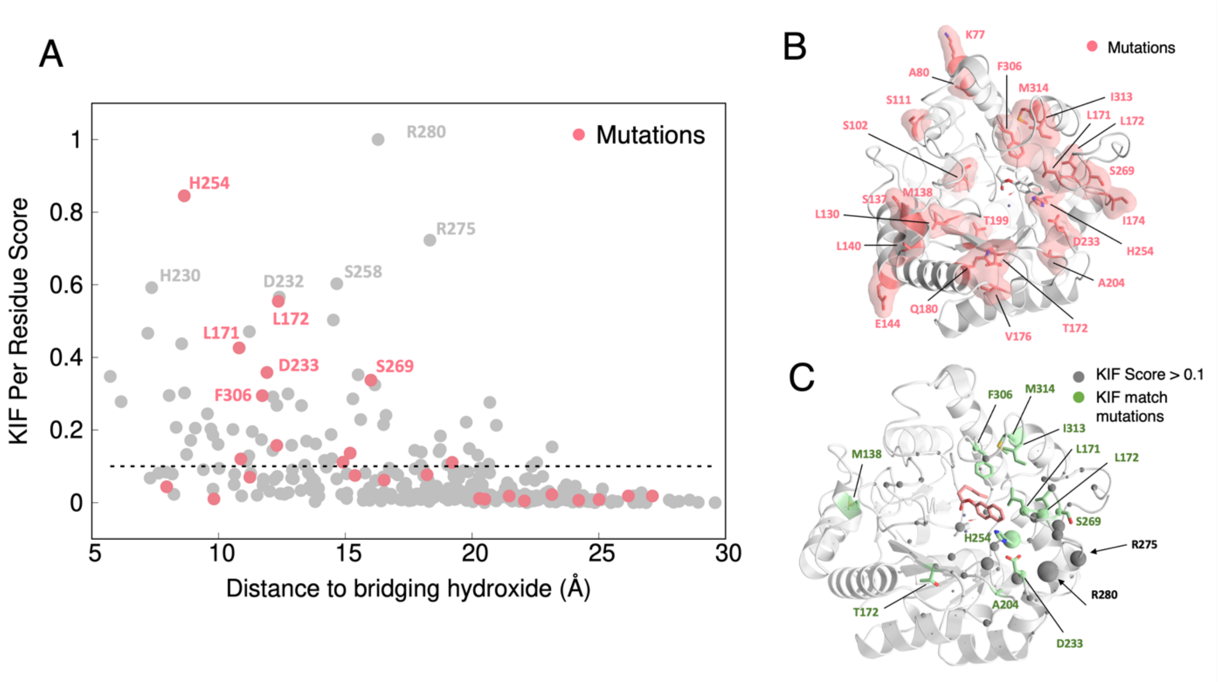

Figure S12. Prediction of residues involved in functionally important non-covalent interactions in the wild-type phosphotriesterase (PTE) from *Pseudomonas diminuta*, using Key Interactions Finder (KIF).[14] (A) Scatter plot of KIF-calculated residue importance scores, *vs*. distance to the PTE metal-bridging hydroxide oxygen atom. Hotspots identified from directed evolution[10] are colored based on the set they are included in. (**B**) Structure of PTE (PDB ID: 4PCP[10] showcasing the position of the different hotspots from directed evolution on the crystal structure. (**C**) Projection of residues with KIF scores >0.1 onto the PTE crystal structure (PDB ID: 4PCP[10]), shown as gray spheres, with evolutionary hotspots with KIF scores >0.1 highlighted in green.

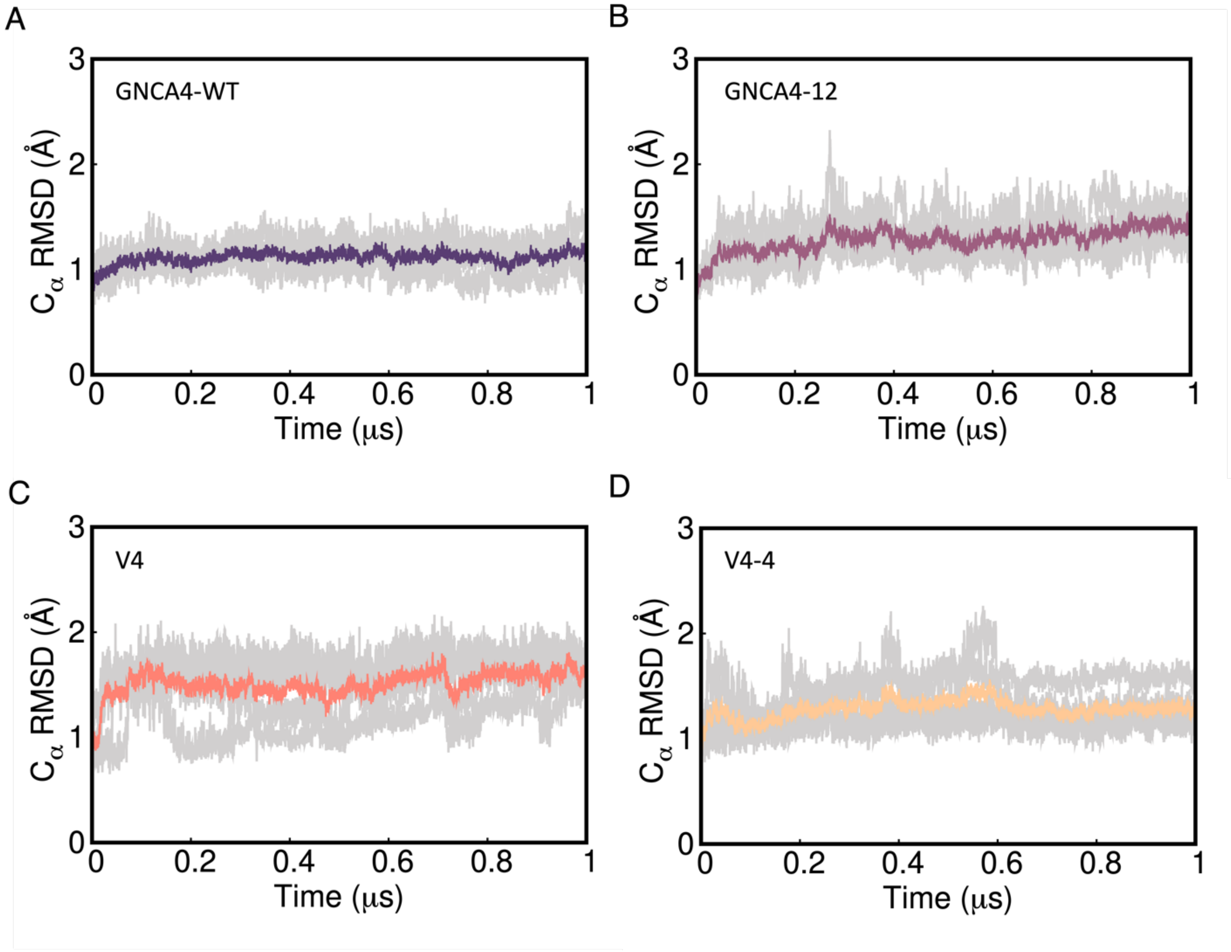

Figure S13. Root mean square deviations (RMSD, Å) of the C_α-atoms from MD simulations of (A) GNCA4-WT, (B) GNCA4-12 and (C) V4 and (D) V4-4 variants (polypeptide segment was not included in the RMSD analysis). Data was collected every 10 ps from 5 replicas of 1 μs length each. The grey lines show the 5 individual runs, whilst the solid line in the color of the respective variant in each panel shows a rolling average of the RMSD from all 5 replicas for each system.

S3. Supplementary Discussion

S3-1. pH-dependence of the Michaelis-Menten parameters for Kemp eliminases based on the proton-abstraction mechanism

The Michaelis-Menten equation can be easily justified on the basis of a simple pre-equilibrium assumption:

$$E + S \xleftrightarrow{K_M} ES \xrightarrow{k_{cat}} P$$

where S is the substrate, E is the free enzyme (*i.e.,* the enzyme with no substrate bound at the active site), ES is the Michaelis complex, and P is the product. k_{cat} is the first-order rate constant for the transformation of the Michaelis complex into products and K_M, the Michaelis constant, is the equilibrium constant for the formation of the Michaelis complex, although it is traditionally defined as a dissociation constant:

$$K_M = \frac{[E][S]}{[ES]} \qquad (1)$$

The rate of the reaction is,

$$v = k_{cat}[ES] \qquad (2)$$

and the concentration of the Michaelis complex can be easily expressed in terms of the substrate concentration and the total enzyme concentration,

$$[E]_T = [E] + [ES] \qquad (3)$$

using the expression for the Michaelis constant. That is, equation 1 is solved for [E], the resulting expression is substituted into equation 3, which is solved for [ES] to yield,

$$[ES] = [E]_T \frac{[S]}{K_M+[S]} \qquad (4)$$

which can be substituted into equation 2 to yield the Michaelis-Menten equation:

$$v = \frac{k_{cat}[E]_T[S]}{K_M+[S]} \qquad (5)$$

If the substrate concentration is clearly above the value of the Michaelis constant ($[S] >> K_M$), equation 5 reduces to,

$$v \approx k_{cat}[E]_T \qquad (6)$$

In this case, the reaction is of zero-order in substrate concentration and of first-order in enzyme concentration, and its rate is determined by the first-order rate constant k_{cat}, which is known as the catalytic constant or the turnover number.

If the substrate concentration is clearly below the value of the Michaelis constant ($[S] << K_M$), equation 5 reduces to,

$$v \approx \left(\frac{k_{cat}}{K_M}\right)[E]_T[S] \qquad (7)$$

In this case, the reaction is of global second order (first order in enzyme concentration and first order in substrate concentration) and its rate is determined by a second-order rate constant, k_{cat}/K_M, known as the catalytic efficiency.

For the *de novo* Kemp eliminases studied in this work, we expect the Michaelis-Menten catalytic parameters, k_{cat} and k_{cat}/K_M, to depend strongly on pH, since the reaction requires the aspartate at the active site to be deprotonated to function as the catalytic base. The normal pK_a values for an aspartate side chain is about 4, but a substantially higher value may be expected for an aspartate residue in a hydrophobic environment, as it is the case with the *de novo* active site in our Kemp eliminases. In any case, we may expect the values of the catalytic parameters to increase with pH,

as the aspartate becomes deprotonated. This effect of pH can be easily included in the pre-equilibrium model as expounded below.

We consider first the free enzyme, E, and distinguish between the free enzyme with the active-site aspartate deprotonated, E^0, and the free enzyme with the active-site aspartate protonated, E^+. The concentrations of E^0 and E^+ are related by the pK_a value of the active-site aspartic acid in the free enzyme (pK_a^E),

$$K_a^E = 10^{-pK_a^E} = \frac{[E^0]}{[E^+]}10^{-pH} \qquad (8)$$

from which it is straightforward to derive an expression for the fraction of free enzyme with a deprotonated aspartate:

$$X_E^0 = \frac{10^{\left(pH-pK_a^E\right)}}{1+10^{\left(pH-pK_a^E\right)}} \qquad (9)$$

Similarly, in the Michaelis-Menten complex, the active-site aspartate may be protonated, ES^0, or deprotonated, ES^+, with the concentrations of the forms related through the pK_a value of the active site aspartic acid in the Michaelis complex:

$$K_a^{ES} = 10^{-pK_a^{ES}} = \frac{[ES^0]}{[ES^+]}10^{-pH} \qquad (10)$$

and an expression for the fraction of Michaelis complex with a deprotonated aspartate is easily derived:

$$X_{ES}^0 = \frac{10^{\left(pH-pK_a^{ES}\right)}}{1+10^{\left(pH-pK_a^{ES}\right)}} \qquad (11)$$

It is important to note immediately that the pK_a values for the aspartic acid in the free enzyme (pK_a^E) and the Michaelis complex (pK_a^{ES}) are not necessarily equal. In fact, they are likely to be

different, since they are perturbed pK_a values that reflect the local environment of the active-site aspartic acid, and the local environment is bound to change upon substrate binding.

Only the Michaelis complex with deprotonated active-site aspartic acid is competent for the chemical reaction. Therefore, the rate of the reaction is given by,

$$v = k[ES^0] = kX_{ES}^0[ES] \qquad (12)$$

where k is the (pH-independent) rate constant for the conversion of ES^0 into products. Comparison of equation 12 with equation 2 immediately yields an equation for the pH-dependence of k_{cat},

$$k_{cat} = kX_{ES}^0 = k\frac{10^{\left(pH-pK_a^{ES}\right)}}{1+10^{\left(pH-pK_a^{ES}\right)}} \qquad (13)$$

or, taking logarithms,

$$log_{10}k_{cat} = log_{10}k + log_{10}\frac{10^{\left(pH-pK_a^{ES}\right)}}{1+10^{\left(pH-pK_a^{ES}\right)}} \qquad (14)$$

which is, in fact, equation 1 in the main text with P=k_{cat} and pK_a=pK_a^{ES}.

In order to determine an equation for the pH-dependence of the catalytic efficiency, k_{cat}/K_M, we need to consider the effect of pH on the Michaelis constant:

$$K_M = \frac{[E][S]}{[ES]} = \frac{\left({[E^0]}/{X_E^0}\right)[S]}{\left({[ES^0]}/{X_{ES}^0}\right)} = K\frac{X_{ES}^0}{X_E^0} \qquad (15)$$

where K=$[E^0][S]/[ES^0]$ is the (pH-independent) substrate-dissociation constant of the non-protonated Michaelis complex to give the non-protonated free enzyme. Using equations 12, 15 and 9, the pH-dependency of the catalytic efficiency can be easily written as,

$$\frac{k_{cat}}{K_M} = \frac{k}{K} X_E^0 = \left(\frac{k}{K}\right) \frac{10^{\left(pH - pK_a^E\right)}}{1+10^{\left(pH - pK_a^E\right)}} \qquad (16)$$

or, taking logarithms,

$$log_{10}\left({}^{k_{cat}}/_{K_M}\right) = log_{10}\left({}^{k}/_{K}\right) + log_{10}\left(\frac{10^{\left(pH - pK_a^E\right)}}{1+10^{\left(pH - pK_a^E\right)}}\right) \qquad (17)$$

which is, in fact, equation 1 in the main text with P=k_{cat} and pK_a=pK_a^E.

Equations 14 and 17 reveal that, while the pH-dependency of k_{cat} probes the ionization at the active-site in the Michaelis complex, the pH dependence of the catalytic efficiency, k_{cat}/K_M, probes the ionization in the free enzyme only.

The fact that ionization in the Michaelis complex does not determine the pH-dependency of the catalytic efficiency might seem surprising. Actually, it is not. It is a well-known textbook result that the value of the catalytic efficiency reflects the free energy of the transition state with respect to the reactants (free enzyme plus free substrate). Therefore, ionization in the Michaelis complex will necessarily affect k_{cat} and K_M by the same factor which will cancel out in the ratio k_{cat}/K_M. On the other hand, the value of k_{cat} reflects the free energy of the transition state with respect to the Michaelis complex and the pH-dependence of k_{cat} reflects ionization in the Michaelis complex.

The experimental catalytic efficiency data for our V4 and V4-4 Kemp eliminases are well described by equation 17 with a clearly perturbed pK_a value for the aspartic acid at the active site. On the other hand, as discussed in the main text, equation 14 does not describe the pH-dependence of k_{cat}. This means that that the ionization behavior in the Michaelis complex is more complicated that the simple model we have assumed in deriving equation 14. This is consistent with our computational analyses which show two conformations of the substrate bound in the Michaelis complex: "IN" and "OUT". Even if the two conformations are catalytically competent, they may bring about different environments in the *de novo* active site and, therefore, different perturbed pK_a values for the active site aspartic acid. Then, k_{cat} would be given by,

$$k_{cat} = k_{IN}X^{0}_{ES,IN} + k_{OUT}X^{0}_{ES,OUT} \qquad (18)$$

where k_{IN} and k_{OUT} are the first order rate constants for the conversion of the substrate in "IN" and "OUT" conformations into products. $X^{0}_{ES,IN}$ is the fraction of Michaelis complex with, simultaneously, the substrate in conformation "IN" and a deprotonated active-site aspartic acid residue. Likewise, $X^{0}_{ES,OUT}$ is the fraction of Michaelis complex with, simultaneously, the substrate in conformation "OUT" and a deprotonated active-site aspartic acid residue.

Analysis of the experimental k_{cat} *vs.* pH profile on the basis of equation 18, would require fitting 5 parameters: besides k_{IN} and k_{OUT}, the pK_a^{ES} values for the "IN" and "OUT" conformation, and the equilibrium constant for the "IN"↔"OUT" conversion at some specified condition. A unique, reliable fit is unlikely to be obtained 5 fitting parameters. Therefore, analysis of the experimental k_{cat} *vs.* pH profile on the basis of equation 18 was not pursued.

S3-2. The Pareto front

The enzyme enhancement addressed in this work is a two-objective problem, which precludes the identification of a unique "best" variant. The reason is obviously that each variant is given two scores (related to stability and catalysis) and there is no single variant that simultaneously displays the highest values for the two scores. Therefore, it is of interest in this context to briefly discuss how variant selection would be carried out when several properties are relevant for an intended application. In general, for multi-objective optimization problems, a global optimum cannot be generally identified. However, the Pareto front is a fundamental concept in engineering, including enzyme engineering, that, at a very basic level, provides a criterium to decide which variants within a set need to be considered for a practical application in a multi-objective optimization problem. A solution (enzyme variant in our case) belongs to the Pareto front if there is no other variant within the set experimentally characterized with better scores in all the targeted objectives. The Pareto front identifies the subset of variants to be explored for a practical application. This is so because variants within the set experimentally characterized that do not belong to the Pareto front will be worse than those in the front in terms of both properties and, therefore, are not worth considering. For illustration, we highlight in Figure 4 of the main text the catalysis/stability Pareto fronts for the set of 20 β-set Kemp eliminase variants studied in this work.

S4. Supplementary Computational Methods

System setup for additional molecular dynamics simulations. Additional molecular dynamics simulations have been performed on a non-catalytic chalcone isomerase ancestor inferred from ancestral sequence reconstruction[9] (CHI ancCC) and the wild-type phosphotriesterase from *Pseudomonas diminuta* (PTE), in both the liganded and unliganded forms of each protein, to assess the ability of interaction network tools to reliably predict known mutational hotspots.

Simulations of each system were initiated from available X-ray structures of ancCC (PDB ID: 5WKR,[9] Chain A) and PTE (PDB ID: 4PCP,[10] both chains). In the case of ancCC, the ligand was manually removed from the active site for simulations of the unliganded system. In contrast, in the case of PTE, the arylester substrate 2-naphthylhexanoate (the activity towards which this enzyme was evolved[10] in the evolutionary trajectory of interest) was docked into the active site using PLANTS.[15, 16] To perform this docking, the binding site was centred on the metal-bridging hydroxide ion, with a 12Å binding site radius. The $PLANTS_{CHEMPLP}$ scoring function was used, and the search speed was set to the lowest available, with the highest reliability. The standard PLANTS clustering parameters were applied. During evaluation of the clustering results, we also considered the coordination of the carbonyl oxygen of the substrates to the Zn(β) ion, as described by previous studies.[17-19]

The pK_as of ionizable residues in both systems were predicted using PROPKA 3.0,[20] coupled with visual examination of the local environments of histidine side chains. Based on this, all ionizable residues were kept in their standard protonation states at physiological pH throughout the simulations. The protonation patterns of histidine residue are summarized in Table S14 for each of the systems. In the case of PTE, the protonation state of zinc coordinating residues were set according to their position relative to the zinc ions. The two systems were then solvated in a truncated octahedral water box of OPC water molecules,[21] extending 11.0Å from the protein in all directions, and all systems were neutralized using Na^+ or Cl^- counterions (6 Na^+ for CHI ancCC, and 1 Cl^- for PTE). Finally, all hydrogen atoms in the system were scaled using hydrogen mass repartitioning.[22]

All additional MD simulations were performed using the CUDA-accelerated version of Amber22,[23] and the ff19SB force field.[11] Partial charges for the CHI ancCC ligand chalconaringenin were calculated at the HF/6-31G(d) level of theory by restrained electrostatic potential (RESP)[24] fitting using Antechamber,[25] based on gas-phase geometries optimized at the B3LYP/6-31G(d) level of theory, using Gaussian 16 Rev. B.01.[26] Partial charges for the 2-naphthyl-hexanoate substrate of PTE and the bridging hydroxide were optimized at the ωB97XD/6-31+G(d) level of theory using Gaussian 16 Rev. C.01.[26] The carbamylated lysine residue bridging the bivalent metal centre in the PTE active site was parameterized according to a custom protocol designed for Amber (see https://carlosramosg.com/amber-custom-residue-parameterization). The zinc ions were described using a 12-6-4 Lennard-Jones model.[27] All other force field parameters used to describe chalconaringenin and 2-naphthyl-hezaonate were obtained using the General AMBER Force Field (GAFF2).[7] All non-standard parameters used in our simulations are summarized in Tables S15 to S17.

All additional molecular dynamics simulations were performed using the same equilibration and production protocol as described for the Kemp eliminases in the main text. No additional restraints were placed on the CHI/PTE simulations. For all systems, 5 different replicas were performed, of 1 μs in length for CHI, and 500 ns in length for PTE.

S5. Supplementary References

(1) Risso, V. A.; Gavira, J. A.; Mejia-Carmona, D. F.; Guacher, E.; Sanchez-Ruiz, J. M. Hyperstability and Substrate Promiscuity in Laboratory Resurrections of Precambrian β-Lactamases. *Biochem. J.* **2013**, *429*, 243-249.

(2) Risso, V. A.; Martinez-Rodriguez, S. M.; Candel, A. M.; Krüger, D. M.; Pantoja-Uceda, D.; Ortega-Muñoz, M.; Santoyo-Gonzalez, F.; Gaucher, E. A.; Kamerlin, S. C. L.; Bruix, M.; et al. *De Novo* Active Sites for Resurrected Precambrian Enzymes. *Nat. Commun.* **2017**, *8*, 16113.

(3) Risso, V. A.; Romero-Riversa, A.; Gutierrez-Rus, L. I.; Ortega-Muñoz, M.; Santoyo-Gonzalez, F.; Gavira, J. A.; Sanchez-Ruiz, J. M.; Kamerlin, S. C. L. Enhancing a *De Novo* Enzyme Activity by Computationally-Focused Ultra-Low-Throughput Screening. *Chem. Sci.* **2020**, *11*, 6134-6148.

(4) Gutierrez-Rus, L. I.; Alcalde, M.; Risso, V. A.; Sanchez-Ruiz, J. M. Efficient Base-Catalyzed Kemp Elimination in an Engineered Ancestral Enzyme. *Int. J. Mol. Sci.* **2022**, *23*, 8394.

(5) Khersonsky, O.; Lipsh, R.; Avizemer, Z.; Ashani, Y.; Goldsmith, M.; Leader, H.; Dym, O.; Rogotner, S.; Trudeau, D. L.; Prilusky, J.; et al. Automated Design of Efficient and Functionally Diverse Enzyme Repertoires. *Mol Cell.* **2018**, *72*, 178-186.e175.

(6) Warshel, A.; Weiss, R. M. An Empirical Valence Bond Approach for Comparing Reactions in Solutions and in Enzymes. *J. Am. Chem. Soc.* **1980**, *102*, 6218-6226.

(7) Wang, J.; Wolf, R. M.; Caldwell, J. W.; Kollman, P. A.; Case, D. A. Development and Testing of a General Amber Force Field. *J. Comput. Chem.* **2004**, *25*, 1157-1174.

(8) Warshel, A.; King, G. Polarization Constraints in Molecular Dynamics Simulation of Aqueous Solutions: The Surface Constraint All Atom Solvent (SCAAS) Model. *Chem. Phys. Lett.* **1985**, *121*, 124-129.

(9) Kaltenbach, M.; Burke, J. R.; Dindo, M.; Pabis, A.; Munsberg, F. S.; Rabin, A.; Kamerlin, S. C. L.; Noel, J. P.; Tawfik, D. S. Evolution of Chalcone Isomerase from a Noncatalytic Ancestor. *Nat. Chem. Biol.* **2018**, *14*, 548-555.

(10) Campbell, E.; Kaltenbach, M.; Correy, G. J.; Carr, P. D.; Porebski, B. T.; Livingstone, E. K.; Afriat-Jurnou, L.; Buckle, A. M.; Weik, M.; Hollfelder, F.; et al. The Role of Protein Dynamics in the Evolution of New Enzyme Function. *Nat. Chem. Biol.* **2016**, *12*, 944-950.

(11) Tian, C.; Kasavajhala, K.; Belfon, K. A. A.; Raguette, L.; Huan, H.; Migues, A. N.; Bickel, J.; Wang, Y.; Pincay, J.; Wu, Q.; et al. ff19SB: Amino-Acid-Specific Protein Backbone Parameters

Trained against Quantum Mechanics Energy Surfaces in Solution. *J. Chem. Theory Comput.* **2020**, *16*, 528-552.

(12) Šali, A.; Blundell, T. L. Comparative Protein Modeling by Satisfaction of Spatial Restraints. *J. Mol. Biol.* **1993**, *234*, 779-815.

(13) Shen, Y.; Delaglio, F.; Cornilescu, G.; Bax, A. TALOS+: A Hybrid Method for Predicting Protein Backbone Torsion Angles from NMR Chemical Shifts. *J. Biomol. NMR* **2010**, *44*, 213-223.

(14) Crean, R. M.; Slusky, J. S. G.; Kasson, P. M.; Kamerlin, S. C. L. KIF – Key Interactions Finder: A Program to Identify the Key Molecular Interactions that Regulate Protein Conformational Changes. *J. Chem. Phys.* **2023**, *158*, 144114.

(15) Korb, O.; Stützle, T.; Exner, T. E. *PLANTS: Application of Ant Colony Optimization to Structure-Based Drug Design*; Springer, 2006.

(16) Korb, O.; Stützle, T.; Extner, T. E. An Ant Colony Optimization Approach to Flexible Docking. *Swarm Intell.* **2007**, *1*, 115-134.

(17) Vanhooke, J. L.; Benning, M. M.; Raushel, F. M.; Holden, H. M. Three-Dimensional Structure of the Zinc-Containing Phosphotriestease With the Bound Substrate Analog Diethyl 4-Methylbenzylphosphonate. *Biochemistry* **1996**, *35*, 6020-6025.

(18) Bora, R. P.; Mills, M. J.; Frushicheva, M. P.; Warshel, A. On the Challenges of Exploring the Evolutionary Trajecotry from Phopshotriesterase to Arylesterase Using Computer Simulations. *J. Phys. Chem. B* **2015**, *119*, 3434-3445.

(19) Bigley, A. N.; Raushel, F. M. Catalytic Mechanisms for Phosphotriesterases. *Biochim. Biophys. Acta* **2013**, *1834*, 443-453.

(20) Søndergaard, C. R.; Olsson, M. H. M.; Rostkowski, M.; Jensen, J. H. Improved Treatment of Ligands and Coupling Effects in Empirical Calculation and Rationalization of pK_a Values. *J. Chem. Theory Comput.* **2011**, *7*, 2284-2295.

(21) Izadi, S.; Anandakrishnan, R.; Onufriev, A. Building Water Molecules: A Different Approach. *5* **2014**, 3863-3871.

(22) Hopkins, C. W.; LeGrand, S. L.; Walker, R. C.; Roitberg, A. E. Long-Time-Step Molecular Dynamics Through Hydrogen Mass Repartitioning. *J. Chem. Theory Comput.* **2015**, *11*, 1864-1874.

(23) Case, D. A.; Aktulga, H. M.; Belfon, K.; Ben-Shalom, I. Y.; Berryman, J. T.; Brozell, S. R.; Cerutti, D. S.; Cheatham III, T. E.; Cisneros, G. A.; Cruzeiro, V. W. D.; *et al.* Amber 2022. University of California: San Francisco, 2022.
(24) Woods, R. J.; Chappelle, R. Restrained Electrostatic Potential Atomic Partial Charges for Condensed-Phase Simulations of Carbohydrates. *J. Mol. Struct.* **2000**, *1-3*, 149-156.
(25) Wang, J.; Wang, W.; Kollman, P. A.; Case, D. A. Automatic Atom Type and Bond Type Perception in Molecular Mechanical Calculations. *J. Mol. Graph. Model.* **2006**, *25*, 247-260.
(26) Frisch, M. J.; Trucks, G. W.; Schlegel, H. B.; Scuseria, G. E.; Robb, M. A.; Cheeseman, J. R.; Scalmani, G.; Barone, V.; Petersson, G. A.; Nakatsuji, H.; *et al.* Gaussian 16, Revision A.03; Gaussian Inc.: Wallingford CT, 2016.
(27) Li, P.; Song, L. F.; Merz Jr., K. M. Parameterization of Highly Charged Metal Ions Using the 12-6-4 LJ-Type Nonbonded Model in Explicit Water. *J. Phys. Chem. B* **2014**, *119*, 883-895.

Supplementary Data Table 1

serial_number	280A	283A	284A	total_score
'010101	A	A	R	-905.347
'030402	K	T	A	-901.238
'030305	K	S	K	-900.447
'040302	S	S	A	-899.345
'030204	K	G	H	-898.554
'040405	S	T	K	-898.3
'020205	G	G	K	-896.248
'020303	G	S	E	-895.853
'020406	G	T	Q	-895.197
'040203	S	G	E	-895.011

Supplementary Data Table 2

serial_number	49A	51A	58A	230A	232A	248A	251A	257A	262A	total_score
'010101010101010101	L	T	G	R	G	A	W	A	A	-887.017
'040107040101020501	I	T	Q	M	G	A	F	P	A	-902.97
'040109040101030102	I	T	T	M	G	A	Y	A	C	-902.563
'040109060101020501	I	T	T	T	G	A	F	P	A	-902.427
'040107020101010502	I	T	Q	I	G	A	W	P	C	-902.106
'040105070101030105	I	T	H	V	G	A	Y	A	V	-901.843
'040105040101010505	I	T	H	M	G	A	W	P	V	-901.839
'040104020101030102	I	T	E	I	G	A	Y	A	C	-901.781
'040107060101020102	I	T	Q	T	G	A	F	A	C	-901.751
'040109040101030501	I	T	T	M	G	A	Y	P	A	-901.611
'090105020101020501	Y	T	H	I	G	A	F	P	A	-901.607
'070107070101010502	V	T	Q	V	G	A	W	P	C	-901.518
'070105010101020505	V	T	H	R	G	A	F	P	V	-901.479
'040105020101010102	I	T	H	I	G	A	W	A	C	-901.338
'040302060101010105	I	M	A	T	G	A	W	A	V	-901.329
'070105040101010305	V	T	H	M	G	A	W	G	V	-901.294
'040304040101010105	I	M	E	M	G	A	W	A	V	-901.277
'040103010201030501	I	T	D	R	A	A	Y	P	A	-901.275
'040102070101010505	I	T	A	V	G	A	W	P	V	-901.218
'040109060101010502	I	T	T	T	G	A	W	P	C	-901.191
'040105040101020105	I	T	H	M	G	A	F	A	V	-901.104
'040102060101030501	I	T	A	T	G	A	Y	P	A	-901.054
'040107010101030505	I	T	Q	R	G	A	Y	P	V	-901.049
'010107060101020502	L	T	Q	T	G	A	F	P	C	-901.041
'040309020101030101	I	M	T	I	G	A	Y	A	A	-901.022
'070109020101030105	V	T	T	I	G	A	Y	A	V	-900.992
'040107060101030105	I	T	Q	T	G	A	Y	A	V	-900.99
'040109020101010505	I	T	T	I	G	A	W	P	V	-900.987
'070109010201020501	V	T	T	R	A	A	F	P	A	-900.973

**'090105060101010502**	Y	T	H	T	G	A	W	P	C	-900.968
**'040108060101030102**	I	T	S	T	G	A	Y	A	C	-900.962
**'040205070101010105**	I	A	H	V	G	A	W	A	V	-900.925
**'040109070101010102**	I	T	T	V	G	A	W	A	C	-900.923
**'040103020101020102**	I	T	D	I	G	A	F	A	C	-900.917
**'010103040201030501**	L	T	D	M	A	A	Y	P	A	-900.915
**'040309020101010105**	I	M	T	I	G	A	W	A	V	-900.903
**'040108020101020501**	I	T	S	I	G	A	F	P	A	-900.882
**'040204050101010102**	I	A	E	Q	G	A	W	A	C	-900.851
**'040107050101030501**	I	T	Q	Q	G	A	Y	P	A	-900.82
**'040107020101020105**	I	T	Q	I	G	A	F	A	V	-900.795
**'040105070101020501**	I	T	H	V	G	A	F	P	A	-900.792
**'010103020101020105**	L	T	D	I	G	A	F	A	V	-900.786
**'040304070101010501**	I	M	E	V	G	A	W	P	A	-900.78
**'040106070101030501**	I	T	N	V	G	A	Y	P	A	-900.718
**'040209070101010501**	I	A	T	V	G	A	W	P	A	-900.707
**'040104040101010501**	I	T	E	M	G	A	W	P	A	-900.7
**'040305050101010501**	I	M	H	Q	G	A	W	P	A	-900.694
**'010103020201030105**	L	T	D	I	A	A	Y	A	V	-900.681
**'070108010101020502**	V	T	S	R	G	A	F	P	C	-900.658
**'090105010101030501**	Y	T	H	R	G	A	Y	P	A	-900.648
**'040105020101010805**	I	T	H	I	G	A	W	S	V	-900.647
**'040303020101010501**	I	M	D	I	G	A	W	P	A	-900.59
**'070109040101020102**	V	T	T	M	G	A	F	A	C	-900.584
**'070109020101030501**	V	T	T	I	G	A	Y	P	A	-900.571
**'040105050101010305**	I	T	H	Q	G	A	W	G	V	-900.558
**'070109070101020501**	V	T	T	V	G	A	F	P	A	-900.549
**'040307070101030101**	I	M	Q	V	G	A	Y	A	A	-900.503
**'040109010101030802**	I	T	T	R	G	A	Y	S	C	-900.487
**'040105050101030102**	I	T	H	Q	G	A	Y	A	C	-900.444
**'040206010101010505**	I	A	N	R	G	A	W	P	V	-900.407
**'040302070101010101**	I	M	A	V	G	A	W	A	A	-900.391

**'070305070101010105**	V	M	H	V	G	A	W	A	V	-900.382
**'040107030201030101**	I	T	Q	K	A	A	Y	A	A	-900.377
**'070105020101030102**	V	T	H	I	G	A	Y	A	C	-900.371
**'020105040101030501**	F	T	H	M	G	A	Y	P	A	-900.368
**'070103040101030501**	V	T	D	M	G	A	Y	P	A	-900.365
**'040106060101010102**	I	T	N	T	G	A	W	A	C	-900.36
**'040109030101030105**	I	T	T	K	G	A	Y	A	V	-900.34
**'040104010101030502**	I	T	E	R	G	A	Y	P	C	-900.339
**'090205060101020101**	Y	A	H	T	G	A	F	A	A	-900.307
**'040105010101030202**	I	T	H	R	G	A	Y	E	C	-900.304
**'040105020101010501**	I	T	H	I	G	A	W	P	A	-900.3
**'070105070101020102**	V	T	H	V	G	A	F	A	C	-900.289
**'040305010101010505**	I	M	H	R	G	A	W	P	V	-900.276
**'040106040101010502**	I	T	N	M	G	A	W	P	C	-900.264
**'010105060101030502**	L	T	H	T	G	A	Y	P	C	-900.263
**'040307020101010102**	I	M	Q	I	G	A	W	A	C	-900.262
**'040207020101010501**	I	A	Q	I	G	A	W	P	A	-900.253
**'040203010101020105**	I	A	D	R	G	A	F	A	V	-900.209
**'040305070101010102**	I	M	H	V	G	A	W	A	C	-900.205
**'070107070101030105**	V	T	Q	V	G	A	Y	A	V	-900.197
**'070104050101030501**	V	T	E	Q	G	A	Y	P	A	-900.164
**'040205060101010501**	I	A	H	T	G	A	W	P	A	-900.151
**'040107060101010505**	I	T	Q	T	G	A	W	P	V	-900.147
**'040102030101020102**	I	T	A	K	G	A	F	A	C	-900.137
**'040105010201020501**	I	T	H	R	A	A	F	P	A	-900.133
**'070102020101020102**	V	T	A	I	G	A	F	A	C	-900.132
**'070102040101030102**	V	T	A	M	G	A	Y	A	C	-900.128
**'040304050101030101**	I	M	E	Q	G	A	Y	A	A	-900.099
**'040302040101010501**	I	M	A	M	G	A	W	P	A	-900.091
**'070207010101010502**	V	A	Q	R	G	A	W	P	C	-900.088
**'040305030101010105**	I	M	H	K	G	A	W	A	V	-900.086
**'040105010101020502**	I	T	H	R	G	A	F	P	C	-900.062

**'020107030101020501**	F	T	Q	K	G	A	F	P	A	-900.054
**'040205060101030101**	I	A	H	T	G	A	Y	A	A	-900.053
**'070105070101010505**	V	T	H	V	G	A	W	P	V	-900.046
**'070107020101010505**	V	T	Q	I	G	A	W	P	V	-900.041
**'040103070101020105**	I	T	D	V	G	A	F	A	V	-900.033
**'040309060101010201**	I	M	T	T	G	A	W	E	A	-900.031
**'040105060101010402**	I	T	H	T	G	A	W	K	C	-900.025
**'040102040201020101**	I	T	A	M	A	A	F	A	A	-900.015
**'070109060101010505**	V	T	T	T	G	A	W	P	V	-900.007
**'040105070101010502**	I	T	H	V	G	A	W	P	C	-899.998
**'040107030101010102**	I	T	Q	K	G	A	W	A	C	-899.991
**'040307010101020501**	I	M	Q	R	G	A	F	P	A	-899.981
**'010205040101030105**	L	A	H	M	G	A	Y	A	V	-899.972
**'040105060201030101**	I	T	H	T	A	A	Y	A	A	-899.968
**'040107020101030401**	I	T	Q	I	G	A	Y	K	A	-899.962
**'070107010101030502**	V	T	Q	R	G	A	Y	P	C	-899.958
**'070109010201020105**	V	T	T	R	A	A	F	A	V	-899.946
**'040208020101010102**	I	A	S	I	G	A	W	A	C	-899.946
**'090105020101030401**	Y	T	H	I	G	A	Y	K	A	-899.936
**'040302020101020101**	I	M	A	I	G	A	F	A	A	-899.934
**'040104050101010105**	I	T	E	Q	G	A	W	A	V	-899.931
**'010103060201020102**	L	T	D	T	A	A	F	A	C	-899.926
**'040105010201030401**	I	T	H	R	A	A	Y	K	A	-899.917
**'040308060101010501**	I	M	S	T	G	A	W	P	A	-899.916
**'040104020101030501**	I	T	E	I	G	A	Y	P	A	-899.913
**'070309070101010501**	V	M	T	V	G	A	W	P	A	-899.901
**'010103020101010505**	L	T	D	I	G	A	W	P	V	-899.899
**'010103020101020501**	L	T	D	I	G	A	F	P	A	-899.891
**'040107020201020101**	I	T	Q	I	A	A	F	A	A	-899.881
**'040109040101020401**	I	T	T	M	G	A	F	K	A	-899.874
**'020103020101010502**	F	T	D	I	G	A	W	P	C	-899.874
**'010105020101020305**	L	T	H	I	G	A	F	G	V	-899.873

'040103020101030105	I	T	D	I	G	A	Y	A	V	-899.865
'040209010101010205	I	A	T	R	G	A	W	E	V	-899.86
'040106050101020501	I	T	N	Q	G	A	F	P	A	-899.839
'010103070101030505	L	T	D	V	G	A	Y	P	V	-899.827
'070107020201030101	V	T	Q	I	A	A	Y	A	A	-899.82
'010104040101030501	L	T	E	M	G	A	Y	P	A	-899.82
'010109070101020505	L	T	T	V	G	A	F	P	V	-899.817
'040307040101010101	I	M	Q	M	G	A	W	A	A	-899.816
'040309040201010101	I	M	T	M	A	A	W	A	A	-899.79
'070205040101030101	V	A	H	M	G	A	Y	A	A	-899.784
'040207010101020401	I	A	Q	R	G	A	F	K	A	-899.771
'040209010101020102	I	A	T	R	G	A	F	A	C	-899.755
'070104020101010502	V	T	E	I	G	A	W	P	C	-899.752
'040107010101030102	I	T	Q	R	G	A	Y	A	C	-899.743
'010103060201030201	L	T	D	T	A	A	Y	E	A	-899.737
'040305040101030101	I	M	H	M	G	A	Y	A	A	-899.736
'010203020101010205	L	A	D	I	G	A	W	E	V	-899.727
'040107030101010205	I	T	Q	K	G	A	W	E	V	-899.713
'090105020101010505	Y	T	H	I	G	A	W	P	V	-899.711
'040208040101010105	I	A	S	M	G	A	W	A	V	-899.709
'070107060101030501	V	T	Q	T	G	A	Y	P	A	-899.708
'090105040201030101	Y	T	H	M	A	A	Y	A	A	-899.698
'010103060101030202	L	T	D	T	G	A	Y	E	C	-899.688
'010107040101020202	L	T	Q	M	G	A	F	E	C	-899.687
'040105020101020401	I	T	H	I	G	A	F	K	A	-899.67
'040102050201030101	I	T	A	Q	A	A	Y	A	A	-899.664
'010107070101030501	L	T	Q	V	G	A	Y	P	A	-899.663
'040109010101020505	I	T	T	R	G	A	F	P	V	-899.659
'040405010101020105	I	S	H	R	G	A	F	A	V	-899.653
'070207020101010102	V	A	Q	I	G	A	W	A	C	-899.652
'090105060201020101	Y	T	H	T	A	A	F	A	A	-899.646
'070109010101030102	V	T	T	R	G	A	Y	A	C	-899.646

**'040103020101010605**	I	T	D	I	G	A	W	Q	V	-899.646
**'070104010101020105**	V	T	E	R	G	A	F	A	V	-899.64
**'040305010101010202**	I	M	H	R	G	A	W	E	C	-899.632
**'070104010201030102**	V	T	E	R	A	A	Y	A	C	-899.624
**'010103040101020502**	L	T	D	M	G	A	F	P	C	-899.623
**'040104010101020202**	I	T	E	R	G	A	F	E	C	-899.609
**'050109070101010305**	M	T	T	V	G	A	W	G	V	-899.603
**'070105020101020105**	V	T	H	I	G	A	F	A	V	-899.593
**'040104030101020501**	I	T	E	K	G	A	F	P	A	-899.593
**'040109060201020101**	I	T	T	T	A	A	F	A	A	-899.583
**'040205010101030105**	I	A	H	R	G	A	Y	A	V	-899.566
**'040103010101030205**	I	T	D	R	G	A	Y	E	V	-899.562
**'040307010101010105**	I	M	Q	R	G	A	W	A	V	-899.555
**'020103030101030501**	F	T	D	K	G	A	Y	P	A	-899.554
**'040107070101010105**	I	T	Q	V	G	A	W	A	V	-899.545
**'040109020101010702**	I	T	T	I	G	A	W	R	C	-899.536
**'040405020101030101**	I	S	H	I	G	A	Y	A	A	-899.531
**'040106070101030102**	I	T	N	V	G	A	Y	A	C	-899.524
**'040303060101020101**	I	M	D	T	G	A	F	A	A	-899.511
**'020105060101020501**	F	T	H	T	G	A	F	P	A	-899.507
**'040107010201030201**	I	T	Q	R	A	A	Y	E	A	-899.504
**'010205020101010505**	L	A	H	I	G	A	W	P	V	-899.492
**'070104060101020501**	V	T	E	T	G	A	F	P	A	-899.488
**'040104020101010205**	I	T	E	I	G	A	W	E	V	-899.488
**'070102020101020501**	V	T	A	I	G	A	F	P	A	-899.48
**'040309030101010501**	I	M	T	K	G	A	W	P	A	-899.476
**'040205040101010102**	I	A	H	M	G	A	W	A	C	-899.475
**'040103010201030105**	I	T	D	R	A	A	Y	A	V	-899.461
**'040406070101010501**	I	S	N	V	G	A	W	P	A	-899.458
**'040403020101010105**	I	S	D	I	G	A	W	A	V	-899.439
**'040309070101020101**	I	M	T	V	G	A	F	A	A	-899.428
**'070108060101020105**	V	T	S	T	G	A	F	A	V	-899.424

**'070107050101010501**	V	T	Q	Q	G	A	W	P	A	-899.412
**'040405060101010102**	I	S	H	T	G	A	W	A	C	-899.41
**'040306040101020101**	I	M	N	M	G	A	F	A	A	-899.409
**'040104040101010102**	I	T	E	M	G	A	W	A	C	-899.405
**'040105040101010302**	I	T	H	M	G	A	W	G	C	-899.39
**'070107010201020102**	V	T	Q	R	A	A	F	A	C	-899.386
**'040103060101010405**	I	T	D	T	G	A	W	K	V	-899.384
**'040109010101010202**	I	T	T	R	G	A	W	E	C	-899.381
**'040105070101030401**	I	T	H	V	G	A	Y	K	A	-899.38
**'040107070101010302**	I	T	Q	V	G	A	W	G	C	-899.355
**'010103070201020501**	L	T	D	V	A	A	F	P	A	-899.354
**'040103050101010502**	I	T	D	Q	G	A	W	P	C	-899.353
**'040308070101010105**	I	M	S	V	G	A	W	A	V	-899.35
**'020103070101020501**	F	T	D	V	G	A	F	P	A	-899.344
**'050109040101010502**	M	T	T	M	G	A	W	P	C	-899.344
**'070105010101030305**	V	T	H	R	G	A	Y	G	V	-899.341
**'040107050101020701**	I	T	Q	Q	G	A	F	R	A	-899.34
**'010403060101020501**	L	S	D	T	G	A	F	P	A	-899.336
**'010103010201030502**	L	T	D	R	A	A	Y	P	C	-899.333
**'050109040101030105**	M	T	T	M	G	A	Y	A	V	-899.331
**'070103030101010505**	V	T	D	K	G	A	W	P	V	-899.312
**'010108040101020505**	L	T	S	M	G	A	F	P	V	-899.306
**'070105010201030105**	V	T	H	R	A	A	Y	A	V	-899.292
**'090109060101030501**	Y	T	T	T	G	A	Y	P	A	-899.291
**'040107070101010501**	I	T	Q	V	G	A	W	P	A	-899.287
**'070206060101010105**	V	A	N	T	G	A	W	A	V	-899.279
**'090105010101030105**	Y	T	H	R	G	A	Y	A	V	-899.278
**'040103040101010202**	I	T	D	M	G	A	W	E	C	-899.273
**'050108070101010505**	M	T	S	V	G	A	W	P	V	-899.262
**'040104060101020105**	I	T	E	T	G	A	F	A	V	-899.251
**'090105070101030701**	Y	T	H	V	G	A	Y	R	A	-899.251
**'040305020101010101**	I	M	H	I	G	A	W	A	A	-899.25

**'010103010201020505**	L	T	D	R	A	A	F	P	V	-899.243
**'040109070101010503**	I	T	T	V	G	A	W	P	S	-899.239
**'040306010101030501**	I	M	N	R	G	A	Y	P	A	-899.238
**'070109020101010605**	V	T	T	I	G	A	W	Q	V	-899.236
**'040108020201030101**	I	T	S	I	A	A	Y	A	A	-899.232
**'040108040101010205**	I	T	S	M	G	A	W	E	V	-899.23
**'040107040101010402**	I	T	Q	M	G	A	W	K	C	-899.23
**'070103070101030102**	V	T	D	V	G	A	Y	A	C	-899.22
**'040108040101030105**	I	T	S	M	G	A	Y	A	V	-899.204
**'040109040201010501**	I	T	T	M	A	A	W	P	A	-899.197
**'070105060101010302**	V	T	H	T	G	A	W	G	C	-899.194
**'040302010101020105**	I	M	A	R	G	A	F	A	V	-899.191
**'010405070101020105**	L	S	H	V	G	A	F	A	V	-899.188
**'070107010201030501**	V	T	Q	R	A	A	Y	P	A	-899.172
**'010403060101010502**	L	S	D	T	G	A	W	P	C	-899.171
**'010103070101020102**	L	T	D	V	G	A	F	A	C	-899.164
**'010105020101020502**	L	T	H	I	G	A	F	P	C	-899.159
**'040107020101010101**	I	T	Q	I	G	A	W	A	A	-899.147
**'070109040101010202**	V	T	T	M	G	A	W	E	C	-899.143
**'040302010101010502**	I	M	A	R	G	A	W	P	C	-899.135
**'070305060101010101**	V	M	H	T	G	A	W	A	A	-899.133
**'010105040101020605**	L	T	H	M	G	A	F	Q	V	-899.131
**'050104040101020105**	M	T	E	M	G	A	F	A	V	-899.129
**'090105040101010205**	Y	T	H	M	G	A	W	E	V	-899.125
**'040102020101010305**	I	T	A	I	G	A	W	G	V	-899.121
**'010103070101030602**	L	T	D	V	G	A	Y	Q	C	-899.115
**'040107040101020103**	I	T	Q	M	G	A	F	A	S	-899.105
**'020105010101030502**	F	T	H	R	G	A	Y	P	C	-899.104
**'040204050101010501**	I	A	E	Q	G	A	W	P	A	-899.102
**'040106070101010802**	I	T	N	V	G	A	W	S	C	-899.075
**'040207020101010105**	I	A	Q	I	G	A	W	A	V	-899.071
**'070106050101030105**	V	T	N	Q	G	A	Y	A	V	-899.066

**'090105040101020201**	Y	T	H	M	G	A	F	E	A	-899.061
**'070305070101030101**	V	M	H	V	G	A	Y	A	A	-899.054
**'070105050101010405**	V	T	H	Q	G	A	W	K	V	-899.049
**'010203060101030501**	L	A	D	T	G	A	Y	P	A	-899.04
**'010209070101030105**	L	A	T	V	G	A	Y	A	V	-899.027
**'050108020101010502**	M	T	S	I	G	A	W	P	C	-899.026
**'040205010101020501**	I	A	H	R	G	A	F	P	A	-899.02
**'020103050201030101**	F	T	D	Q	A	A	Y	A	A	-899.015
**'040104050101010202**	I	T	E	Q	G	A	W	E	C	-899.015
**'090205040101010501**	Y	A	H	M	G	A	W	P	A	-899.004
**'040107040101010805**	I	T	Q	M	G	A	W	S	V	-899.004
**'040205040101020101**	I	A	H	M	G	A	F	A	A	-898.985
**'040108070101020102**	I	T	S	V	G	A	F	A	C	-898.984
**'070307020101010501**	V	M	Q	I	G	A	W	P	A	-898.984
**'010403040101030102**	L	S	D	M	G	A	Y	A	C	-898.984
**'040209010101030501**	I	A	T	R	G	A	Y	P	A	-898.983
**'040108010101030705**	I	T	S	R	G	A	Y	R	V	-898.977
**'070108040101010502**	V	T	S	M	G	A	W	P	C	-898.968
**'010203060101030105**	L	A	D	T	G	A	Y	A	V	-898.968
**'070108070101030501**	V	T	S	V	G	A	Y	P	A	-898.967
**'070107060101010202**	V	T	Q	T	G	A	W	E	C	-898.966
**'020203040101030101**	F	A	D	M	G	A	Y	A	A	-898.959
**'040105070101010205**	I	T	H	V	G	A	W	E	V	-898.959
**'090109020101010501**	Y	T	T	I	G	A	W	P	A	-898.955
**'010203070101010502**	L	A	D	V	G	A	W	P	C	-898.952
**'010403050101010505**	L	S	D	Q	G	A	W	P	V	-898.952
**'070105030101010105**	V	T	H	K	G	A	W	A	V	-898.951
**'040103010101020501**	I	T	D	R	G	A	F	P	A	-898.945
**'070102050101010502**	V	T	A	Q	G	A	W	P	C	-898.941
**'040109060101030101**	I	T	T	T	G	A	Y	A	A	-898.928
**'090305040101010105**	Y	M	H	M	G	A	W	A	V	-898.928
**'070104030101030102**	V	T	E	K	G	A	Y	A	C	-898.926

**'010403060101020102**	L	S	D	T	G	A	F	A	C	-898.901
**'010208060101020105**	L	A	S	T	G	A	F	A	V	-898.901
**'070106060101030102**	V	T	N	T	G	A	Y	A	C	-898.899
**'040102040101010705**	I	T	A	M	G	A	W	R	V	-898.895
**'040102040201010102**	I	T	A	M	A	A	W	A	C	-898.886
**'040104010101030805**	I	T	E	R	G	A	Y	S	V	-898.885
**'040107020101010705**	I	T	Q	I	G	A	W	R	V	-898.875
**'070103020101010105**	V	T	D	I	G	A	W	A	V	-898.874
**'090105020101010602**	Y	T	H	I	G	A	W	Q	C	-898.874
**'070108040101020501**	V	T	S	M	G	A	F	P	A	-898.872
**'090103020101030501**	Y	T	D	I	G	A	Y	P	A	-898.871
**'070105030101010502**	V	T	H	K	G	A	W	P	C	-898.862
**'040309010201020101**	I	M	T	R	A	A	F	A	A	-898.857
**'050108070101020105**	M	T	S	V	G	A	F	A	V	-898.855
**'070105070101010702**	V	T	H	V	G	A	W	R	C	-898.853
**'010105060101020405**	L	T	H	T	G	A	F	K	V	-898.844
**'090105010101020705**	Y	T	H	R	G	A	F	R	V	-898.834
**'090105040101010102**	Y	T	H	M	G	A	W	A	C	-898.833
**'070405010101010105**	V	S	H	R	G	A	W	A	V	-898.832
**'010303050101010502**	L	M	D	Q	G	A	W	P	C	-898.823
**'040103020101030701**	I	T	D	I	G	A	Y	R	A	-898.82
**'040304060101010102**	I	M	E	T	G	A	W	A	C	-898.815
**'070207010201030101**	V	A	Q	R	A	A	Y	A	A	-898.812
**'070209060101010501**	V	A	T	T	G	A	W	P	A	-898.81
**'040305070101010401**	I	M	H	V	G	A	W	K	A	-898.801
**'070109010101020305**	V	T	T	R	G	A	F	G	V	-898.8
**'070107040201020101**	V	T	Q	M	A	A	F	A	A	-898.788
**'010105060101010305**	L	T	H	T	G	A	W	G	V	-898.786
**'040103040201030101**	I	T	D	M	A	A	Y	A	A	-898.778
**'010403010101030105**	L	S	D	R	G	A	Y	A	V	-898.777
**'010203070101020105**	L	A	D	V	G	A	F	A	V	-898.772
**'040109070201030101**	I	T	T	V	A	A	Y	A	A	-898.768

**'070102010101030505**	V	T	A	R	G	A	Y	P	V	-898.758
**'090109010201030501**	Y	T	T	R	A	A	Y	P	A	-898.755
**'050107020101030105**	M	T	Q	I	G	A	Y	A	V	-898.754
**'040409050101010102**	I	S	T	Q	G	A	W	A	C	-898.752
**'010107020201020105**	L	T	Q	I	A	A	F	A	V	-898.743
**'040109050101030401**	I	T	T	Q	G	A	Y	K	A	-898.742
**'040106010101030105**	I	T	N	R	G	A	Y	A	V	-898.74
**'040108030101010505**	I	T	S	K	G	A	W	P	V	-898.74
**'070105020101010802**	V	T	H	I	G	A	W	S	C	-898.736
**'070407040101010501**	V	S	Q	M	G	A	W	P	A	-898.735
**'040305040101010103**	I	M	H	M	G	A	W	A	S	-898.734
**'070202020101010501**	V	A	A	I	G	A	W	P	A	-898.73
**'040304010201030101**	I	M	E	R	A	A	Y	A	A	-898.729
**'040108030101030501**	I	T	S	K	G	A	Y	P	A	-898.727
**'040308020101010201**	I	M	S	I	G	A	W	E	A	-898.714
**'010205020101020105**	L	A	H	I	G	A	F	A	V	-898.712
**'010403020101030501**	L	S	D	I	G	A	Y	P	A	-898.71
**'040405010101030102**	I	S	H	R	G	A	Y	A	C	-898.698
**'090105060101020105**	Y	T	H	T	G	A	F	A	V	-898.695
**'010203040101010102**	L	A	D	M	G	A	W	A	C	-898.689
**'040307010101030601**	I	M	Q	R	G	A	Y	Q	A	-898.685
**'040106040101020102**	I	T	N	M	G	A	F	A	C	-898.681
**'070104010101030205**	V	T	E	R	G	A	Y	E	V	-898.664
**'010107040101030502**	L	T	Q	M	G	A	Y	P	C	-898.656
**'010103070201030102**	L	T	D	V	A	A	Y	A	C	-898.655
**'090105070101010202**	Y	T	H	V	G	A	W	E	C	-898.649
**'010103060101020402**	L	T	D	T	G	A	F	K	C	-898.647
**'070109060101010402**	V	T	T	T	G	A	W	K	C	-898.645
**'010109020101030502**	L	T	T	I	G	A	Y	P	C	-898.637
**'010104020101020102**	L	T	E	I	G	A	F	A	C	-898.637
**'040108070101010702**	I	T	S	V	G	A	W	R	C	-898.635
**'010205010101020505**	L	A	H	R	G	A	F	P	V	-898.635

**'040309040101010401**	I	M	T	M	G	A	W	K	A	-898.633
**'070104020101030201**	V	T	E	I	G	A	Y	E	A	-898.631
**'010209020101010502**	L	A	T	I	G	A	W	P	C	-898.629
**'070105020101010705**	V	T	H	I	G	A	W	R	V	-898.625
**'070107010101010102**	V	T	Q	R	G	A	W	A	C	-898.621
**'020103020101010305**	F	T	D	I	G	A	W	G	V	-898.619
**'090102040101030501**	Y	T	A	M	G	A	Y	P	A	-898.602
**'010105060201020105**	L	T	H	T	A	A	F	A	V	-898.593
**'010303070201020101**	L	M	D	V	A	A	F	A	A	-898.593
**'020403060101010501**	F	S	D	T	G	A	W	P	A	-898.585
**'090107070101020501**	Y	T	Q	V	G	A	F	P	A	-898.582
**'020103040101030102**	F	T	D	M	G	A	Y	A	C	-898.568
**'070307060101010105**	V	M	Q	T	G	A	W	A	V	-898.562
**'040104060101010701**	I	T	E	T	G	A	W	R	A	-898.562
**'040107050101010702**	I	T	Q	Q	G	A	W	R	C	-898.561
**'040106050101010505**	I	T	N	Q	G	A	W	P	V	-898.558
**'070203070101010105**	V	A	D	V	G	A	W	A	V	-898.556
**'070109030101020105**	V	T	T	K	G	A	F	A	V	-898.553
**'070302050101010105**	V	M	A	Q	G	A	W	A	V	-898.541
**'040302030101010201**	I	M	A	K	G	A	W	E	A	-898.54
**'040107020101020201**	I	T	Q	I	G	A	F	E	A	-898.537
**'040309010101010102**	I	M	T	R	G	A	W	A	C	-898.536
**'040206020101010401**	I	A	N	I	G	A	W	K	A	-898.534
**'040105010101010105**	I	T	H	R	G	A	W	A	V	-898.528
**'010102010201020502**	L	T	A	R	A	A	F	P	C	-898.526
**'010203010201030501**	L	A	D	R	A	A	Y	P	A	-898.523
**'050104060101010505**	M	T	E	T	G	A	W	P	V	-898.522
**'040102040101010602**	I	T	A	M	G	A	W	Q	C	-898.519
**'010105050101030505**	L	T	H	Q	G	A	Y	P	V	-898.495
**'050107020101020501**	M	T	Q	I	G	A	F	P	A	-898.494
**'050109050101020105**	M	T	T	Q	G	A	F	A	V	-898.487
**'040306030101030101**	I	M	N	K	G	A	Y	A	A	-898.486

**'040104060101010602**	I	T	E	T	G	A	W	Q	C	-898.482
**'010208020101030105**	L	A	S	I	G	A	Y	A	V	-898.478
**'040307070101010201**	I	M	Q	V	G	A	W	E	A	-898.475
**'050109010101020102**	M	T	T	R	G	A	F	A	C	-898.469
**'040205010201020101**	I	A	H	R	A	A	F	A	A	-898.469
**'090405060101010501**	Y	S	H	T	G	A	W	P	A	-898.468
**'040107070101020101**	I	T	Q	V	G	A	F	A	A	-898.468
**'040109010201020102**	I	T	T	R	A	A	F	A	C	-898.467
**'090203070101010501**	Y	A	D	V	G	A	W	P	A	-898.467
**'010103070101020705**	L	T	D	V	G	A	F	R	V	-898.465
**'010105020101030105**	L	T	H	I	G	A	Y	A	V	-898.461
**'070102040101010501**	V	T	A	M	G	A	W	P	A	-898.459
**'040109040101010605**	I	T	T	M	G	A	W	Q	V	-898.458
**'010103060101020505**	L	T	D	T	G	A	F	P	V	-898.456
**'040105050101030201**	I	T	H	Q	G	A	Y	E	A	-898.456
**'050108040201020101**	M	T	S	M	A	A	F	A	A	-898.455
**'040104060101030401**	I	T	E	T	G	A	Y	K	A	-898.453
**'010206020101030102**	L	A	N	I	G	A	Y	A	C	-898.448
**'010109060201030105**	L	T	T	T	A	A	Y	A	V	-898.446
**'050107040101020102**	M	T	Q	M	G	A	F	A	C	-898.441
**'010303060201030101**	L	M	D	T	A	A	Y	A	A	-898.44
**'070103010101020702**	V	T	D	R	G	A	F	R	C	-898.438
**'070306020101030101**	V	M	N	I	G	A	Y	A	A	-898.431
**'070102020201020101**	V	T	A	I	A	A	F	A	A	-898.422
**'090305010101020501**	Y	M	H	R	G	A	F	P	A	-898.415
**'070105060101030401**	V	T	H	T	G	A	Y	K	A	-898.414
**'070302060101030101**	V	M	A	T	G	A	Y	A	A	-898.414
**'040203010201030101**	I	A	D	R	A	A	Y	A	A	-898.413
**'070208070101010501**	V	A	S	V	G	A	W	P	A	-898.411
**'040104070201020101**	I	T	E	V	A	A	F	A	A	-898.409
**'040304010101030102**	I	M	E	R	G	A	Y	A	C	-898.4
**'070307020101020101**	V	M	Q	I	G	A	F	A	A	-898.399

**'090105030101020102**	Y	T	H	K	G	A	F	A	C	-898.395
**'050109020201030101**	M	T	T	I	A	A	Y	A	A	-898.391
**'090105070101010501**	Y	T	H	V	G	A	W	P	A	-898.387
**'040109010101020402**	I	T	T	R	G	A	F	K	C	-898.386
**'040409010101010105**	I	S	T	R	G	A	W	A	V	-898.384
**'040306010101020102**	I	M	N	R	G	A	F	A	C	-898.378
**'040209020101010103**	I	A	T	I	G	A	W	A	S	-898.376
**'040203040101010501**	I	A	D	M	G	A	W	P	A	-898.371
**'040105050101010602**	I	T	H	Q	G	A	W	Q	C	-898.368
**'050104060101030105**	M	T	E	T	G	A	Y	A	V	-898.364
**'040108010201030801**	I	T	S	R	A	A	Y	S	A	-898.362
**'070104040101010402**	V	T	E	M	G	A	W	K	C	-898.357
**'010303010101030502**	L	M	D	R	G	A	Y	P	C	-898.35
**'070101020101030505**	V	T	G	I	G	A	Y	P	V	-898.349
**'070107010101020202**	V	T	Q	R	G	A	F	E	C	-898.348
**'040209020101010701**	I	A	T	I	G	A	W	R	A	-898.347
**'070305010101010205**	V	M	H	R	G	A	W	E	V	-898.345
**'020303070101010501**	F	M	D	V	G	A	W	P	A	-898.343
**'040409040101010501**	I	S	T	M	G	A	W	P	A	-898.342
**'010203040201020101**	L	A	D	M	A	A	F	A	A	-898.342
**'070106020101020401**	V	T	N	I	G	A	F	K	A	-898.337
**'070109020101010102**	V	T	T	I	G	A	W	A	C	-898.337
**'020105060101030102**	F	T	H	T	G	A	Y	A	C	-898.336
**'040108020201010105**	I	T	S	I	A	A	W	A	V	-898.336
**'040105030101010405**	I	T	H	K	G	A	W	K	V	-898.335
**'010105010101020205**	L	T	H	R	G	A	F	E	V	-898.333
**'070104010101020802**	V	T	E	R	G	A	F	S	C	-898.331
**'070106040101010505**	V	T	N	M	G	A	W	P	V	-898.33
**'020109020101020102**	F	T	T	I	G	A	F	A	C	-898.326
**'040108010101030602**	I	T	S	R	G	A	Y	Q	C	-898.323
**'040103070101020201**	I	T	D	V	G	A	F	E	A	-898.322
**'090105010201030201**	Y	T	H	R	A	A	Y	E	A	-898.321

**'040209060101010102**	I	A	T	T	G	A	W	A	C	-898.32
**'070104060101010205**	V	T	E	T	G	A	W	E	V	-898.32
**'040107010101020405**	I	T	Q	R	G	A	F	K	V	-898.31
**'010104050201030501**	L	T	E	Q	A	A	Y	P	A	-898.306
**'050104010101010305**	M	T	E	R	G	A	W	G	V	-898.297
**'040101070101020502**	I	T	G	V	G	A	F	P	C	-898.296
**'040109020201010201**	I	T	T	I	A	A	W	E	A	-898.296
**'070104060101020102**	V	T	E	T	G	A	F	A	C	-898.294
**'070107010101020501**	V	T	Q	R	G	A	F	P	A	-898.294
**'090205070101030101**	Y	A	H	V	G	A	Y	A	A	-898.289
**'040209030101020101**	I	A	T	K	G	A	F	A	A	-898.288
**'070104020101020301**	V	T	E	I	G	A	F	G	A	-898.287
**'040106020101010105**	I	T	N	I	G	A	W	A	V	-898.285
**'010104040201020105**	L	T	E	M	A	A	F	A	V	-898.272
**'040102010101020602**	I	T	A	R	G	A	F	Q	C	-898.266
**'010209020101010105**	L	A	T	I	G	A	W	A	V	-898.26
**'010103050101030802**	L	T	D	Q	G	A	Y	S	C	-898.256
**'040105010201010505**	I	T	H	R	A	A	W	P	V	-898.255
**'040109020101020601**	I	T	T	I	G	A	F	Q	A	-898.253
**'040409010101020501**	I	S	T	R	G	A	F	P	A	-898.251
**'070205010101020105**	V	A	H	R	G	A	F	A	V	-898.251
**'070102030101030501**	V	T	A	K	G	A	Y	P	A	-898.25
**'040105060101010705**	I	T	H	T	G	A	W	R	V	-898.246
**'040104020101010802**	I	T	E	I	G	A	W	S	C	-898.243
**'040206030101010501**	I	A	N	K	G	A	W	P	A	-898.241
**'070107060101010605**	V	T	Q	T	G	A	W	Q	V	-898.234
**'040202070101010401**	I	A	A	V	G	A	W	K	A	-898.221
**'010103020101030702**	L	T	D	I	G	A	Y	R	C	-898.22
**'090209020101020101**	Y	A	T	I	G	A	F	A	A	-898.216
**'040108030101010202**	I	T	S	K	G	A	W	E	C	-898.214
**'050103030101020501**	M	T	D	K	G	A	F	P	A	-898.213
**'010103010101020805**	L	T	D	R	G	A	F	S	V	-898.211

**'090103060101030102**	Y	T	D	T	G	A	Y	A	C	-898.211
**'070109010101010502**	V	T	T	R	G	A	W	P	C	-898.211
**'010106070101020501**	L	T	N	V	G	A	F	P	A	-898.202
**'010105010201030102**	L	T	H	R	A	A	Y	A	C	-898.198
**'020103030101020701**	F	T	D	K	G	A	F	R	A	-898.196
**'070305040101010102**	V	M	H	M	G	A	W	A	C	-898.194
**'010103010101030605**	L	T	D	R	G	A	Y	Q	V	-898.189
**'040207040101010401**	I	A	Q	M	G	A	W	K	A	-898.189
**'010209010101020105**	L	A	T	R	G	A	F	A	V	-898.187
**'070105060101030103**	V	T	H	T	G	A	Y	A	S	-898.184
**'040107010201020105**	I	T	Q	R	A	A	F	A	V	-898.178
**'040404020101010102**	I	S	E	I	G	A	W	A	C	-898.174
**'070106020201010501**	V	T	N	I	A	A	W	P	A	-898.174
**'040104030101010605**	I	T	E	K	G	A	W	Q	V	-898.172
**'040207010101010605**	I	A	Q	R	G	A	W	Q	V	-898.172
**'070309070101010102**	V	M	T	V	G	A	W	A	C	-898.167
**'020103010201030401**	F	T	D	R	A	A	Y	K	A	-898.164
**'040308030101020101**	I	M	S	K	G	A	F	A	A	-898.164
**'030105060101010505**	H	T	H	T	G	A	W	P	V	-898.161
**'070102060101010105**	V	T	A	T	G	A	W	A	V	-898.153
**'040103010201020101**	I	T	D	R	A	A	F	A	A	-898.147
**'010202040101020105**	L	A	A	M	G	A	F	A	V	-898.14
**'070107010101030805**	V	T	Q	R	G	A	Y	S	V	-898.134
**'070302060101010501**	V	M	A	T	G	A	W	P	A	-898.133
**'040307010101010302**	I	M	Q	R	G	A	W	G	C	-898.132
**'070104040201010501**	V	T	E	M	A	A	W	P	A	-898.131
**'010104060201020501**	L	T	E	T	A	A	F	P	A	-898.131
**'040109070101030701**	I	T	T	V	G	A	Y	R	A	-898.115
**'070109010201030201**	V	T	T	R	A	A	Y	E	A	-898.114
**'040404010201020101**	I	S	E	R	A	A	F	A	A	-898.114
**'040106060101020401**	I	T	N	T	G	A	F	K	A	-898.113
**'090105060201010501**	Y	T	H	T	A	A	W	P	A	-898.109

'040106020101010701	I	T	N	I	G	A	W	R	A	-898.109
'090105010201020401	Y	T	H	R	A	A	F	K	A	-898.103
'040109040101010802	I	T	T	M	G	A	W	S	C	-898.101
'040106060101010501	I	T	N	T	G	A	W	P	A	-898.098
'010105020101010205	L	T	H	I	G	A	W	E	V	-898.09
'050104050101030102	M	T	E	Q	G	A	Y	A	C	-898.076
'010405050101030105	L	S	H	Q	G	A	Y	A	V	-898.075
'010103070101020503	L	T	D	V	G	A	F	P	S	-898.074
'010203040101020501	L	A	D	M	G	A	F	P	A	-898.07
'010303050101010205	L	M	D	Q	G	A	W	E	V	-898.063
'070104020101010305	V	T	E	I	G	A	W	G	V	-898.06
'070101040101030502	V	T	G	M	G	A	Y	P	C	-898.059
'040109050101010302	I	T	T	Q	G	A	W	G	C	-898.051
'090105030101020401	Y	T	H	K	G	A	F	K	A	-898.049
'010107040201020102	L	T	Q	M	A	A	F	A	C	-898.036
'040304010101020401	I	M	E	R	G	A	F	K	A	-898.034
'070102070101010405	V	T	A	V	G	A	W	K	V	-898.033
'050107070101010202	M	T	Q	V	G	A	W	E	C	-898.032
'040309010101030201	I	M	T	R	G	A	Y	E	A	-898.029
'070107030101010702	V	T	Q	K	G	A	W	R	C	-898.029
'040404010101030501	I	S	E	R	G	A	Y	P	A	-898.026
'010103040201010502	L	T	D	M	A	A	W	P	C	-898.019
'010107070201030105	L	T	Q	V	A	A	Y	A	V	-898.019
'040105010101020101	I	T	H	R	G	A	F	A	A	-898.017
'070308060101020101	V	M	S	T	G	A	F	A	A	-898.015
'010209060101010505	L	A	T	T	G	A	W	P	V	-898.013
'070207010101030501	V	A	Q	R	G	A	Y	P	A	-898.011
'070103050101020105	V	T	D	Q	G	A	F	A	V	-898.004
'090305020101010501	Y	M	H	I	G	A	W	P	A	-898.004
'040303010201010102	I	M	D	R	A	A	W	A	C	-898.002
'020403040101010401	F	S	D	M	G	A	W	K	A	-898.0
'010403060201020101	L	S	D	T	A	A	F	A	A	-897.999

**'040109070101010405**	I	T	T	V	G	A	W	K	V	-897.994
**'070106060101010502**	V	T	N	T	G	A	W	P	C	-897.99
**'010103060201010302**	L	T	D	T	A	A	W	G	C	-897.989
**'040106010101020805**	I	T	N	R	G	A	F	S	V	-897.988
**'040104030101010502**	I	T	E	K	G	A	W	P	C	-897.983
**'010109020201030102**	L	T	T	I	A	A	Y	A	C	-897.983
**'020104010101030501**	F	T	E	R	G	A	Y	P	A	-897.982
**'040105010101030503**	I	T	H	R	G	A	Y	P	S	-897.982
**'040107040201010801**	I	T	Q	M	A	A	W	S	A	-897.981
**'010203010201030105**	L	A	D	R	A	A	Y	A	V	-897.978
**'010109060101020305**	L	T	T	T	G	A	F	G	V	-897.977
**'090105060101010101**	Y	T	H	T	G	A	W	A	A	-897.969
**'040105040101030701**	I	T	H	M	G	A	Y	R	A	-897.969
**'010105040101010502**	L	T	H	M	G	A	W	P	C	-897.967
**'040105030101010802**	I	T	H	K	G	A	W	S	C	-897.966
**'010209040101010205**	L	A	T	M	G	A	W	E	V	-897.964
**'070108050101020102**	V	T	S	Q	G	A	F	A	C	-897.963
**'040107040201010105**	I	T	Q	M	A	A	W	A	V	-897.962
**'020103060101010505**	F	T	D	T	G	A	W	P	V	-897.96
**'050104070101020501**	M	T	E	V	G	A	F	P	A	-897.956
**'040104030201010501**	I	T	E	K	A	A	W	P	A	-897.949
**'050106040101030501**	M	T	N	M	G	A	Y	P	A	-897.947
**'070108010201030401**	V	T	S	R	A	A	Y	K	A	-897.941
**'040109050101020102**	I	T	T	Q	G	A	F	A	C	-897.936
**'040105040201010101**	I	T	H	M	A	A	W	A	A	-897.927
**'010103020101020802**	L	T	D	I	G	A	F	S	C	-897.926
**'040105010101030605**	I	T	H	R	G	A	Y	Q	V	-897.926
**'010105010101030405**	L	T	H	R	G	A	Y	K	V	-897.924
**'040105060101030801**	I	T	H	T	G	A	Y	S	A	-897.918
**'040305040101010601**	I	M	H	M	G	A	W	Q	A	-897.917
**'040405010101010305**	I	S	H	R	G	A	W	G	V	-897.915
**'040106020101030101**	I	T	N	I	G	A	Y	A	A	-897.913

'050109020101020701	M	T	T	I	G	A	F	R	A	-897.913
'070105060101010501	V	T	H	T	G	A	W	P	A	-897.912
'050108010101020505	M	T	S	R	G	A	F	P	V	-897.91
'040203010101010202	I	A	D	R	G	A	W	E	C	-897.907
'070105010201010305	V	T	H	R	A	A	W	G	V	-897.899
'040306070201010101	I	M	N	V	A	A	W	A	A	-897.896
'070106040101010102	V	T	N	M	G	A	W	A	C	-897.896
'040303050101010401	I	M	D	Q	G	A	W	K	A	-897.895
'040103040101010105	I	T	D	M	G	A	W	A	V	-897.894
'090105070101030102	Y	T	H	V	G	A	Y	A	C	-897.887
'010303030101030105	L	M	D	K	G	A	Y	A	V	-897.886
'070104050101010605	V	T	E	Q	G	A	W	Q	V	-897.886
'010404070101030501	L	S	E	V	G	A	Y	P	A	-897.885
'030109010101030505	H	T	T	R	G	A	Y	P	V	-897.883
'070303010101010505	V	M	D	R	G	A	W	P	V	-897.883
'010105040201020501	L	T	H	M	A	A	F	P	A	-897.883
'040109030201010105	I	T	T	K	A	A	W	A	V	-897.879
'070307040101010201	V	M	Q	M	G	A	W	E	A	-897.874
'040308040101010102	I	M	S	M	G	A	W	A	C	-897.873
'040302060101010801	I	M	A	T	G	A	W	S	A	-897.87
'090103070201030101	Y	T	D	V	A	A	Y	A	A	-897.87
'020103060101020105	F	T	D	T	G	A	F	A	V	-897.868
'090105060101010405	Y	T	H	T	G	A	W	K	V	-897.867
'010205060101010205	L	A	H	T	G	A	W	E	V	-897.865
'010303020101030102	L	M	D	I	G	A	Y	A	C	-897.861
'010103070201030701	L	T	D	V	A	A	Y	R	A	-897.861
'090105010101030802	Y	T	H	R	G	A	Y	S	C	-897.856
'010303020101010805	L	M	D	I	G	A	W	S	V	-897.856
'010205060101020501	L	A	H	T	G	A	F	P	A	-897.855
'070106040201030101	V	T	N	M	A	A	Y	A	A	-897.85
'070309040101010105	V	M	T	M	G	A	W	A	V	-897.85
'050102040101010505	M	T	A	M	G	A	W	P	V	-897.847

**'010107020201020501**	L	T	Q	I	A	A	F	P	A	-897.846
**'070107020101010302**	V	T	Q	I	G	A	W	G	C	-897.841
**'070108060101010305**	V	T	S	T	G	A	W	G	V	-897.841
**'040102020201010501**	I	T	A	I	A	A	W	P	A	-897.839
**'010105010201020202**	L	T	H	R	A	A	F	E	C	-897.833
**'040107050201010501**	I	T	Q	Q	A	A	W	P	A	-897.829
**'040105070201010102**	I	T	H	V	A	A	W	A	C	-897.828
**'010103050201020105**	L	T	D	Q	A	A	F	A	V	-897.827
**'010207050101020105**	L	A	Q	Q	G	A	F	A	V	-897.826
**'050106060101010305**	M	T	N	T	G	A	W	G	V	-897.825
**'040107010201020401**	I	T	Q	R	A	A	F	K	A	-897.823
**'040108060101010401**	I	T	S	T	G	A	W	K	A	-897.816
**'040407010101010502**	I	S	Q	R	G	A	W	P	C	-897.816
**'030104040101010502**	H	T	E	M	G	A	W	P	C	-897.809
**'070104030101020401**	V	T	E	K	G	A	F	K	A	-897.804
**'070102020101010402**	V	T	A	I	G	A	W	K	C	-897.801
**'070104010201020801**	V	T	E	R	A	A	F	S	A	-897.799
**'020107020101030501**	F	T	Q	I	G	A	Y	P	A	-897.797
**'010103030101020202**	L	T	D	K	G	A	F	E	C	-897.794
**'040104070201010105**	I	T	E	V	A	A	W	A	V	-897.792
**'040308030201010101**	I	M	S	K	A	A	W	A	A	-897.785
**'070106020101010205**	V	T	N	I	G	A	W	E	V	-897.784
**'010104050101020501**	L	T	E	Q	G	A	F	P	A	-897.779
**'040309020101010801**	I	M	T	I	G	A	W	S	A	-897.775
**'020105070101010302**	F	T	H	V	G	A	W	G	C	-897.775
**'070306040101010501**	V	M	N	M	G	A	W	P	A	-897.774
**'040104040101010405**	I	T	E	M	G	A	W	K	V	-897.771
**'040307020101010401**	I	M	Q	I	G	A	W	K	A	-897.77
**'020103040101010501**	F	T	D	M	G	A	W	P	A	-897.77
**'010205010101030502**	L	A	H	R	G	A	Y	P	C	-897.766
**'070107060101020201**	V	T	Q	T	G	A	F	E	A	-897.765
**'010205070101030102**	L	A	H	V	G	A	Y	A	C	-897.764

'070102070101020105	V	T	A	V	G	A	F	A	V	-897.762
'070107060101010503	V	T	Q	T	G	A	W	P	S	-897.757
'010104070101030502	L	T	E	V	G	A	Y	P	C	-897.756
'070104010101030402	V	T	E	R	G	A	Y	K	C	-897.756
'070409010101020102	V	S	T	R	G	A	F	A	C	-897.756
'070106030101020501	V	T	N	K	G	A	F	P	A	-897.754
'040107060101010303	I	T	Q	T	G	A	W	G	S	-897.752
'050109040101030401	M	T	T	M	G	A	Y	K	A	-897.752
'070106040101020105	V	T	N	M	G	A	F	A	V	-897.751
'010103040201010105	L	T	D	M	A	A	W	A	V	-897.749
'010104070101010505	L	T	E	V	G	A	W	P	V	-897.748
'020107040101010502	F	T	Q	M	G	A	W	P	C	-897.747
'010103020201010101	L	T	D	I	A	A	W	A	A	-897.745
'040105050201020101	I	T	H	Q	A	A	F	A	A	-897.739
'070109010101010805	V	T	T	R	G	A	W	S	V	-897.736
'070309010201030101	V	M	T	R	A	A	Y	A	A	-897.733
'010303040201010501	L	M	D	M	A	A	W	P	A	-897.732
'010103070101010802	L	T	D	V	G	A	W	S	C	-897.732
'010409040101030105	L	S	T	M	G	A	Y	A	V	-897.732
'010303050101020105	L	M	D	Q	G	A	F	A	V	-897.731
'020107020101030102	F	T	Q	I	G	A	Y	A	C	-897.728
'010109070201020102	L	T	T	V	A	A	F	A	C	-897.726
'040406030101010105	I	S	N	K	G	A	W	A	V	-897.723
'040307060101010301	I	M	Q	T	G	A	W	G	A	-897.722
'040102060101020801	I	T	A	T	G	A	F	S	A	-897.72
'050302070101010501	M	M	A	V	G	A	W	P	A	-897.72
'010104060201030102	L	T	E	T	A	A	Y	A	C	-897.72
'010102060101010501	L	T	A	T	G	A	W	P	A	-897.711
'070108030101030105	V	T	S	K	G	A	Y	A	V	-897.71
'040109030101010402	I	T	T	K	G	A	W	K	C	-897.71
'070307070101010101	V	M	Q	V	G	A	W	A	A	-897.708
'040307020201010101	I	M	Q	I	A	A	W	A	A	-897.707

**'020107010201020501**	F	T	Q	R	A	A	F	P	A	-897.706
**'010105070101030205**	L	T	H	V	G	A	Y	E	V	-897.703
**'030107040101030501**	H	T	Q	M	G	A	Y	P	A	-897.7
**'090109060201030101**	Y	T	T	T	A	A	Y	A	A	-897.699
**'070105050101020101**	V	T	H	Q	G	A	F	A	A	-897.699
**'030105040101020501**	H	T	H	M	G	A	F	P	A	-897.698
**'040101040201020501**	I	T	G	M	A	A	F	P	A	-897.692
**'040109060201010102**	I	T	T	T	A	A	W	A	C	-897.691
**'040102010101010102**	I	T	A	R	G	A	W	A	C	-897.69
**'010206040101010105**	L	A	N	M	G	A	W	A	V	-897.689
**'070109070101030401**	V	T	T	V	G	A	Y	K	A	-897.688
**'070209010101020501**	V	A	T	R	G	A	F	P	A	-897.687
**'010309020101020501**	L	M	T	I	G	A	F	P	A	-897.685
**'030107060101010502**	H	T	Q	T	G	A	W	P	C	-897.683
**'090103020201020101**	Y	T	D	I	A	A	F	A	A	-897.68
**'040102020101010202**	I	T	A	I	G	A	W	E	C	-897.675
**'030105070101030501**	H	T	H	V	G	A	Y	P	A	-897.674
**'040404010101010405**	I	S	E	R	G	A	W	K	V	-897.674
**'070106070101010402**	V	T	N	V	G	A	W	K	C	-897.67
**'090105050101010201**	Y	T	H	Q	G	A	W	E	A	-897.663
**'070305010101030201**	V	M	H	R	G	A	Y	E	A	-897.66
**'040104070101020701**	I	T	E	V	G	A	F	R	A	-897.66
**'070108060101020301**	V	T	S	T	G	A	F	G	A	-897.66
**'070309030101030101**	V	M	T	K	G	A	Y	A	A	-897.653
**'070304010101030105**	V	M	E	R	G	A	Y	A	V	-897.647
**'040404060101010105**	I	S	E	T	G	A	W	A	V	-897.645
**'090305010101020105**	Y	M	H	R	G	A	F	A	V	-897.641
**'040105010101020305**	I	T	H	R	G	A	F	G	V	-897.641
**'050104050101010502**	M	T	E	Q	G	A	W	P	C	-897.64
**'040103070101010701**	I	T	D	V	G	A	W	R	A	-897.638
**'070103070101010205**	V	T	D	V	G	A	W	E	V	-897.637
**'070103070201020101**	V	T	D	V	A	A	F	A	A	-897.633

**'040305050101020101**	I	M	H	Q	G	A	F	A	A	-897.63
**'010103070101010501**	L	T	D	V	G	A	W	P	A	-897.63
**'070107020101020701**	V	T	Q	I	G	A	F	R	A	-897.626
**'020103020101020401**	F	T	D	I	G	A	F	K	A	-897.626
**'040105070201010501**	I	T	H	V	A	A	W	P	A	-897.621
**'020206040101010501**	F	A	N	M	G	A	W	P	A	-897.611
**'050107050201020101**	M	T	Q	Q	A	A	F	A	A	-897.61
**'040107010201010502**	I	T	Q	R	A	A	W	P	C	-897.61
**'050404020101010501**	M	S	E	I	G	A	W	P	A	-897.609
**'090105070101010305**	Y	T	H	V	G	A	W	G	V	-897.607
**'010108020101020205**	L	T	S	I	G	A	F	E	V	-897.596
**'040102060101010802**	I	T	A	T	G	A	W	S	C	-897.594
**'010103010101030102**	L	T	D	R	G	A	Y	A	C	-897.593
**'010103050101030501**	L	T	D	Q	G	A	Y	P	A	-897.592
**'040106010201010302**	I	T	N	R	A	A	W	G	C	-897.587
**'070105010101030602**	V	T	H	R	G	A	Y	Q	C	-897.586
**'050102050101020501**	M	T	A	Q	G	A	F	P	A	-897.584
**'020405070101010501**	F	S	H	V	G	A	W	P	A	-897.579
**'010209050101010305**	L	A	T	Q	G	A	W	G	V	-897.576
**'070308010201020101**	V	M	S	R	A	A	F	A	A	-897.574
**'070107070201010501**	V	T	Q	V	A	A	W	P	A	-897.565
**'070104070201030101**	V	T	E	V	A	A	Y	A	A	-897.564
**'050107010201030101**	M	T	Q	R	A	A	Y	A	A	-897.564
**'020108050101030501**	F	T	S	Q	G	A	Y	P	A	-897.564
**'010107070201030401**	L	T	Q	V	A	A	Y	K	A	-897.562
**'040304010101010605**	I	M	E	R	G	A	W	Q	V	-897.559
**'010105040101030705**	L	T	H	M	G	A	Y	R	V	-897.559
**'040104070101030101**	I	T	E	V	G	A	Y	A	A	-897.558
**'040204070101010701**	I	A	E	V	G	A	W	R	A	-897.55
**'020405040101010105**	F	S	H	M	G	A	W	A	V	-897.548
**'040104060101020601**	I	T	E	T	G	A	F	Q	A	-897.546
**'040103010101010505**	I	T	D	R	G	A	W	P	V	-897.542

**'050104020201020101**	M	T	E	I	A	A	F	A	A	-897.541
**'010305020201030101**	L	M	H	I	A	A	Y	A	A	-897.539
**'070305010101020101**	V	M	H	R	G	A	F	A	A	-897.539
**'070107050201010102**	V	T	Q	Q	A	A	W	A	C	-897.538
**'070304010101020501**	V	M	E	R	G	A	F	P	A	-897.536
**'070109050101010205**	V	T	T	Q	G	A	W	E	V	-897.536
**'070104040101030105**	V	T	E	M	G	A	Y	A	V	-897.532
**'030103050101030105**	H	T	D	Q	G	A	Y	A	V	-897.532
**'040102010101010501**	I	T	A	R	G	A	W	P	A	-897.53
**'040105040101010801**	I	T	H	M	G	A	W	S	A	-897.527
**'070103050101020501**	V	T	D	Q	G	A	F	P	A	-897.526
**'040105020201010301**	I	T	H	I	A	A	W	G	A	-897.521
**'010107040101030405**	L	T	Q	M	G	A	Y	K	V	-897.521
**'090105040201010601**	Y	T	H	M	A	A	W	Q	A	-897.515
**'010109030101020502**	L	T	T	K	G	A	F	P	C	-897.515
**'070403040101010105**	V	S	D	M	G	A	W	A	V	-897.512
**'090205020101010102**	Y	A	H	I	G	A	W	A	C	-897.51
**'050109060101020201**	M	T	T	T	G	A	F	E	A	-897.509
**'010407010101030505**	L	S	Q	R	G	A	Y	P	V	-897.507
**'040109010201030701**	I	T	T	R	A	A	Y	R	A	-897.504
**'020102040101020501**	F	T	A	M	G	A	F	P	A	-897.503
**'070405070101010102**	V	S	H	V	G	A	W	A	C	-897.492
**'040107010201010101**	I	T	Q	R	A	A	W	A	A	-897.489
**'070409010101020401**	V	S	T	R	G	A	F	K	A	-897.489
**'040105010201020103**	I	T	H	R	A	A	F	A	S	-897.487
**'090104070101030501**	Y	T	E	V	G	A	Y	P	A	-897.485
**'010103020101030405**	L	T	D	I	G	A	Y	K	V	-897.482
**'090109070101010502**	Y	T	T	V	G	A	W	P	C	-897.476
**'040209030101010105**	I	A	T	K	G	A	W	A	V	-897.474
**'090107070101010505**	Y	T	Q	V	G	A	W	P	V	-897.474
**'090107050101010502**	Y	T	Q	Q	G	A	W	P	C	-897.473
**'070309010101020105**	V	M	T	R	G	A	F	A	V	-897.463

**'070109040101010405**	V	T	T	M	G	A	W	K	V	-897.462
**'070105040101020801**	V	T	H	M	G	A	F	S	A	-897.462
**'040102010201020601**	I	T	A	R	A	A	F	Q	A	-897.458
**'090105020101010701**	Y	T	H	I	G	A	W	R	A	-897.457
**'040102040101010401**	I	T	A	M	G	A	W	K	A	-897.457
**'010109030201020501**	L	T	T	K	A	A	F	P	A	-897.455
**'040102010101030702**	I	T	A	R	G	A	Y	R	C	-897.454
**'050103050101010505**	M	T	D	Q	G	A	W	P	V	-897.451
**'090106040101010501**	Y	T	N	M	G	A	W	P	A	-897.448
**'090109010201030102**	Y	T	T	R	A	A	Y	A	C	-897.447
**'040304010101010402**	I	M	E	R	G	A	W	K	C	-897.444
**'040209010101010502**	I	A	T	R	G	A	W	P	C	-897.443
**'010108060101010502**	L	T	S	T	G	A	W	P	C	-897.443
**'010204050101030105**	L	A	E	Q	G	A	Y	A	V	-897.441
**'040408070101010102**	I	S	S	V	G	A	W	A	C	-897.427
**'050109010101030405**	M	T	T	R	G	A	Y	K	V	-897.424
**'050102060101010502**	M	T	A	T	G	A	W	P	C	-897.424
**'010109060101030505**	L	T	T	T	G	A	Y	P	V	-897.423
**'090202060101030101**	Y	A	A	T	G	A	Y	A	A	-897.422
**'040306020101010301**	I	M	N	I	G	A	W	G	A	-897.421
**'040309050101010601**	I	M	T	Q	G	A	W	Q	A	-897.419
**'040106030101020101**	I	T	N	K	G	A	F	A	A	-897.418
**'010202070101010205**	L	A	A	V	G	A	W	E	V	-897.414
**'070304020101010102**	V	M	E	I	G	A	W	A	C	-897.413
**'040208010101010802**	I	A	S	R	G	A	W	S	C	-897.41
**'070104070101020101**	V	T	E	V	G	A	F	A	A	-897.409
**'050106060201030101**	M	T	N	T	A	A	Y	A	A	-897.407
**'040106020101010402**	I	T	N	I	G	A	W	K	C	-897.404
**'090103040101010505**	Y	T	D	M	G	A	W	P	V	-897.403
**'090305040201010101**	Y	M	H	M	A	A	W	A	A	-897.402
**'040205010101010302**	I	A	H	R	G	A	W	G	C	-897.4
**'010403040201030101**	L	S	D	M	A	A	Y	A	A	-897.4

**'010203060101020201**	L	A	D	T	G	A	F	E	A	-897.395
**'010103020101030201**	L	T	D	I	G	A	Y	E	A	-897.394
**'050104020101010105**	M	T	E	I	G	A	W	A	V	-897.39
**'090209070101010102**	Y	A	T	V	G	A	W	A	C	-897.389
**'070103060101030105**	V	T	D	T	G	A	Y	A	V	-897.389
**'040109010101020701**	I	T	T	R	G	A	F	R	A	-897.387
**'040108010101030401**	I	T	S	R	G	A	Y	K	A	-897.386
**'020105050101030105**	F	T	H	Q	G	A	Y	A	V	-897.384
**'020109010101020502**	F	T	T	R	G	A	F	P	C	-897.383
**'040207070101010102**	I	A	Q	V	G	A	W	A	C	-897.38
**'040302050101010102**	I	M	A	Q	G	A	W	A	C	-897.379
**'010309020101010505**	L	M	T	I	G	A	W	P	V	-897.378
**'050109060101010501**	M	T	T	T	G	A	W	P	A	-897.375
**'070107070101020401**	V	T	Q	V	G	A	F	K	A	-897.374
**'020106060101020102**	F	T	N	T	G	A	F	A	C	-897.373
**'040108020101010302**	I	T	S	I	G	A	W	G	C	-897.371
**'040305010101030401**	I	M	H	R	G	A	Y	K	A	-897.37
**'040106010201010205**	I	T	N	R	A	A	W	E	V	-897.367
**'030107010101020102**	H	T	Q	R	G	A	F	A	C	-897.366
**'090405040101020101**	Y	S	H	M	G	A	F	A	A	-897.366
**'090406020101010501**	Y	S	N	I	G	A	W	P	A	-897.358
**'070204010101030102**	V	A	E	R	G	A	Y	A	C	-897.357
**'040102050101020105**	I	T	A	Q	G	A	F	A	V	-897.356
**'040307060101010103**	I	M	Q	T	G	A	W	A	S	-897.355
**'040402020101010501**	I	S	A	I	G	A	W	P	A	-897.354
**'010107070101030302**	L	T	Q	V	G	A	Y	G	C	-897.351
**'010205040101010605**	L	A	H	M	G	A	W	Q	V	-897.351
**'010103060101030805**	L	T	D	T	G	A	Y	S	V	-897.348
**'040207010201010501**	I	A	Q	R	A	A	W	P	A	-897.348
**'020109020101020501**	F	T	T	I	G	A	F	P	A	-897.346
**'070308010101030501**	V	M	S	R	G	A	Y	P	A	-897.343
**'010405010101020305**	L	S	H	R	G	A	F	G	V	-897.342

**'040208020101030101**	I	A	S	I	G	A	Y	A	A	-897.342
**'010205040101020102**	L	A	H	M	G	A	F	A	C	-897.331
**'040101040201030102**	I	T	G	M	A	A	Y	A	C	-897.331
**'020403010201020101**	F	S	D	R	A	A	F	A	A	-897.326
**'040105030101030101**	I	T	H	K	G	A	Y	A	A	-897.324
**'010103060201020401**	L	T	D	T	A	A	F	K	A	-897.323
**'010105020201030501**	L	T	H	I	A	A	Y	P	A	-897.32
**'010305070101010205**	L	M	H	V	G	A	W	E	V	-897.319
**'040309010101010301**	I	M	T	R	G	A	W	G	A	-897.316
**'010207070101010501**	L	A	Q	V	G	A	W	P	A	-897.312
**'070104070101010105**	V	T	E	V	G	A	W	A	V	-897.311
**'050105060101030501**	M	T	H	T	G	A	Y	P	A	-897.31
**'040105060101020201**	I	T	H	T	G	A	F	E	A	-897.31
**'090309060101010501**	Y	M	T	T	G	A	W	P	A	-897.308
**'070107010201020201**	V	T	Q	R	A	A	F	E	A	-897.299
**'070106060101010705**	V	T	N	T	G	A	W	R	V	-897.296
**'010109060101020102**	L	T	T	T	G	A	F	A	C	-897.291
**'010302040101010505**	L	M	A	M	G	A	W	P	V	-897.288
**'010303040101010202**	L	M	D	M	G	A	W	E	C	-897.287
**'020204070101010501**	F	A	E	V	G	A	W	P	A	-897.286
**'070108010101020805**	V	T	S	R	G	A	F	S	V	-897.284
**'040308010101010205**	I	M	S	R	G	A	W	E	V	-897.277
**'020105020101030101**	F	T	H	I	G	A	Y	A	A	-897.272
**'090105010201020105**	Y	T	H	R	A	A	F	A	V	-897.268
**'040305010101020103**	I	M	H	R	G	A	F	A	S	-897.265
**'090105050101030101**	Y	T	H	Q	G	A	Y	A	A	-897.264
**'010303070101020501**	L	M	D	V	G	A	F	P	A	-897.264
**'070407010101010505**	V	S	Q	R	G	A	W	P	V	-897.262
**'040208010101010405**	I	A	S	R	G	A	W	K	V	-897.256
**'040204010101010101**	I	A	E	R	G	A	W	A	A	-897.255
**'010103020101010302**	L	T	D	I	G	A	W	G	C	-897.251
**'040103040201010201**	I	T	D	M	A	A	W	E	A	-897.25

**'010403010101020202**	L	S	D	R	G	A	F	E	C	-897.249
**'090205010201030101**	Y	A	H	R	A	A	Y	A	A	-897.249
**'070302030101010101**	V	M	A	K	G	A	W	A	A	-897.241
**'030109020101010302**	H	T	T	I	G	A	W	G	C	-897.241
**'040209060101010401**	I	A	T	T	G	A	W	K	A	-897.239
**'090105050101010105**	Y	T	H	Q	G	A	W	A	V	-897.233
**'040108060101020101**	I	T	S	T	G	A	F	A	A	-897.231
**'050104040201010105**	M	T	E	M	A	A	W	A	V	-897.227
**'010108010101030505**	L	T	S	R	G	A	Y	P	V	-897.227
**'040405040101010101**	I	S	H	M	G	A	W	A	A	-897.226
**'050109020101010405**	M	T	T	I	G	A	W	K	V	-897.225
**'050109030101030102**	M	T	T	K	G	A	Y	A	C	-897.224
**'070108030101010205**	V	T	S	K	G	A	W	E	V	-897.223
**'070308020101010105**	V	M	S	I	G	A	W	A	V	-897.22
**'070105050201010105**	V	T	H	Q	A	A	W	A	V	-897.219
**'010403020101010401**	L	S	D	I	G	A	W	K	A	-897.219
**'040304010201010501**	I	M	E	R	A	A	W	P	A	-897.217
**'030103010201030102**	H	T	D	R	A	A	Y	A	C	-897.215
**'040106010101030402**	I	T	N	R	G	A	Y	K	C	-897.214
**'040104010101020503**	I	T	E	R	G	A	F	P	S	-897.211
**'070109030101030301**	V	T	T	K	G	A	Y	G	A	-897.208
**'050104070101020102**	M	T	E	V	G	A	F	A	C	-897.208
**'040305060101010701**	I	M	H	T	G	A	W	R	A	-897.208
**'070105040101010101**	V	T	H	M	G	A	W	A	A	-897.206
**'020105040201020101**	F	T	H	M	A	A	F	A	A	-897.205
**'040408010101020102**	I	S	S	R	G	A	F	A	C	-897.203
**'040103060201010501**	I	T	D	T	A	A	W	P	A	-897.203
**'020105040101010202**	F	T	H	M	G	A	W	E	C	-897.198
**'010103060101030701**	L	T	D	T	G	A	Y	R	A	-897.195
**'090102010101020502**	Y	T	A	R	G	A	F	P	C	-897.193
**'040108050101030701**	I	T	S	Q	G	A	Y	R	A	-897.192
**'070105010201030301**	V	T	H	R	A	A	Y	G	A	-897.19

**'090105050101020701**	Y	T	H	Q	G	A	F	R	A	-897.189
**'050106050101010205**	M	T	N	Q	G	A	W	E	V	-897.184
**'070202010101020102**	V	A	A	R	G	A	F	A	C	-897.178
**'010108060101030501**	L	T	S	T	G	A	Y	P	A	-897.178
**'040306060101010401**	I	M	N	T	G	A	W	K	A	-897.177
**'040408010101030105**	I	S	S	R	G	A	Y	A	V	-897.177
**'070105050101010102**	V	T	H	Q	G	A	W	A	C	-897.174
**'040107060101030701**	I	T	Q	T	G	A	Y	R	A	-897.172
**'050102050101010305**	M	T	A	Q	G	A	W	G	V	-897.172
**'070207010101030105**	V	A	Q	R	G	A	Y	A	V	-897.171
**'040104020101020801**	I	T	E	I	G	A	F	S	A	-897.171
**'040102060201010105**	I	T	A	T	A	A	W	A	V	-897.171
**'070406010101020501**	V	S	N	R	G	A	F	P	A	-897.17
**'040106070101010605**	I	T	N	V	G	A	W	Q	V	-897.168
**'070107040201010201**	V	T	Q	M	A	A	W	E	A	-897.162
**'010405010101020502**	L	S	H	R	G	A	F	P	C	-897.159
**'030102020101030501**	H	T	A	I	G	A	Y	P	A	-897.159
**'070103040101010605**	V	T	D	M	G	A	W	Q	V	-897.159
**'020103030101020102**	F	T	D	K	G	A	F	A	C	-897.158
**'040301070101030501**	I	M	G	V	G	A	Y	P	A	-897.154
**'050108020101010205**	M	T	S	I	G	A	W	E	V	-897.154
**'070109030101010501**	V	T	T	K	G	A	W	P	A	-897.154
**'070209040101010301**	V	A	T	M	G	A	W	G	A	-897.152
**'070107070101030701**	V	T	Q	V	G	A	Y	R	A	-897.152
**'040103070101010805**	I	T	D	V	G	A	W	S	V	-897.151
**'040107070101030601**	I	T	Q	V	G	A	Y	Q	A	-897.151
**'090103050101020102**	Y	T	D	Q	G	A	F	A	C	-897.151
**'010404070101010305**	L	S	E	V	G	A	W	G	V	-897.151
**'040109060101010205**	I	T	T	T	G	A	W	E	V	-897.15
**'020105040201010102**	F	T	H	M	A	A	W	A	C	-897.15
**'010105010201020305**	L	T	H	R	A	A	F	G	V	-897.15
**'090109040101030701**	Y	T	T	M	G	A	Y	R	A	-897.146

**'070108050101010505**	V	T	S	Q	G	A	W	P	V	-897.144
**'010203050101010501**	L	A	D	Q	G	A	W	P	A	-897.142
**'090105070101020801**	Y	T	H	V	G	A	F	S	A	-897.141
**'040105020101010203**	I	T	H	I	G	A	W	E	S	-897.141
**'020307060101010501**	F	M	Q	T	G	A	W	P	A	-897.141
**'010407060101010305**	L	S	Q	T	G	A	W	G	V	-897.14
**'040104010201020701**	I	T	E	R	A	A	F	R	A	-897.138
**'040105050101010101**	I	T	H	Q	G	A	W	A	A	-897.137
**'070304030101010105**	V	M	E	K	G	A	W	A	V	-897.136
**'070103010201010505**	V	T	D	R	A	A	W	P	V	-897.133
**'070307050101030101**	V	M	Q	Q	G	A	Y	A	A	-897.132
**'010407030101030501**	L	S	Q	K	G	A	Y	P	A	-897.132
**'050109040101010705**	M	T	T	M	G	A	W	R	V	-897.132
**'040407020101010701**	I	S	Q	I	G	A	W	R	A	-897.13
**'070105040101010602**	V	T	H	M	G	A	W	Q	C	-897.129
**'010102030101020501**	L	T	A	K	G	A	F	P	A	-897.129
**'010207050101020501**	L	A	Q	Q	G	A	F	P	A	-897.129
**'010404020101010505**	L	S	E	I	G	A	W	P	V	-897.129
**'090103060101010201**	Y	T	D	T	G	A	W	E	A	-897.126
**'040109040101010201**	I	T	T	M	G	A	W	E	A	-897.124
**'010203070101010805**	L	A	D	V	G	A	W	S	V	-897.124
**'020105060101030701**	F	T	H	T	G	A	Y	R	A	-897.119
**'090108020101030105**	Y	T	S	I	G	A	Y	A	V	-897.119
**'070106010101030705**	V	T	N	R	G	A	Y	R	V	-897.117
**'010409040101030501**	L	S	T	M	G	A	Y	P	A	-897.116
**'040306030101010102**	I	M	N	K	G	A	W	A	C	-897.114
**'090305010201020101**	Y	M	H	R	A	A	F	A	A	-897.113
**'010107030201030501**	L	T	Q	K	A	A	Y	P	A	-897.112
**'050109020101010503**	M	T	T	I	G	A	W	P	S	-897.111
**'040104020101020103**	I	T	E	I	G	A	F	A	S	-897.108
**'010203020101030401**	L	A	D	I	G	A	Y	K	A	-897.105
**'090205010101010502**	Y	A	H	R	G	A	W	P	C	-897.103

**'070103050101010302**	V	T	D	Q	G	A	W	G	C	-897.102
**'070207040101020101**	V	A	Q	M	G	A	F	A	A	-897.101
**'070409010101010501**	V	S	T	R	G	A	W	P	A	-897.1
**'040104040101020101**	I	T	E	M	G	A	F	A	A	-897.094
**'010107040201020801**	L	T	Q	M	A	A	F	S	A	-897.094
**'010303040101030501**	L	M	D	M	G	A	Y	P	A	-897.094
**'050102020101010501**	M	T	A	I	G	A	W	P	A	-897.091
**'070405010201020101**	V	S	H	R	A	A	F	A	A	-897.091
**'090206020101030101**	Y	A	N	I	G	A	Y	A	A	-897.09
**'040105060101010103**	I	T	H	T	G	A	W	A	S	-897.09
**'090405020101010105**	Y	S	H	I	G	A	W	A	V	-897.088
**'070308040101030101**	V	M	S	M	G	A	Y	A	A	-897.084
**'040106050201010105**	I	T	N	Q	A	A	W	A	V	-897.076
**'040109030101030801**	I	T	T	K	G	A	Y	S	A	-897.073
**'090104060101010501**	Y	T	E	T	G	A	W	P	A	-897.073
**'010108070101030702**	L	T	S	V	G	A	Y	R	C	-897.072
**'070106010201020101**	V	T	N	R	A	A	F	A	A	-897.072
**'010103040101030105**	L	T	D	M	G	A	Y	A	V	-897.07
**'010402010101030502**	L	S	A	R	G	A	Y	P	C	-897.07
**'090103040101020501**	Y	T	D	M	G	A	F	P	A	-897.066
**'010109020201020301**	L	T	T	I	A	A	F	G	A	-897.065
**'040309010101010503**	I	M	T	R	G	A	W	P	S	-897.064
**'070209030101010102**	V	A	T	K	G	A	W	A	C	-897.063
**'070104060201020101**	V	T	E	T	A	A	F	A	A	-897.062
**'010107020101010305**	L	T	Q	I	G	A	W	G	V	-897.061
**'010206040101030501**	L	A	N	M	G	A	Y	P	A	-897.056
**'040205010101010503**	I	A	H	R	G	A	W	P	S	-897.049
**'070108060201010105**	V	T	S	T	A	A	W	A	V	-897.048
**'040105070101010301**	I	T	H	V	G	A	W	G	A	-897.043
**'010404010201020501**	L	S	E	R	A	A	F	P	A	-897.042
**'010103040101010702**	L	T	D	M	G	A	W	R	C	-897.041
**'020304020101010105**	F	M	E	I	G	A	W	A	V	-897.039

**'010107020101020505**	L	T	Q	I	G	A	F	P	V	-897.035
**'020103010101020505**	F	T	D	R	G	A	F	P	V	-897.035
**'010102050201030102**	L	T	A	Q	A	A	Y	A	C	-897.033
**'050304040101010501**	M	M	E	M	G	A	W	P	A	-897.033
**'010103040101010305**	L	T	D	M	G	A	W	G	V	-897.031
**'010106070101010305**	L	T	N	V	G	A	W	G	V	-897.03
**'070107020101030601**	V	T	Q	I	G	A	Y	Q	A	-897.03
**'070305020101010201**	V	M	H	I	G	A	W	E	A	-897.028
**'040306010201010105**	I	M	N	R	A	A	W	A	V	-897.023
**'090105010101020503**	Y	T	H	R	G	A	F	P	S	-897.023
**'070106070101010501**	V	T	N	V	G	A	W	P	A	-897.014
**'050106070201020101**	M	T	N	V	A	A	F	A	A	-897.012
